# Fusion-driven oncogenic programs shape the immune landscape in translocation renal cell carcinoma

**DOI:** 10.1038/s41467-026-76858-w

**Published:** 2026-08-26

**Authors:** Prathyusha Konda, Alexandra J. Wakolbinger, Yantong Cui, Gabriel Roberti de Oliveira, Yasmin L. Nabil, Cary N. Weiss, Maxwell D. Seager, Riva Deodhar, Jiao Li, Sayed Matar, Jinyu Wang, Jack Horst, Sabrina Y. Camp, Aseman B. Sheshdeh, Jonathan L. Hecht, David J. Einstein, Yashika Rustagi, Anwesha Nag, Aaron R. Thorner, Cheng-Zhong Zhang, Eliezer M. Van Allen, Sabina Signoretti, Toni K. Choueiri, Srinivas R. Viswanathan

**Affiliations:** 1 Department of Medical Oncology, Dana-Farber Cancer Institute, Boston, MA USA; 2 Department of Medicine, Harvard Medical School, Boston, MA USA; 3 Department of Pathology, Brigham and Women’s Hospital, Boston, MA USA; 4 Department of Pediatric Oncology, Dana-Farber Cancer Institute, Boston, MA USA; 5 Division of Hematology/Oncology, Boston Children’s Hospital; Harvard Medical School, Boston, MA USA; 6 Department of Biomedical Informatics, Dana-Farber Cancer Institute, Boston, MA USA; 7 Department of Pathology, Beth Israel Deaconess Medical Center, Boston, MA USA; 8 Division of Medical Oncology, Beth Israel Deaconess Medical Center, Boston, MA USA; 9 Broad Institute of MIT and Harvard, Cambridge, MA USA; 10 Department of Medicine, Brigham and Women’s Hospital, Boston, MA USA

**Keywords:** Renal cell carcinoma, Cancer genomics

## Abstract

Renal cell carcinomas (RCC) comprise multiple molecularly distinct cancers but most are treated empirically with therapies designed for clear cell RCC (ccRCC), the most common subtype, due to incomplete understanding of subtype-specific biology. We analyzed single-cell transcriptomes and chromatin accessibility profiles from translocation renal cell carcinoma (tRCC), an aggressive RCC defined by oncogenic *TFE3* gene fusions. We show that, despite arising from a proximal tubule cell of origin similar to ccRCC, tRCCs display distinct oncogenic programs and an immunosuppressive tumor microenvironment (TME). tRCCs exhibit six conserved tumor meta-programs, including epithelial-mesenchymal transition (EMT) and proximal tubule identity programs whose balance is regulated by TFE3 fusion activity. The fusion-driven EMT program drives a suppressive TME marked by progenitor-exhausted CD8 + T cells, anti-inflammatory SPP1+ macrophages, and matrix-associated fibroblasts. Our findings highlight unique TFE3 fusion-driven biology in tRCC, explaining its reduced immunotherapy responsiveness relative to ccRCC, and suggesting strategies for targeting fusion-driven oncogenic programs and TME reprogramming.

## Abstract (web summary)

Currently, there is limited understanding of the biology for translocation renal cell carcinoma (RCC), a rare RCC subtype defined by oncogenic TFE3 gene fusions. Here, the authors use multi-omics profiling to show that TFE3 fusions drive distinct tumor programs and an immunosuppressive microenvironment linked to reduced immunotherapy response.

## Introduction

Kidney cancer comprises dozens of different subtypes, each with unique molecular and clinical characteristics. To date, most discovery biology research in kidney cancer has focused on clear-cell renal cell carcinoma (ccRCC), which constitutes approximately 75% of renal cell carcinomas in adults^1,2^. ccRCC is characterized by a pathognomonic inactivation of the Von Hippel-Lindau (*VHL*) tumor suppressor gene, which results in the uncontrolled activation of hypoxia-inducible transcription factors (HIFs)^3–6^. Accordingly, therapies aimed at targeting hypoxia signaling, along with immunotherapies, have significantly advanced the treatment of ccRCC^7,8^. However, these treatments are generally less effective against other forms of kidney cancer, which are driven by different biological mechanisms^9,10^.

Translocation RCC (tRCC) is a rare and aggressive subtype of RCC defined by oncogenic gene fusions involving a transcription factor in the MiT/TFE family, usually TFE3^11–13^. tRCC comprises approximately 1–5% of RCCs in adults and about 50% of RCCs in children; however, its true incidence may be underestimated due to frequent histologic misclassification as other, more common, subtypes of kidney cancer^14–16^. There is no established standard of care treatment specifically for tRCC, and responses to immune checkpoint inhibitors (ICI) and vascular endothelial growth factor (VEGF)-targeted kinase inhibitors are typically more modest than in ccRCC^17–20^. Moreover, an incomplete molecular understanding of tRCC has hindered the ability to potentiate these therapies and to identify new mechanism-informed targets in this subtype.

Prior molecular characterization studies of tRCC have been limited in scale due to this cancer's rarity. Nonetheless, recent whole exome sequencing and whole genome sequencing studies have revealed that tRCCs have a low mutational rate and harbor few recurrent genomic alterations apart from the driver MiT/TFE gene fusion^12,21–26^. Despite this quiet and homogeneous genomic profile, tRCCs have been proposed to comprise multiple transcriptional and proteomic subtypes, though these studies have relied on bulk profiling and/or have identified different sets of conserved molecular programs^22,23,27–29^. Indeed, multiple pathways have been identified as activated in tRCC, including the antioxidant response, mTOR signaling, oxidative phosphorylation, autophagy, epithelial-mesenchymal transition (EMT), Ret signaling and Wnt signaling, amongst others^12,22,23,30–34^.

To date, malignant programs in tRCC have not been systematically studied at single-cell resolution, nor have they been linked to specific components of the tumor ecosystem in tRCC. Therefore, how oncogenic TFE3 fusions direct malignant programs, shape the immune landscape, and influence therapy response in tRCC remains poorly defined.

In this study, we assembled a cohort of 20 tRCC samples which were profiled using single-nucleus RNA sequencing (snRNA-seq), along with single nucleus ATAC sequencing (snATAC-seq), spatial transcriptomic analysis, and T Cell Receptor (TCR) sequencing. By integrating 128 bulk transcriptomic tRCC profiles from prior studies^22,23,35–37^ and a systematic comparison with 1311 published ccRCC bulk and 32 single-cell transcriptional profiles^35,36,38^, we present a detailed picture of how TFE3 fusions drive malignancy and contribute to immunotherapy resistance, while nominating biologically relevant therapeutic entry points in this aggressive cancer.

## Results

### Single nucleus sequencing of tRCC

**Fig. 1:**
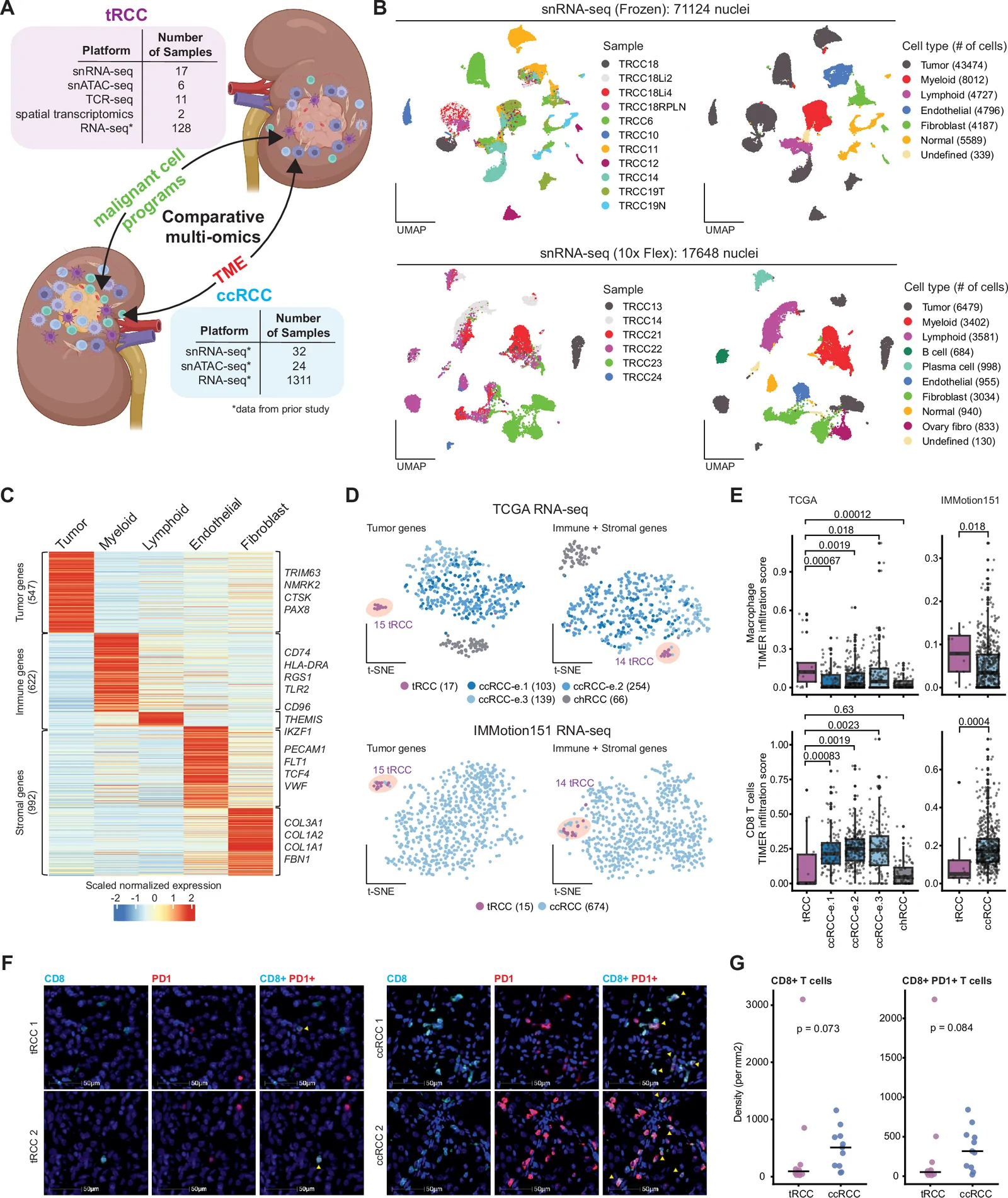
tRCCs and ccRCCs have distinct cellular landscapes. **A** Schematic representation of study design, including sample numbers for tRCC and ccRCC cohorts analyzed in the study. **B** Uniform manifold projections (UMAP) of nuclei from tRCC samples annotated by Sample ID (left) and cell lineage (right) in snRNA-seq frozen (top) or 10x-flex (bottom) cohorts. **C** Heatmap of genes highly expressed in tumor, immune, or stromal cells in tRCC based on snRNA-seq data (frozen cohort). **D** Projection of genes from (**C**) onto bulk RNA-Seq RCC datasets (top, TCGA; bottom, IMmotion151), with t-SNE plots showing separation of subtypes based on either tumor (left) or microenvironmental (immune + stromal, right) gene signatures. **E** Box plots displaying estimated proportions of immune cell types in various RCC subtypes, derived from immune deconvolution analysis of bulk RNA-Seq datasets (TCGA: *N* = 17 tRCC, 103 ccRCC-e.1, 254 ccRCC-e.2, 139 ccRCC-e.3, 66 chRCC; IMMotion151: *N* = 15 tRCC, 674 ccRCC). Data is represented as median with 25th and 75th percentiles defining the box limits. *P* values are calculated using two-sided Wilcoxon rank-sum tests. **F** Multiplex immunofluorescence for CD8 and PD1 in representative tRCC and ccRCC cases. Yellow arrows indicate CD8 + PD1+ cells. **G** Quantification of CD8 + T cell density (left) and CD8 + PD1 + T cell density (right) in tRCC (*N* = 10) and ccRCC (*N* = 11) cases. *P*-values are calculated by two-sided Wilcoxon’s rank-sum test.

We assembled a cohort of 20 tRCC samples from 15 patients, of which 17 samples (16 tumors, 1 adjacent normal) underwent single-nucleus profiling via one of three platforms: (1) 10x Genomics 3′ snRNA-Seq (6 fresh frozen samples); (2) 10x 3′ multiome (5 fresh frozen samples); (3) 10x-Flex fixed RNA-Seq (6 FFPE samples) (Figs. 1A and S1A). For comparative analysis, we uniformly reprocessed data from 32 snRNA-seq and 24 snATAC-seq samples of ccRCC profiled via the 3′ multiome platform in a prior study^38^. We also aggregated 128 tRCC and 1311 ccRCC bulk transcriptomes from across 5 published studies for validation analyses^22,23,35–37^ (Fig. 1A).

Our snRNA-Seq cohort was female predominant (8 females, 4 males), consistent with the known female sex-bias in tRCC^21^. Four different *TFE3* fusion partner genes were represented (*PRCC*, *SFPQ*, *MED15*, and *LUC7L3*). Given the rarity of tRCC, the full snRNA-seq cohort was heterogeneous with respect to tumor site (11 primary, 5 metastases; including 3 metastatic site samples from one patient) and treatment history (8 treatment-naïve samples from 8 unique patients and 8 with prior systemic therapy from 6 unique patients) (Fig. S1A and Supplementary Data 1).

Unsupervised clustering of snRNA-seq data from our tRCC cohort revealed robust separation of non-malignant cell types such as endothelial cells, fibroblasts, immune populations, and normal kidney cells, regardless of patient origin or platform. By contrast, malignant cells segregated by patient, suggesting a degree of intertumoral heterogeneity (Figs. 1B and S1B, C). To additionally confirm malignant cell identity and assess large-scale genomic alterations, we performed inferCNV analysis across the frozen cohort (Fig. S2A). Inferred chromosomal copy-number profiles were consistent within malignant cells of individual tumors and showed concordance with copy number alterations identified in matched whole-genome sequencing data from the same patients in our previous study^21^. These findings are broadly consistent with a lack of subclonal genetic alterations in tRCC^12,21^. While frozen and FFPE snRNA-seq platforms yielded relatively comparable feature distributions, cell type proportions differed between platforms (likely due to differences in sample preparation), leading us to analyze the two cohorts separately (Fig. S1D–F).

### tRCC exhibits distinctive tumor and microenvironment-derived gene expression

After assigning cell types in our tRCC snRNA-seq cohort, we identified genes differentially expressed in the tumor, immune, and stromal compartments (Fig. 1C). We then mapped these lineage-specific signatures onto two bulk RNA-seq datasets that included tRCCs and other kidney cancers (TCGA; IMmotion151)^35,36^. tRCC transcriptomes segregated from both ccRCC and chromophobe RCC (chRCC) when clustered based on tumor genes (Fig. 1D, left) and when clustered by immune/stromal genes (Fig. 1D, right) (Note: papillary RCCs were excluded from both cohorts due to their molecularly heterogeneous nature^39,40^). Immune deconvolution analyses using TIMER^41^, which accounts for tumor purity, inferred significantly increased macrophage infiltration in tRCC compared to ccRCC and chRCC, as well as significantly reduced CD8 + T cells in tRCC relative to ccRCC, across both the TCGA and IMmotion151 cohorts (Fig. 1E). These findings were validated at the protein level by multiplex immunofluorescence on a cohort of tRCC (*N* = 10) and ccRCC (*N* = 11) cases, which confirmed significantly lower CD8+ and CD8 + PD1+ populations in tRCC (Fig. 1F). CIBERSORTx^42^ absolute mode analysis, which provides granular macrophage subtype estimates, showed concordant results for CD8 + T cells but yielded partially discordant results for macrophage subpopulations (Fig. S2B–D). Notably, CIBERSORTx indicated a higher M2/M1 ratio in tRCC compared to ccRCC (Fig. S2B). The differences in the underlying assumptions and sensitivity to tumor purity in these two methods provided an impetus to further characterize the macrophage compartment at single-cell resolution in this study. Overall, these analyses suggest that the unique clinical features of tRCC may be linked to distinctions both in malignant programs as well as in the surrounding stromal and immune microenvironment.

### Divergent transcriptional and epigenetic programs in tRCC and ccRCC

**Fig. 2:**
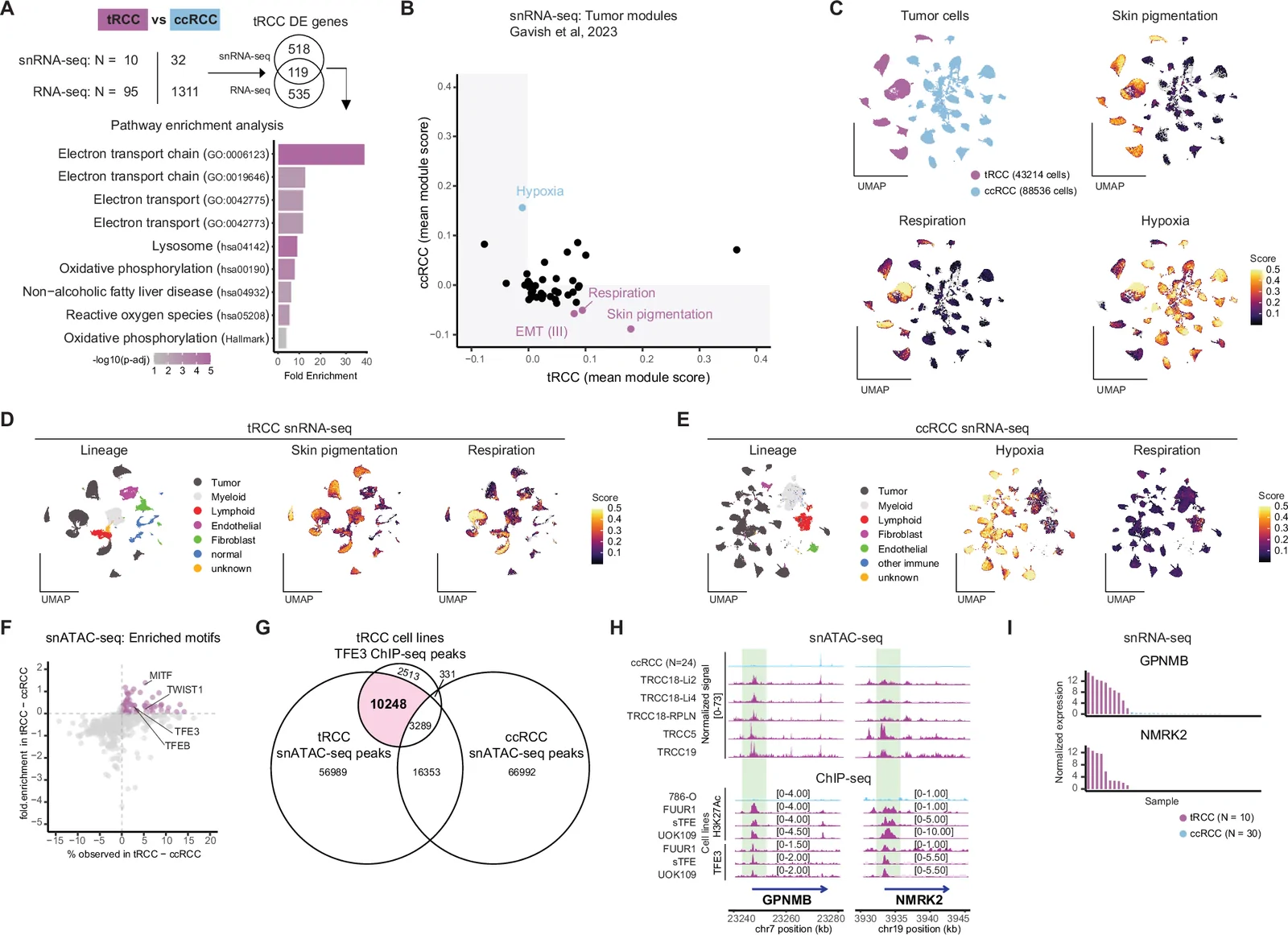
Divergent tumor transcriptional profiles in tRCC and ccRCC. **A** Differential expression analysis of tRCC vs ccRCC tumors using snRNA-seq (Frozen) and RNA-seq data, highlighting top enriched pathways in tRCC tumors identified based on gene-ontology. One-sided hypergeometric over-representation test values along with adjusted *p*-value corrected via Benjamini-hochberg method are represented. **B** Scatterplot of program enrichment in tRCC vs ccRCC tumor cells from snRNA-seq data (Frozen), based on consensus meta-programs established in a recent pan-cancer study^45^. **C** UMAPs representing tumor cells from tRCC and ccRCC snRNA-seq datasets (top left), colored by: skin pigmentation program module score (top right), respiration program module score (bottom left), and hypoxia program module score (bottom right). **D** UMAPs showing all lineages in the tRCC snRNA-seq dataset (left) colored by: skin pigmentation module score (middle) and respiration module score (right). **E** UMAPs depicting all cellular lineages in the ccRCC snRNA-seq dataset (left) colored by: hypoxia module score (middle) and respiration module score (right). **F** Scatterplot of motifs enriched in tRCC and ccRCC tumor cells from snATAC-seq data. *X*-axis represents the difference in percent motif observed in tRCC vs. ccRCC tumor cells and *Y*-axis represents the difference in fold enrichment of each motif in tRCC vs. ccRCC. tRCC-enriched motifs are highlighted in pink. **G** Venn diagram showing the intersection of peaks from tRCC snATAC-seq data (tumor cells), TFE3 ChIP Seq from tRCC cell lines, snATAC-seq from ccRCC tumor cells. Peaks specific to tRCC are highlighted in pink. **H** Peaks at the loci for two tRCC-marker genes (*GPNMB* and *NMRK2*) from: snATAC-seq of tRCC and ccRCC tumors (top) and ChIP-seq of H3K27ac or TFE3 in tRCC (FUUR1, sTFE, UOK109) or ccRCC (786-O) cell lines (bottom). **I** Waterfall plot representing normalized expression of tRCC-specific genes, *GPNMB* and *NMRK2*, in each sample of tRCC and ccRCC tumors profiled via snRNA-seq.

To characterize the malignant programs that typify tRCC, we performed differential gene expression analysis on malignant cells from our tRCC snRNA-seq cohort (*N* = 10 frozen samples only) and a previously published snRNA-seq cohort of ccRCC (*N* = 32)^38^. We intersected tRCC-enriched genes from this analysis with differential gene expression performed on a much larger bulk RNA-seq cohorts for tRCC (*N* = 95^22,35,36^) and ccRCC (*N* = 1311^35,36^) (Fig. 2A and Supplementary Data 2). This consensus set of genes upregulated in tRCC tumors was strongly enriched for pathways associated with reactive oxygen species handling, oxidative phosphorylation, and the electron transport chain, consistent with our recent finding that these pathways are under direct transcriptional control of the TFE3 fusion^30^. tRCCs also exhibited an upregulation of the lysosomal pathway, consistent with the canonical role for MiT/TFE genes as master regulators of lysosomal biogenesis^43^ (Fig. 2A). By contrast, ccRCC tumors displayed upregulation of hypoxia and glycolysis related genes, in keeping with the well-established role of HIF stabilization and Warburg-like metabolism in ccRCC^5,44^ (Fig. S2E).

We also evaluated cancer cell states previously defined in pan-cancer single cell studies^45,46^. Consistent with our above analyses and recent work suggesting metabolically divergent states in tRCC and ccRCC^30,31^, a respiration program was enriched in tRCC while a hypoxia program was enriched in ccRCC (Figs. 2B–E and S2F). tRCC malignant cells also displayed enrichment of EMT and skin pigmentation transcriptional programs, the latter being originally described as an MITF-driven hallmark module in melanoma^45^ and reflective of tRCC being driven by an MiT/TFE family transcription factor (Fig. 2B, C). Both the pigmentation and respiration programs were also enriched in tumor cells relative to non-malignant cells, with the exception of some activity of the pigmentation program in myeloid cells (consistent with a role for TFE3 in macrophages^47–49^) and some activity of the respiration program in lymphoid cells (consistent with resting T lymphocytes preferring aerobic respiration^50^) (Fig. 2D).

To assess whether these transcriptional differences were driven by distinct chromatin accessibility profiles in tRCC vs. ccRCC malignant cells, we interrogated our tRCC single snATAC-seq dataset (23542 nuclei from 5 samples) dataset together with a published ccRCC snATAC-Seq dataset (119130 nuclei from 24 samples^38^). As expected, transcription factor motif enrichment analysis identified increased accessibility of MiT/TFE family members in tRCC vs. ccRCC (Fig. 2F). We also observed tRCC-specific enrichment of a TWIST1 motif (master regulator of EMT^51^), consistent with our observation of an EMT transcriptional program in tRCC malignant cells.

To determine whether regions of chromatin accessibility enriched in tRCC marked fusion target genes, we next intersected TFE3 fusion binding sites as determined by ChIP-Seq on tRCC cell lines^30^ with snATAC-seq peaks in the tRCC and ccRCC cohorts. A majority of TFE3 fusion ChIP-Seq peaks overlapped with tRCC-specific ATAC-Seq peaks (10248 peaks, hypergeometric test *p* = 6e-85) (Fig. 2G). These regions exhibited increased H3K27Ac signal and TFE3 occupancy of the canonical TFE3 target genes, *GPNMB*^33^ and *NMRK2*^52,53^ (Fig. 2H). Correspondingly, these fusion target genes were also strongly upregulated at the RNA level in tRCC tumors (Fig. 2I), highlighting the direct linkage between chromatin accessibility and gene expression downstream of TFE3 fusions.

### Cell-type identification points to a common cell of origin for tRCC and ccRCC

The stark transcriptional and epigenetic differences between tRCC and ccRCC malignant cells may reflect their distinct genetic drivers, a distinct cellular origin, or both. Kidney cancers are known to be cellularly diverse, comprising dozens of histologies originating from various cell types along the length of the nephron^54^. For example, the cell of origin for ccRCC has been proposed as proximal tubule (PT), while collecting duct intercalated cells are proposed as the cell of origin for chRCC, and PT for papillary RCC^54–56^. Recent studies have proposed a PT cell origin for tRCC^56,57^; however, owing to the rarity of tRCC, these conclusions were based on bulk transcriptome analysis or on single-cell profiling of a single sample and not mapped to the resolution of a subpopulation within PT cells. Therefore, we sought to comprehensively define the cell of origin in tRCC.

We leveraged a recently published snRNA-seq atlas of normal adult human kidney^58^ to classify cell types within a tumor-adjacent normal sample from patient TRCC19, identifying 13 distinct cell types representing all segments of the nephron (Fig. S3A–D). Notably, we identified a cluster expressing canonical parietal epithelial cell (PEC) marker CLDN1 but also co-expressing markers of rare PT-B/PT-C populations described by ref. ^56^ and PT-like Cluster 29 identified by ref. ^59^ (Fig. S3D). This mixed marker profile may be consistent with the transitional identity of cells at the PT/Bowman’s capsule interface^60–62^, and we therefore annotated this cluster as PEC/PT progenitor.

We then quantified the transcriptomic similarity between malignant cells in each tRCC tumor and each normal reference cell type. Across tRCC malignant cells, we found a substantial similarity to the vascular cell adhesion molecule-1 (*VCAM1*)-positive proximal tubule cells (PT_VCAM1) (Fig. S3E–H). PT_VCAM1 cells represent a subpopulation of proximal tubule cells that also show increased expression of kidney injury molecule-1 (KIM1, encoded by *HAVCR1*) and may represent cells with progenitor-like features that are expanded in the setting of renal injury^58,63,64^. Of note, plasma KIM1 has emerged as a biomarker of malignant pathology for kidney masses, recurrent RCC, and as a predictive marker of response to immunotherapy in metastatic RCC^65,66^. A subset of tRCC samples (e.g., TRCC6, TRCC11, TRCC19) showed highest similarity to the PEC/PT progenitor population; however, given that VCAM1 is expressed across PEC and other cell types throughout the kidney^58,67^, this likely reflects the shared VCAM1-expressing identity along the PT/Bowman’s capsule continuum rather than a strictly distinct cellular origin in these cases (Fig. S3E, G, H).

A parallel analysis on ccRCC tumors^38^ showed uniform mapping of ccRCC malignant cells to the same PT_VCAM1 subpopulation, consistent with a prior report implicating a *VCAM1*-expressing proximal tubule cell as the likely cell of origin for ccRCC^55^ (Fig. S3G). These findings were further supported by analysis of bulk RNA-seq datasets, which similarly suggested proximal tubule origin for both tRCC and ccRCC^54,56,57^ (Fig. S3H). Altogether, these findings suggest that tRCC and ccRCC most often arise from a shared *VCAM1*-expressing proximal tubular cell of origin, with a subset of tRCCs potentially arising from a closely related PEC/PT progenitor population, but undergo divergent transcriptional and epigenetic reprogramming in the context of distinct oncogenic drivers.

### Conserved oncogenic meta-programs in malignant tRCC cells

**Fig. 3:**
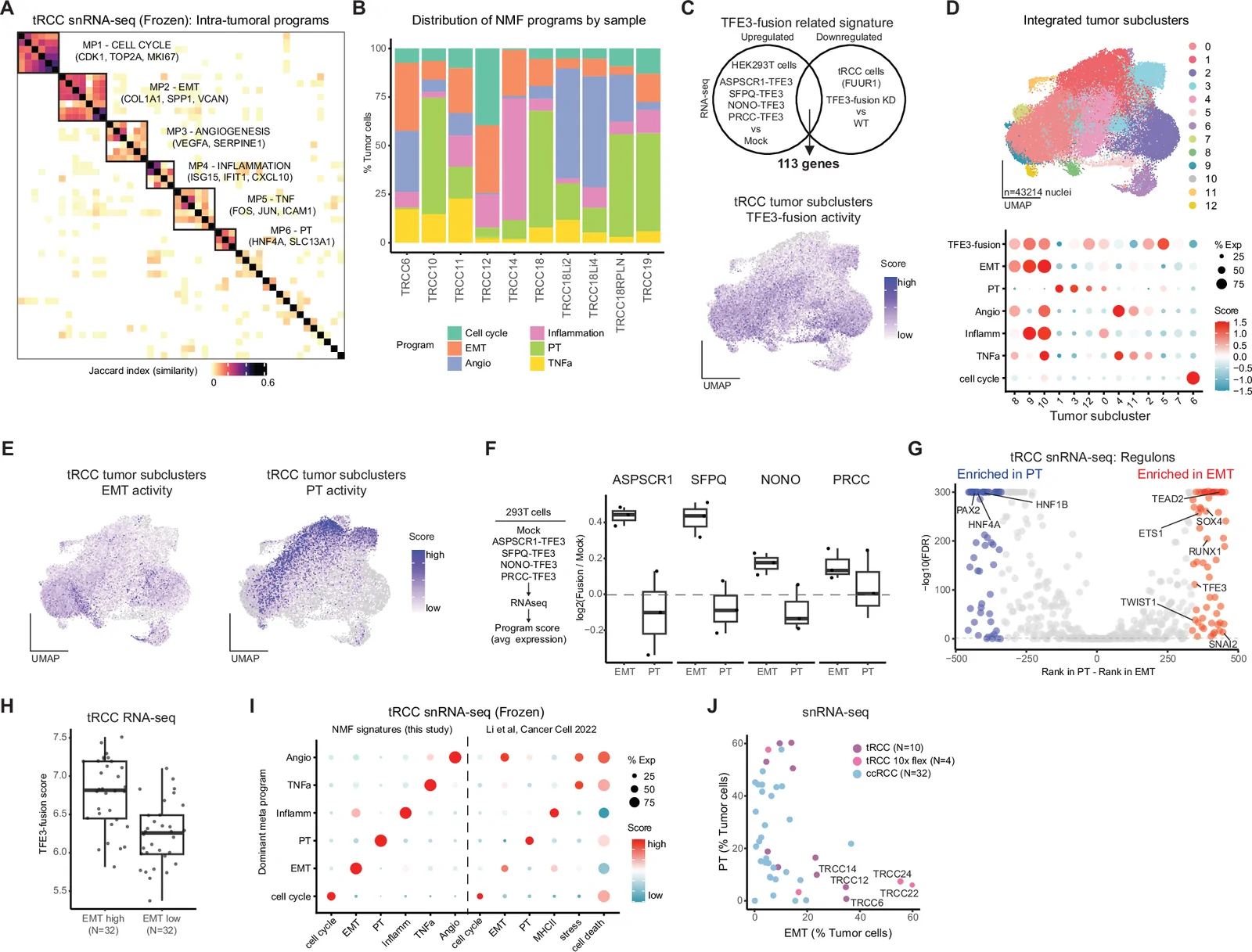
Consensus tumor meta-programs in tRCC. **A** Heatmap indicating similarity (Jaccard index) between individual tumor NMF programs in tRCC frozen cohort, with six conserved tRCC meta-programs (MP1-MP6) annotated. **B** Stacked bar plot showing the distribution of identified NMF programs across the tRCC snRNA-seq (frozen) cohort. **C** TFE3-regulated genes identified in in vitro gain- and loss-of-function experiments^12,30^ (top) were used to derive a TFE3 fusion activity signature. Bottom, UMAP representing TFE3-fusion activity score across tRCC tumor cells (frozen cohort). **D** UMAP representing subclusters from integrated tRCC tumor cells (top) and dot plot showing relative scores of tumor NMF programs as well as TFE3 fusion activity signature in each tumor subcluster in frozen cohort (bottom). **E** UMAPs representing EMT activity score (left) and PT activity score (right) in tRCC tumor cells (frozen cohort). **F** Box plots depicting changes in EMT and PT scores upon overexpression of various different TFE3-fusions in HEK-293T cells^12^ (*N* = 3 biological replicates). Graphs indicate medians with 25th and 75th percentiles, with points representing individual biological replicates. **G** Volcano plot representing transcription factor regulons identified in EMT vs PT cells from tRCC tumor snRNA-seq data. *X*-axis represents the difference in ranks of regulons in PT vs EMT cells. Regulons enriched in EMT are highlighted in red while those enriched in PT are highlighted in blue. **H** Boxplots showing TFE3-fusion activity score in EMT-high vs EMT-low tumors from bulk RNA-Seq data of tRCC (*N* = 32 tumor samples in each condition). Graphs indicate medians with 25th and 75th percentiles, with points representing individual biological replicates. **I** Dot plot showing association between tRCC NMF programs identified in this study (frozen cohort) and NMF programs defined previously in ccRCC^72^. **J** Scatterplot showing proportion of EMT and PT tumor cells in each tRCC and ccRCC sample.

We next sought to identify gene expression programs that were shared across multiple tumors in our cohort. We identified co-regulated gene modules via non-negative matrix factorization (NMF), converging upon six tumor meta-programs (MPs) that were each defined by the expression of canonical markers (Fig. 3A and Supplementary Data 3): (MP1) Cell cycle (*CDK1*, *TOP2A*, *MI67*); (MP2) EMT (*COL1A1*, *VCAN, FN1*); (MP3) Angiogenesis (*VEGFA*, *SERPINE1*); (MP4) Inflammation (*CXCL10*, *IFIT1*); (MP5) TNFα (*FOS*, *JUN*); (MP6) Proximal tubule (PT) (*HNF4A*, *SLC13A1*). These metaprograms were independently identified in both the frozen snRNA-seq and FFPE 10x Flex cohorts using the same analytical framework.

All six MPs were expressed to varying degrees in malignant cells in each tumor, indicative of substantial transcriptional heterogeneity, despite tRCC being a genetically quiet tumor with few recurrent genomic alterations apart from the driver fusion (Fig. S2A)^12,21–24,26^. The PT MP was the most prevalent across all malignant cells (23.8% of all malignant cells overall; range 0.7–60.2% per sample), reinforcing a proximal tubular epithelial cell origin for tRCC. The cell cycle MP, likely reflective of rapidly cycling cells, was mutually exclusive with all other MPs; a recent scRNA-seq study has suggested enrichment of a similar program amongst tumor progenitor cells in synovial sarcoma^68^ (Figs. 3B–D and S4A).

Intriguingly, ~20% of malignant cells (1–57%) in tRCC expressed an angiogenesis MP, despite the fact that tRCCs do not harbor *VHL* loss and therefore do not have genetically enforced activation of angiogenesis via HIF-2a signaling, as in ccRCC^3–6^. Nonetheless, the expression of an angiogenesis MP in a subset of malignant tRCC cells is consistent with the clinical observation of improved outcomes with VEGF targeted therapy or immunotherapy-VEGF combinations (IO/TKI) rather than immunotherapy alone in tRCC^17,69^. Notably, the angiogenesis MP was frequently co-expressed with either the inflammation or TNFa MPs, or both, perhaps suggesting activation of angiogenesis by inflammatory cytokines in tRCC^70,71^ (Fig. 3D).

The EMT and PT MPs were mutually exclusive in malignant cells, consistent with a recent scRNA-seq study of ccRCC^72^. This held across patients, with malignant cells across the entire cohort integrated into 12 subclusters (Fig. 3D, E). Moreover, snRNA-seq of the primary tumor and three metastatic sites from a recently reported tRCC rapid autopsy case^21^ revealed that the EMT MP was stronger in the metastatic sites relative to the primary tumor, while the PT MP showed the inverse pattern (Fig. S4B–D). Whether this reflects selective outgrowth of EMT-high cells during metastatic dissemination, induction of EMT by the metastatic microenvironment leading to loss of proximal tubule identity, or both, is unclear. Nevertheless, this is consistent with multiple prior functional studies in which EMT confers migratory and invasive capacity during metastatic dissemination^73–75^.

We next sought to determine to what extent these MPs were directly linked to TFE3 fusion activity. We derived a consensus signature of 113 fusion-regulated genes based on RNA-seq data from two in vitro perturbation experiments: overexpression of TFE3 fusion constructs in HEK293T cells^12^ and knockdown of the endogenous *TFE3* fusion in tRCC cell line, FUUR1 (Fig. 3C and Supplementary Data 3)^30^. Malignant cells displayed substantial heterogeneity in the fusion transcriptional signature score, with a higher signature score associated with increased utilization of the EMT MP and inversely associated with utilization of the PT MP (Fig. 3C–E). This relationship was recapitulated in vitro by overexpression of four different *TFE3* fusions in HEK293T embryonic kidney cells^12^ (Fig. 3F).

To further investigate the relationship between TFE3 fusion activity and EMT program, we performed gene set enrichment analysis following TFE3 fusion knockdown in three tRCC cell lines (FUUR1 [ASPSCR1-TFE3]: NES = −1.86, *P* = 0.0015; UOK120 [PRCC-TFE3]: NES = −2.17, *P* = 0.0023; UOK124 [PRCC-TFE3]: NES = −2.85, *P* = 0.0017). Knockdown of the TFE3 fusion enriched the Hallmark EMT gene signature in the cell line context but also impaired cell migration of FUUR1 cells in a wound healing assay (Fig. S5A, B). RNA-Seq profiles from two different published murine tumor models driven by ASPSCR1-TFE3^76^ and SFPQ-TFE3^77^ fusions showed significant enrichment of the Hallmark EMT signature in tumor compared to normal kidney tissue (NES = 2.4, *P* = 0.0001 and NES = 1.49, *P* = 0.0013, respectively). Together, these findings indicate a link between TFE3 fusion activity and changes in EMT in vitro and in vivo (Fig. S5C).

In further support of TFE3 activity skewing towards an EMT cell state, regulon analysis revealed that EMT-high malignant cells were enriched for motifs associated with TFE3 along with canonical EMT-associated transcription factors including TWIST1, SOX4, RUNX1, SNAI2, ETS1, and TEAD2^51,78–82^; conversely, lineage factors associated with renal epithelial cell identity (e.g., PAX2, HNF4A, and HNF1B^83–85^) were enriched in PT-high cells (Fig. 3G). We also analyzed bulk RNA-Seq data from 128 tRCC tumors across 5 published cohorts^22,23,35–37^ and confirmed that a higher TFE3 fusion signature score was positively correlated with an EMT signature score and inversely correlated with a PT signature score (Figs. 3H and S4E).

Altogether, our observation of a PT MP conserved across all samples supports a proximal tubule cell of origin for tRCC, while its mutually exclusive expression with an EMT program is suggestive of a fusion-driven mesenchymal differentiation program leading to loss of epithelial cellular identity.

### Comparative analysis of meta-programs in ccRCC

As tRCC is a rare and biologically unique tumor type not well-represented in recent genomic atlas efforts, we next sought to contextualize our conserved tRCC MPs with respect to those identified in other cancers. We first compared our tRCC oncogenic MPs with those recently defined in a cohort of 10 ccRCC samples profiled by scRNA-seq^72^. All six tRCC MPs overlapped substantially with one or more gene programs identified in ccRCC: EMT, cell cycle, and PT programs were identified in both studies while our tRCC MPs of inflammation and TNFa mapped remarkably well to MHCII and stress/cell death programs identified in ccRCC, respectively; the tRCC angiogenesis MP mapped variably to the EMT, stress, and cell death MPs in ccRCC (Fig. 3I). Despite several malignant cell programs being shared between tRCC and ccRCC, there were stark differences in their utilization: most notably, tRCC samples more commonly showed substantial EMT MP utilization relative to ccRCC (6/14; 43% for tRCC vs. 1/32; 3% samples for ccRCC at threshold of >20% tumor cells) (Figs. 3J and S4F).

Our tRCC MPs also mapped to states identified in a recent comprehensive pan-cancer single cell study of >1100 tumors spanning 24 tumor types^45^ (Fig. S4G). The tRCC EMT MP mapped strongly to several EMT states identified in this pan-cancer study, as well as to a mesenchymal program (MES) identified in glioma. Interestingly, the tRCC EMT MP also overlapped with a skin pigmentation program associated with MITF activity in melanoma—consistent with our results above that implicate the TFE3 fusion (an MITF homolog) in directly driving EMT. Our tRCC PT MP exhibited a strong correlation with a pan-cancer glutathione program enriched in ccRCC, and consistent with a critical role for glutathione handling in renal proximal tubule cells^86^. The tRCC angiogenesis MP matched to the pan-cancer hypoxia program but also had partial alignment with many additional programs; together with its variable matching against ccRCC MPs, as discussed above, this suggests that this particular MP may be somewhat unique to tRCC, although we cannot exclude signature-related differences due to sample preparation or platform.

Overall, our results suggest that, despite harboring a dominant genetic driver distinct from almost all other cancers, tRCC cells converge on broadly conserved core oncogenic programs observed in other solid tumors.

### Dysfunctional T cell phenotypes and response to ICI in tRCC

Responses to immunotherapy vary widely across kidney cancer subtypes: while ccRCC is generally considered an immune-responsive tumor, response to immunotherapy in other RCCs is more variable, with low immune-responsiveness in certain subtypes such as renal medullary carcinoma^87^ and chRCC^88^. Several small studies suggest some tRCCs do respond to immunotherapy, albeit at a lower rate than ccRCC^12,17,22,89^. Variable immune-responsiveness across RCC subtypes likely stems both from differences in the quantity and nature of tumor antigens capable of stimulating an anti-tumor immune response, and in microenvironmental factors that may constrain anti-tumor immunity.

**Fig. 4:**
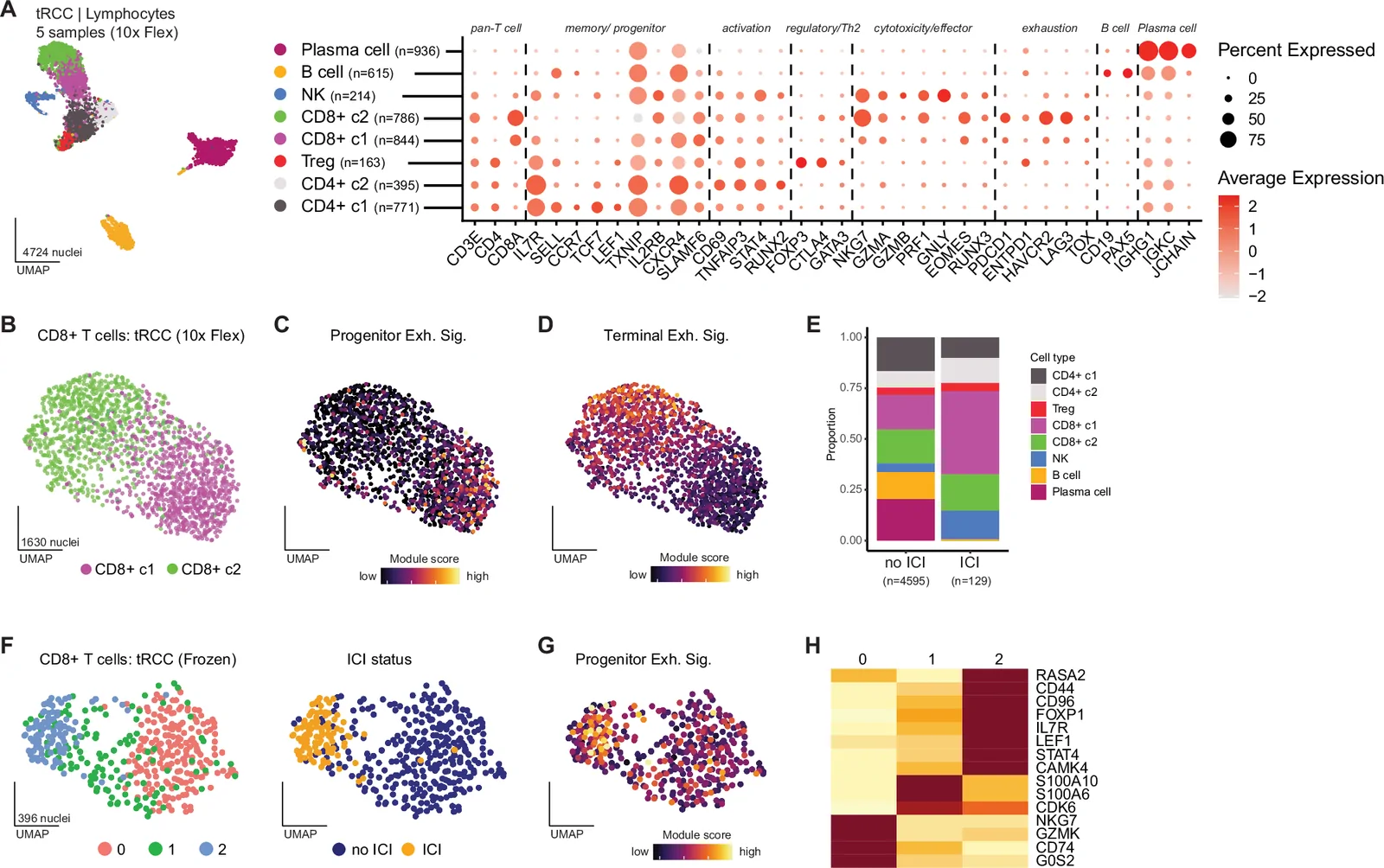
CD8 T cell states in the tRCC TME. **A** UMAP representation of all lymphocytes (left) as well as annotation and marker genes of lymphocyte subsets (right) in the tRCC (10x Flex) cohort. **B** UMAP of CD8+ T cell clusters (10x Flex cohort). **C** UMAP representing progenitor exhausted signature score across CD8+ T cells (10x Flex cohort). **D** UMAP representing terminally exhausted signature score across CD8+ T cells (10x Flex cohort). **E** Stacked bar plot depicting proportion of lymphocyte subtypes in tRCC samples from ICI-naïve (left) and ICI-treated (right) patients. **F** UMAP of CD8+ T cell clusters (left) colored by ICI status (right) in the tRCC frozen cohort. **G** UMAP representing progenitor exhausted signature score across CD8 T cells from the tRCC frozen cohort. **H** Heatmap with top expressed marker genes associated with CD8+ T cell subclusters from the tRCC frozen cohort.

We therefore sought to define the tumor microenvironment (TME) composition in tRCC, particularly in light of our initial observation that tRCCs diverge from ccRCCs on the basis of immune/stromal gene expression programs (Fig. 1E). Focusing initially on lymphocytes, cell type identification revealed CD4+ T cells, CD8+ T cells, regulatory T cells (Tregs), natural killer (NK) cells, B cells, and plasma cells in tRCC tumor samples (Fig. 4A; 10x-Flex fixed RNA-Seq cohort was used for this analysis due to greater representation of lymphocytes, Fig. S1F).

Notably, despite an overall decreased T lymphocyte abundance compared with ccRCC (Fig. 1E and ref. ^12^), both CD4+ and CD8+ T cells were still present in the tRCC TME (Fig. S6A). This finding is a notable contrast from chRCC, which is a typically “cold” tumor with an exceedingly low proportion of infiltrating immune cells^88,90^. Among CD4+ T cells, we observed two clusters: CD4 c1, with expression of *IL7R*, *TCF7*, and *CCR7* consistent with a naïve/central memory phenotype; and CD4 c2, which exhibits features of activated/effector memory phenotype with expression of *STAT4*, *RUNX2*, *CD69*, and *TNFAIP3*^91,92^. Together with the presence of abundant plasma cells (Fig. 4A), the presence of central memory-type CD4 cells may be suggestive of the presence of an immature tertiary lymphoid structures—ectopic lymphoid formations resembling secondary lymphoid organs—which, in their mature form, have recently been linked to immune-responsiveness in ccRCC^93,94^. Additionally, although tRCC tumors displayed sparse NK cell infiltrate, these NK cells skewed toward cytotoxicity rather than a dysfunctional tumor-resident phenotype that has been recently linked to poor outcomes in ccRCC^95^ (Fig. S6B).

Given our finding that tRCCs do indeed possess CD8+ tumor-infiltrating lymphocytes (TILs), we next turned our attention to defining CD8+ T cell states that might modulate immune responsiveness. Prior studies have elucidated heterogeneity in exhausted CD8^+^ TIL populations: progenitor-exhausted CD8+ TILs exhibit polyfunctionality, persistence, share properties with exhausted CD8+ T cells in chronic viral infection models and are capable of being reinvigorated by ICI, while terminally-exhausted CD8+ T cells display greater cytotoxicity but have reduced long-term survival^96^.

Reclustering of CD8+ cells in our 10x flex cohort revealed two subclusters (Fig. 4B): CD8 c1 retained expression of stem-associated and memory markers including *IL7R*, *TCF7*, *CXCR4*, and *SLAMF6*, and exhibited intermediate *PDCD1* and *TOX* levels, consistent with a progenitor-like or early exhausted phenotype. In contrast, CD8 c2 demonstrated higher expression of canonical terminal exhaustion markers including *PDCD1*, *LAG3*, *ENTPD1*, and *HAVCR2*^91,96,97^ (Fig. 4C, D). While limited by the inclusion of only one ICI-treated sample in the FFPE cohort, a higher proportion of progenitor-exhausted CD8+ T cells (CD8 c1) was observed in the ICI-exposed sample compared to ICI-naïve tumors (Fig. 4E).

These results were confirmed in the independent frozen snRNA-seq cohort, where clustering of CD8+ T cells revealed three clusters, with clusters 0 and 1 being primarily derived from ICI-naive tumors and cluster 2 enriched in ICI-treated samples and displaying a higher progenitor-exhausted signature, similar to CD8 c1 in the FFPE cohort (Fig. 4F, G). Functional annotation of these clusters via marker gene expression and gene signature scoring revealed a progression from naïve/quiescent (cluster 0; *G0S2*, *CD74*^98,99^) to proliferative/activated (cluster 1; *CDK6*, *S100A6*, *S100A10*), and ultimately to a progenitor-exhausted/memory-like phenotype (cluster 2; *IL7R*, *FOXP1*, *CD44*, *CD96*, *LEF1*, and *RASA2*^100–105^) (Fig. 4G, H). Metabolic pathway module scores were also consistent with a naïve-like phenotype in cluster 0 (high oxidative phosphorylation^106^), a proliferative phenotype in cluster 1 (high glycolysis/mTOR signaling^107^), and a memory-like phenotype in cluster 2 (high fatty acid oxidation^106^) (Fig. S6C).

Overall, our findings suggest that at least some tRCC cases harbor a progenitor-exhausted like CD8+ T cell reservoir, the population thought to be targeted by ICI for anti-tumor CD8+ T cell activity^96^.

Although tRCC has a very low mutational burden^12^, the driver fusion may represent a strong and clonal neoantigen, given that the *TFE3* fusion junction spans two genes that are not normally juxtaposed^26^. To evaluate for clonal expansion of tumor-responsive T cells, we performed TCR sequencing on 11 tRCC tumor samples (of which 8 tumors were in our single-cell cohort). After excluding publicly reported human viral antigen TCR sequences, we observed a median of 485 TCR-α and 890 TCR-β non-viral unique clonotypes per sample (range 345–2535 TCR-α & 519–4208 TCR-β), which were classified as hyperexpanded (>3%), large (>1%), medium (>0.01%), and small (<0.01%) groups based on their frequency in each sample (Fig. S6D–F). Sample TRCC14 was an outlier in terms of number of unique clonotypes; this sample also had the highest lymphocyte infiltration and interferon module score on snRNA-seq profiling (Fig. S6E). However, all samples had large or hyperexpanded TCR clones to varying degrees, suggesting the presence of expanded TIL populations responding to tumor-specific antigens. Nonetheless, due to the range of potential *TFE3* fusion partners and breakpoints^21^ coupled with HLA-type diversity, the strength and quality of a fusion-derived neoantigen may vary considerably between patients.

Overall, our analyses point to progenitor-exhausted CD8+ T cells and clonally-expanded TILs in tRCC, including after ICI exposure, suggestive of capacity for an effective anti-tumor T cell response. However, despite enrichment of progenitor-exhausted CD8+ T cells following ICI exposure, terminally exhausted CD8+ TILs were not proportionally expanded (Fig. 4E). This implies an immunologically active yet functionally restrained TME in tRCC, which may explain the limited clinical efficacy of single agent PD1/PD-L1 inhibitors in tRCC.

### Suppressive macrophages and fibroblasts in the tRCC TME

**Fig. 5:**
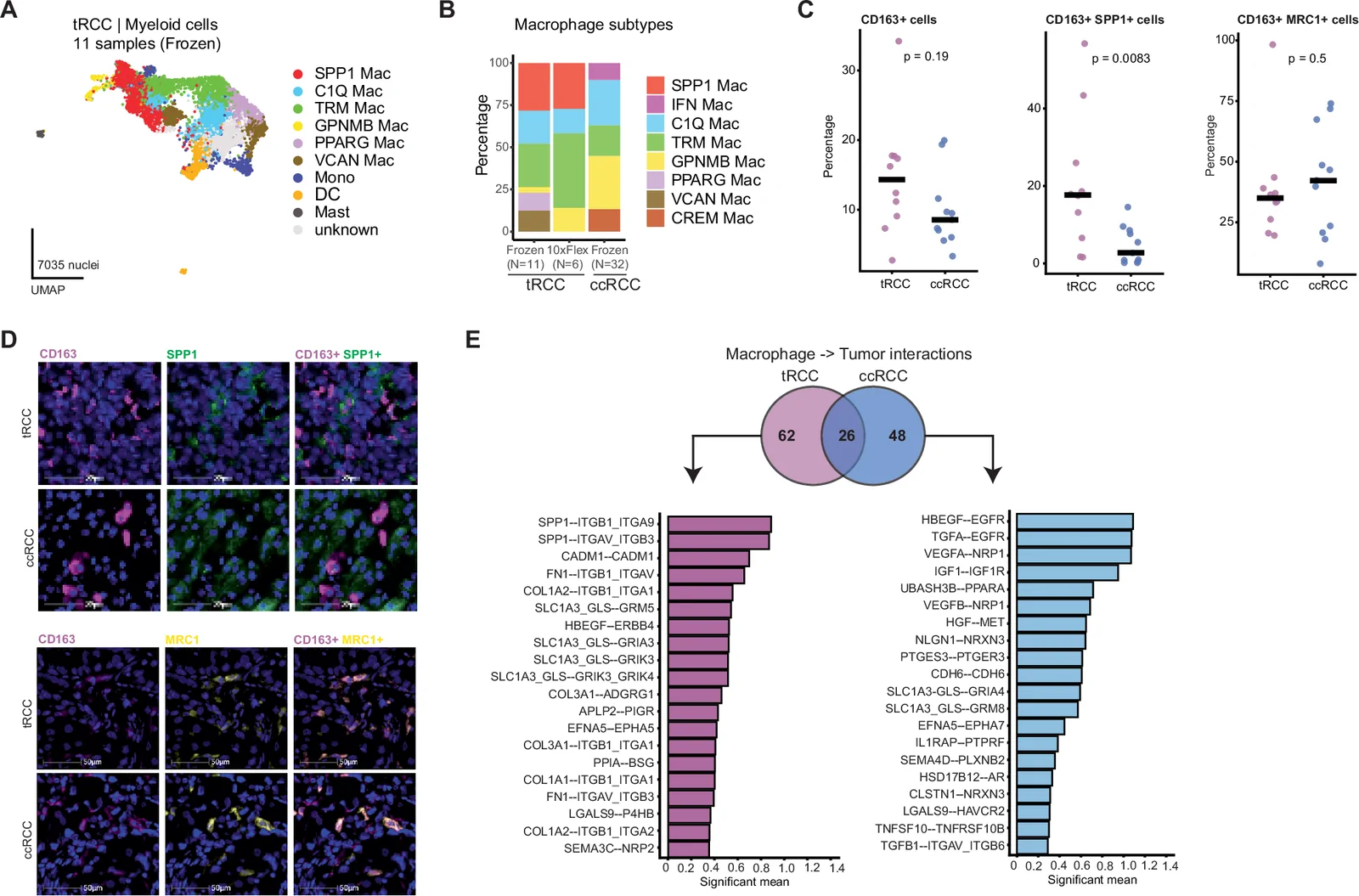
Macrophages in tRCC and ccRCC. **A** UMAP depicting myeloid cell subtypes identified in the tRCC tumors from the frozen cohort. **B** Stacked bar plot of macrophage subtypes in tRCC vs. ccRCC. **C** Quantification of percentage of CD163+ cells (left), CD163 + SPP1+ cells (middle), and CD163 + MRC1+ cells (right) in tRCC (*N* = 10) and ccRCC (*N* = 11) cases via multiple immunofluorescence. *P*-values are calculated by two-sided Wilcoxon’s rank-sum test. **D** Multiplex immunofluorescence for CD163, SPP1, and MRC1 in representative tRCC and ccRCC cases. Red arrows indicate SPP1 + CD163+ cells, and green arrows indicate MRC1 + CD163+ cells. **E** Cell–cell interactions from macrophages to tumor cells in tRCC vs ccRCC, highlighting top unique interactions in bar plots.

We next sought to determine if the constitution of the myeloid compartment in tRCC could reconcile the presence of tumor-specific TILs capable of responding to ICI with the typically modest responses to ICI clinically observed in tRCC^17,108^. By proportion, myeloid cells constituted the largest compartment of the tRCC TME (Fig. S1F), which included a number of different macrophage subtypes, including: SPP1+ macrophages, C1Q+ macrophages, tissue resident macrophages, and GPNMB macrophages (Figs. 5A and S7A, B). Comparison of macrophage subtypes in a published ccRCC snRNA-seq dataset^38^ revealed stark differences with tRCC. SPP1+ macrophages, recently implicated in the suppression of CD8 + TIL activity and adverse clinical outcomes across several tumor types^109–111^, comprised approximately 25% of macrophages in tRCC but were virtually absent in the ccRCC cohort. By contrast, IFN-primed macrophages, which are associated with anti-tumor effects^112,113^, constituted ~10% of the myeloid population in ccRCC but were undetectable in tRCC (Figs. 5B and S7C). Multiplex immunofluorescence across 10 tRCC and 11 ccRCC cases further confirmed a significant enrichment of CD163 + SPP1+ macrophages in tRCC relative to ccRCC, while the proportion of CD163 + MRC1+ macrophages did not differ between the two tumor subtypes (Fig. 5C, D).

Recent scRNA-seq studies have facilitated the identification of myriad cancer-associated fibroblast (CAF) subpopulations, which differ in transcriptional state and function^114,115^. We observed that tRCCs exhibited multiple fibroblast types including vascular CAFs, matrix CAFs (mCAFs), and myofibroblasts (Fig. S7D). Notably, the two latter fibroblast types have also both been linked to a suppressive TME: mCAFs have been linked to the recruitment and differentiation of SPP1+ macrophages^116^ while myofibroblasts have been linked both to M2-polarization of macrophages and promotion of a desmoplastic stroma^117–119^.

Together, these findings point to a suppressive TME in tRCC, which may constrain response to immunotherapy despite adequate tumor-associated antigens, in part through the accumulation of SPP1+ macrophages that contribute to T cell dysfunction via impaired antigen presentation, and secretion of osteopontin that reinforces a non-permissive stromal rich immune niche^111,120^.

### A crosstalk of suppressive signals constrains immune-responsiveness in tRCC

To better define the crosstalk between malignant, immune, and stromal compartments in tRCC, we performed ligand-receptor interaction analyses across our tRCC snRNA-seq cohorts and compared them to ccRCC. Consistent with the preferential accumulation of SPP1+ macrophages observed above, macrophage-to-tumor cell interactions were significantly enriched in tRCC relative to ccRCC (Figs. 5E and S7E). Of the tRCC-specific macrophage-to-tumor interactions, SPP1-integrin axes (SPP1 → ITGB1/ITGA9, SPP1 → ITGAV/ITGB3) and ECM-integrin signaling (COL1A1/COL1A2/FN1 → ITGB1/ITGAV) predominated, reflecting the immunosuppressive and pro-stromal identity of SPP1+ macrophages as the dominant myeloid population in tRCC. By contrast, ccRCC-specific interactions were enriched for growth factor receptor signaling—including HBEGF → EGFR, TGFA → EGFR, VEGFA/B → NRP1, and IGF1 → IGF1R—consistent with HIF-driven angiogenic and proliferative signaling in that disease. Together, these findings reveal that macrophages engage tumor cells through fundamentally distinct axes in tRCC versus ccRCC, shaped by divergent macrophage composition and oncogenic context.

**Fig. 6:**
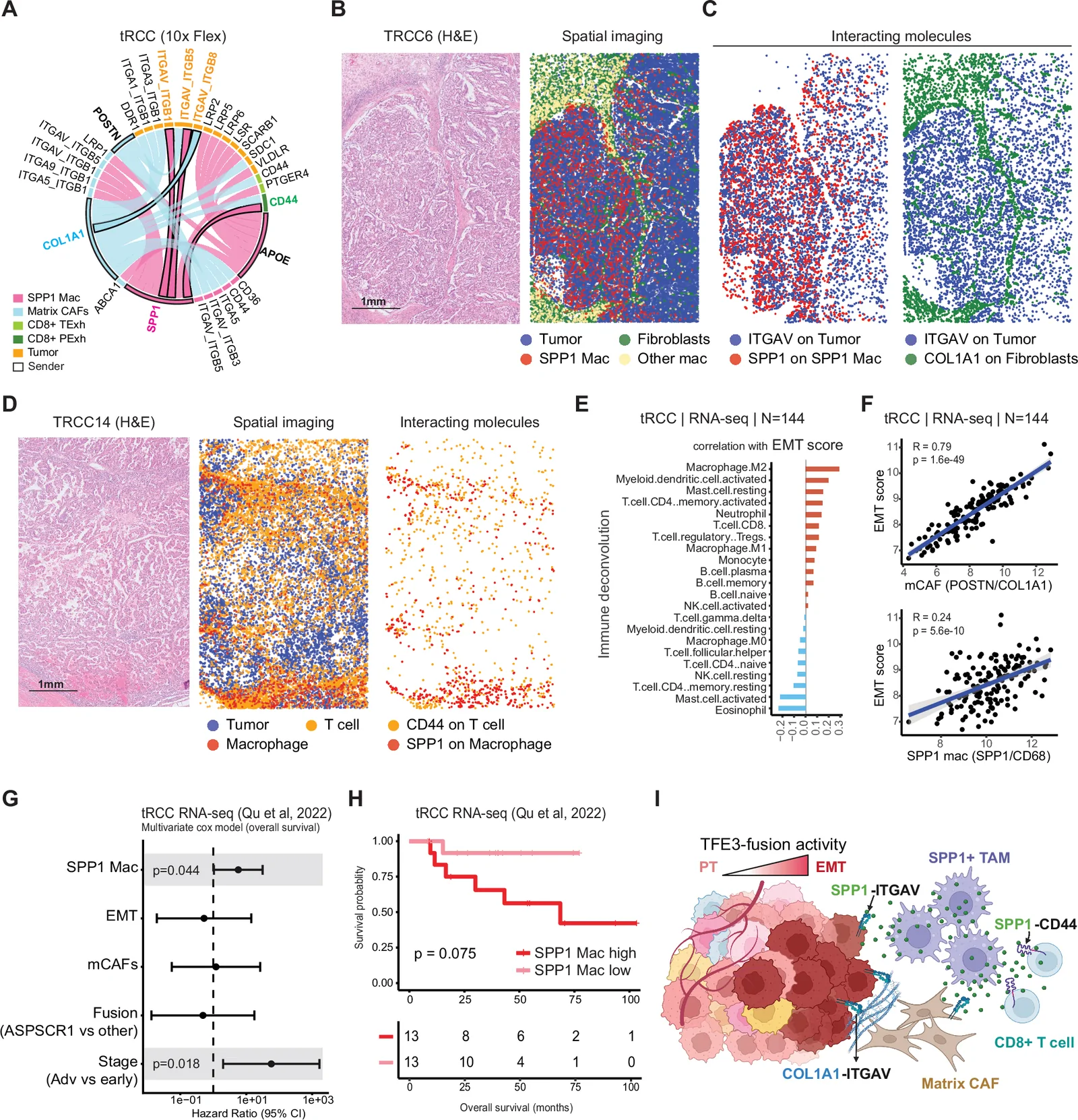
A suppressive tumor microenvironment in tRCC. **A** Chord diagram showing inferred interactions of SPP1 macrophages with Matrix CAFs, tumor cells and CD8 T cell subtypes in the tRCC (10x flex) cohort. **B** Hematoxylin and eosin (H&E) staining (left) and spatial transcriptomics (Nanostring CosMX, right) of a section of tRCC tumor from sample TRCC6. Tumor cells, fibroblasts, SPP1+ macrophages and other macrophages are highlighted in the spatial image plot. **C** Spatial plots highlighting distribution of interacting molecules: SPP1 on SPP1+ macrophages with ITGAV on tumor cells (left) and COL1A1 on fibroblasts with ITGAV on tumor cells (right). **D** H&E staining (left) and spatial imaging (Nanostring CosMX, middle) of a section of tRCC tumor from sample TRCC14. Tumor cells, T cells and macrophages are highlighted in the spatial image plot. Spatial plot highlighting distribution of interacting molecules: SPP1 on macrophages and CD44 on T cells (right). **E** Correlation between sample-wise immune cell deconvolution scores from tRCC bulk RNA-seq data with corresponding EMT scores. **F** Scatterplots showing correlation of EMT score with Matrix CAFs (top) and SPP1 Macrophages (bottom) in tRCC bulk RNA-seq cohort (*N* = 144). Lines indicate linear regression fits and grey shaded bands represent the 95% confidence intervals. Pearson correlation coefficients and two-sided *P* values are shown. **G** Multivariate cox model on overall survival from tRCC samples (*N* = 26) and association with SPP1 macrophages, EMT, matrix CAFs, TFE3 fusion partner (ASPSCR1 vs other) and tumor stage (advanced vs early) as predictors. Dots represent estimated hazard ratios and error bars represent corresponding 95% confidence intervals. Significant predictors, assessed via two-sided wald tests from multivariable coz proportional hazards model are highlighted. **H** Kaplan–Meier survival curve for SPP1 low and SPP1 high tRCC samples in Qu et al. dataset. *P*-value is calculated by a pairwise log-rank test. **I** Schematic illustrating suppressive tumor microenvironment (TME) in tRCC. Tumor cells exist in distinct transcriptional states, with TFE3-fusion driving repression of the epithelial identity and promoting transition to a mesenchymal state. Key suppressive interactions highlighted in this study are highlighted, including SPP1–ITGAV signaling from macrophages to tumor cells, SPP1–CD44 signaling from macrophages to CD8+ T cells, and COL1A1–ITGAV signaling from matrix CAFs to tumor cells.

Within tRCC, SPP1+ macrophages and mCAFs emerged as central hubs of a dense suppressive network (Figs. 6A and S7F, G; “Methods”). Prominent candidate interactions included SPP1 on macrophages to ITGAV on tumor cells, COL1A1 on mCAFs to ITGAV on tumor cells, POSTN on mCAFs to ITGB3 on macrophages, and SPP1 on macrophages to CD44 on progenitor-exhausted CD8+ TILs. All of these putative interactions have been linked to the suppression of anti-tumor T-cell responses or to limiting the efficacy of immune therapies^120–130^ (Figs. 6A and S7F, G).

To assess how these interactions were spatially organized within the tumor architecture, we performed single-cell spatial transcriptomics using the Nanostring CosMx platform on two tRCC specimens (TRCC6 and TRCC14, Fig. S7H–J). Spatial ligand-receptor mapping confirmed the presence of SPP1-ITGAV and COL1A1-ITGAV interactions at points of macrophage-tumor and fibroblast-tumor interaction, respectively, in the TRCC6 sample (Fig. 6B, C). Quantitative spatial proximity analysis demonstrated that 51.5% of SPP1+ macrophages were within 20 µm of an ITGAV+ tumor cell, and 15.0% of COL1A1+ fibroblasts were within 20 µm of an ITGAV+ tumor cell, confirming the spatial co-localization of ligand- and receptor-expressing cells at the tumor interface. We also observed that EMT-high tumor cells were in proximity to SPP1+ macrophages in this sample (Fig. S7I). In TRCC14 (the sample with highest T-cell infiltration on snRNA-seq, Fig. S6E), we additionally identified spatial proximity between SPP1+ macrophages and CD44+ CD8+ T cells, a known suppressive axis implicated in T cell exhaustion^128^, with 4.1% of SPP1+ macrophages residing within 20 µm of a CD44+ T cell (Fig. 6D). Moreover, these associations were validated in a bulk RNA-seq dataset of 95 tRCC tumors, where an EMT signature score positively correlated with signatures for M2-like macrophages (by immune deconvolution), mCAFs, and SPP1+ macrophages (Fig. 6E, F).

Finally, to evaluate the clinical consequences of our findings, we determined the association between overall survival and RNA signatures of EMT, SPP1+ macrophages (SPP1/CD68) and mCAFs (COL1A1/POSTN) in a tRCC bulk RNA-seq cohort^23^. Via Cox multivariable regression incorporating tumor stage and fusion partner type (ASPSCR1-TFE3 vs other), SPP1+ macrophage score and advanced stage emerged as independent predictors of decreased survival in tRCC (Fig. 6G, H).

Overall, these results support the notion that an EMT program in tRCC malignant cells contributes to an immunosuppressive microenvironment through spatially structured interactions between EMT-activated tumor cells, SPP1+ macrophages, mCAFs, and CD8+ TILs (Fig. 6I).

## Discussion

By integrating snRNA-seq with snATAC-seq, spatial transcriptomics, and TCR-seq on a cohort of 20 tRCC samples from 15 patients, we present a detailed molecular view of tRCC. Our study identifies conserved malignant transcriptional programs in tRCC, clarifies the cellular origin of this cancer, and elucidates how activity of the TFE3 driver fusion remodels the immune microenvironment. Additionally, we validate our key results against 128 tRCC bulk transcriptomes and perform a detailed comparative analysis with ccRCC (the most well-studied kidney cancer), revealing significant molecular differences. To our knowledge, this represents the most exhaustive single-cell study performed to date on this rare cancer and offers a framework for mechanism-inspired target discovery in this aggressive RCC subtype.

We observed six conserved MPs in tRCC, which mostly overlap with oncogenic MPs identified in ccRCC or other cancers, with some key distinctions. First, while PT and EMT MPs are identified in both tRCC and ccRCC, their utilization differs substantially; the EMT MP is more prevalent in tRCC malignant cells, whereas the PT program is more dominant in ccRCC. The induction of a mesenchymal phenotype in tRCC may be linked to TFE3-fusion activity (Fig. 3C, D)^23,31,131^. While convergent evidence supports a functional role for the fusion regulating EMT, the mechanism may be indirect—with the fusion establishing transcriptional permissiveness for EMT that requires cooperative microenvironmental signals (e.g., from SPP1+ macrophages and mCAFs) for full effect, and the effects may be context-specific^132^. Additionally, some tRCC malignant cells express an angiogenesis MP, notably in the absence of *VHL* inactivation-associated HIF2a signaling observed in ccRCC^3–6^. This suggests alternative mechanisms of neovascularization in tRCC, possibly mediated by inflammatory cytokines, as evidenced by co-enrichment of TNF and inflammatory MPs with the angiogenesis MP^70,71^ (Fig. 3D). This observation may also explain the higher response rates to immune checkpoint inhibitor (ICI)-VEGF inhibitor combination therapies compared to ICI alone^17^.

Despite the genomic homogeneity of tRCC, which is characterized by a low mutational burden and relatively few recurrent alterations beyond the driver fusion^12,21–23^, we observed significant transcriptional heterogeneity both within and among tumors. Similar non-genetic heterogeneity has been described in other fusion-driven cancers, including Ewing sarcoma^133,134^, alveolar rhabdomyosarcoma^135,136^, and synovial sarcoma^68^, where variation in fusion oncogene activity gives rise to distinct cellular states associated with metabolism, differentiation, or immune evasion. This transcriptional plasticity has also been identified for c-Myc—which, like TFE3, is an oncogenic transcription factor in the bHLH-LZ family^137^. In tRCC, cell-to-cell variability in TFE3 fusion activity may stem from heterogeneity in the expression levels of the fusion itself, variability in the expression of key co-factors or differences in chromatin accessibility.

Our single-cell analyses indicate that despite different genetic drivers and transcriptional programs, tRCC and ccRCC both originate from VCAM1-expressing proximal tubule cells. Interestingly, other origins for tRCC, such as PECs, were predicted in a few cases. While these instances could represent imprecise cell type classification, an alternative explanation is that tRCC has the capacity to arise from multiple potential cell types that are permissive to generate the driver fusion. The presence of *TFE3* fusions in non-kidney cancers (e.g., ASPS, PEComa) further supports their potential to emerge from diverse cells^37,138–142^. Additionally, it is plausible that the histological plasticity observed in tRCC—which can microscopically resemble both clear-cell and papillary subtype RCCs—may be rooted in its ability to arise in diverse cells of origin.

Our study provides mechanistic insight into the clinical observation of suboptimal responses to ICI in tRCC^12,17,89,143^. The TME in tRCC contains immunosuppressive cells like SPP1+ TAMs and COL1A1+ mCAFs, which collaborate to suppress the anti-tumor activity of CD8+ TILs^114,115^. In addition to a suppressive SPP1+ TAM/CD8+ T-cell interaction^128^, SPP1 secreted by TAMs activates fibroblasts, amplifying matrix deposition^120,129,130,144–147^ and further immune suppression^109–111,148^. While SPP1+ macrophages have been reported in ccRCC^149^ and implicated in CD8+ T cell dysfunction in that context, their substantially greater abundance in tRCC (Fig. 5B–D) likely amplifies this suppressive axis and may contribute to the more limited immunotherapy responsiveness observed in tRCC relative to ccRCC. The near-complete absence of SPP1+ macrophages in our ccRCC comparator dataset^38^ (Fig. 5B) may also in part reflect differences in patient population and sample processing between the two studies. Notably, these suppressive populations have been linked to a tumor-intrinsic EMT program in other cancers^150–155^, which we show is also activated in tRCC downstream of the driver fusion. Thus, despite potential neoantigens derived from the TFE3 fusions, a suppressive TME may constrain anti-tumor immunity in tRCC.

Interestingly, ASPS, which also harbors a TFE3 driver fusion, was shown to have a 37% objective response rate to the single-agent PD-L1 inhibitor, atezolizumab, in a recent clinical study^156^. Whether TFE3 fusion partner identity contributes to differences in ICI responsiveness through distinct TME composition, or whether this reflects intrinsic biological differences between tRCC and ASPS, remains an important open question. Bulk transcriptomic data^22^ suggest that *ASPSCR1-TFE3* fusions may be associated with enrichment of stromal and angiogenic signatures, raising the possibility that fusion-partner-specific transcriptional programs shape the immune microenvironment in ways that influence therapeutic response. A comparative analysis of tRCC across fusion subtypes, alongside ASPS, may offer an opportunity to disambiguate microenvironmental factors from oncogenic drivers alone in determining immune resistance.

More broadly, targeting the fusion-driven EMT-macrophage-fibroblast circuit may represent a strategy to reprogram the TME and potentiate immune therapies in tRCC.

## Methods

### Study approval and sample collection

All research described in this study complied with all relevant ethical regulations. The collection and research use of human tumor specimens were approved by the Dana-Farber/Harvard Cancer Center Institutional Review Board. All participants provided written informed consent for the collection, sequencing and research use of their samples. No patient compensation was provided. Sex was reported based on patient self-report. Sex or Gender were not prospectively considered in the study design.

A total of 20 tRCC tumors from 15 individuals were collected retrospectively from the Arthur and Linda Gelb Center for Translational Research at Dana-Farber Cancer Institute and sequenced using one or more of the following platforms: 10x Genomics 3′ snRNA-seq, 10x 3′ multiome (RNA + ATAC), 10x-Flex fixed RNA-seq, bulk TCR sequencing, and spatial transcriptomics. For subject TRCC18, the primary tumor was collected during initial nephrectomy, and 13 metastatic sites were collected during rapid autopsy as previously described, 3 of which were subjected to snRNA-seq^21^ (see Fig. S3). The cohort included 8 females and 7 males. Sample preservation methods (fresh frozen or FFPE-fixed) and additional clinical details are provided in Supplementary Data 1 and Fig. S1A. All individuals provided written informed consent for the use of their samples in sequencing and research. The rapid autopsy for subject TRCC18 was performed under a genitourinary oncology rapid autopsy protocol approved by the Dana-Farber/Harvard Cancer Center Institutional Review Board (IRB).

### Cell culture

The human tRCC cell line FUUR1, obtained from the laboratory of M. Ishiguro at Fukuoka University School of Medicine, was cultured at 37 °C in a humidified incubator with 5% CO₂ in Dulbecco’s modified Eagle medium supplemented with 10% fetal bovine serum and 100 U ml⁻¹ penicillin.

### Single-nucleus RNA sequencing (3′ RNA and 3′ multiome)

From fresh frozen tissues, nuclei isolation was performed as previously described in ref. ^157^, using low-retention microcentrifuge tubes (Fisher Scientific, Hampton, NH, USA) throughout the procedure to minimize nuclei loss. Briefly, OCT-embedded tissue fragments were rapidly thawed on wet ice to remove excess OCT, mechanically dissociated by pipetting, and homogenized in TST solution. The homogenate was filtered through a 30 µm MACS SmartStrainer (Miltenyi Biotec, Germany) and pelleted by centrifugation for 4 min at 500 × *g* at 4 °C. The resulting nuclei pellet was resuspended in 150 µL of 10× Genomics Diluted Nuclei Buffer. Trypan blue-stained nuclei were counted manually using INCYTO C-Chip Neubauer Improved Disposable Hemacytometers (VWR International Ltd., Radnor, PA, USA).

For 3′ RNA-seq, approximately 8000 nuclei per sample were loaded per channel of a Chromium Next GEM Chip G and processed on the Chromium Controller (10x Genomics, Pleasanton, CA, USA), followed by cDNA synthesis and library construction according to the manufacturer’s protocol (Chromium Next GEM Single Cell 3ʹ Reagent Kits v3.1 User Guide, Rev D). Libraries were normalized and pooled for sequencing on a NovaSeq SP-100 flow cell (Illumina, Inc., San Diego, CA, USA).

For multiome (RNA + ATAC) profiling, approximately 16,000–25,000 nuclei per sample were loaded per channel of a Chromium Next GEM Chip J and processed similarly using the Chromium Controller, followed by simultaneous transposition, cDNA synthesis, and library preparation as per the manufacturer’s instructions (Chromium Next GEM Single Cell Multiome ATAC + Gene Expression User Guide, Rev F). Libraries were normalized and pooled for sequencing across two NovaSeq SP-100 flow cells (Illumina, Inc.)

### Single-nucleus RNA sequencing (10× flex fixed)

FFPE tissue processing and RNA isolation were performed using the RNeasy FFPE Kit (Qiagen) on two 10-μm scrolls per sample, following the manufacturer’s protocol. RNA quality was assessed via DV200 analysis using the Agilent 2100 Bioanalyzer. For nuclei isolation, three 30-μm scrolls per sample were deparaffinized in xylene, rehydrated through graded ethanol washes, and incubated in Milli-Q water followed by cold PBS. Tissue dissociation was performed using enzymes from the Miltenyi FFPE Tissue Dissociation Kit and Liberase™ TM, followed by mechanical dissociation with the gentleMACS Octo Dissociator. Nuclei were filtered, centrifuged, and resuspended in 10x Genomics quenching buffer, and counted using AO staining with a Cellometer-K2 Fluorescent Cell Counter. All steps were performed independently for each of the nine samples.

Approximately one million nuclei per sample were hybridized with Human WTA Probes (10x Genomics) for 23 h at 42 °C, washed three times per the manufacturer’s protocol (CG000527-Rev E), and counted. Around 50,000 nuclei per sample were pooled, filtered, and assessed for quality. A total of ~144,000 pooled nuclei were processed using the Chromium Next GEM Single Cell Fixed RNA for Multiplexed Samples workflow. GEM formation, reverse transcription, cleanup, and pre-amplification were performed according to the user guide, followed by FLEX-GEX library construction. Final libraries were quantified on the Agilent Bioanalyzer 2100.

### Single-nucleus RNA sequencing data processing and analysis

Sequencing data were demultiplexed, aligned, and quantified using the 10x Genomics Cell Ranger pipeline (v3.1.0). Reads were aligned to the GRCh38 reference genome (Gencode annotation), and gene-by-cell UMI count matrices were generated. Count matrices were loaded into R using the Read10x function and processed using the Seurat v4^158^ package. Initial quality control retained features expressed in at least 3 cells, and cells with >200 and <3000 detected features. Cells with >5% mitochondrial gene content and those flagged as potential doublets using the Scrublet^159^ Python package (default parameters) were excluded.

Filtered data were normalized using Seurat’s NormalizeData function (scaling factor = 10,000), and the top 2000 variable features were identified per sample using FindVariableFeatures. All genes were used for scaling (ScaleData) and principal component analysis (PCA). PCs were manually reviewed per sample based on variance explained and top feature loadings to guide downstream dimensionality reduction and clustering. Clustering was performed using the Louvain algorithm at multiple resolutions (0.2–1.2); resolution was selected manually based on marker gene expression patterns within subclusters.

Broad lineage identities (tumor, lymphoid, myeloid, endothelial, fibroblast, normal epithelial) were assigned to clusters using canonical marker genes (e.g., Tumor: *TRIM63*, *GPNMB*, *NMRK2*, *CTSK*; Myeloid: *SLC8A1*, *CD74*, *CD163*, *CTSB*; Lymphoid: *THEMIS*, *PTPRC*, *IKZF1*, *CD96*; Endothelial: *PECAM1*, *FLT1*, *VWF*, *PTPRB*; Fibroblasts: *COL3A1*, *COL1A1*, *FBN1*, *LUM*). An additional reference-based cell annotation was performed using SingleR^160^ with the Blueprint-ENCODE reference. For the normal kidney sample (TRCC19N), nephron-specific cell types were annotated using marker genes from ref. ^58^.

For merged analysis across all samples, individual Seurat objects were combined using MergeData without batch correction. The merged dataset was reprocessed to identify highly variable genes, followed by PCA, UMAP, and clustering. For tumor-specific subcluster integration (Fig. 3), batch correction was performed using Harmony^161^ with batch and patient as variables. Module scores were computed using the AddModuleScore function across all snRNA-seq analyses. Large-scale copy number alterations were assessed at sample level using inferCNV^162^ algorithm, by using Myeloid and Lymphoid compartments are reference.

Differentially expressed genes between clusters were identified using FindAllMarkers with the following criteria: expression in ≥10% of cells within a cluster and a log_2_ fold-change >0.25, yielding a total of 2161 marker genes (Fig. 1C). To assess tumor cell-of-origin (Figure S3), FindTransferAnchors and TransferData functions were used to map tumor cells to nephron epithelial subtypes. Cell–cell communication analysis (Figs. 5, 6, and S7) was performed using the LIANA^163^ framework with default parameters, incorporating multiple tools (NATMI, Connectome, logFC, SCA, CellPhoneDB), and prioritizing ligand–receptor interactions with a consensus aggregate rank ≤ 0.05.

### Tumor meta-program NMF analysis

NMF analysis was performed separately for malignant cells from each tumor sample. For each tumor, raw gene expression counts were normalized using Seurat’s NormalizeData() function with default parameters. Highly variable genes (HVGs) were identified using FindVariableFeatures() (top 2000 genes), and their expression values were center-scaled using ScaleData(), regressing out the percentage of mitochondrial gene expression. Any negative values in the scaled expression matrix were set to zero.

NMF was then applied to the scaled HVG expression matrix using the “nsNMF” method (implemented in the NMF R package v0.23.0), across a range of factorization ranks from 5 to 25, as described previously^72^. To define robust gene modules, we implemented a previously described ranking strategy^46^: first, we generated two ranking matrices: (1) for each factor, genes were ranked by contribution weight; (2) for each gene, contributing factors were ranked by weight. A gene was included in a module if it ranked highest for a given factor, and genes were added sequentially until one was encountered that ranked higher for another factor. Modules with fewer than five genes were discarded, and this process was iteratively applied for each NMF rank.

To select an optimal rank, we identified the highest rank at which the number of resulting gene modules equaled the rank, ensuring stability and interpretability. The resulting gene modules across individual tumors were then filtered to retain only those with at least 5% overlap (Jaccard index) with two or more modules from other tumors, indicating shared transcriptional programs. Modules with fewer than five genes or those lacking significant enrichment for known gene sets were excluded from further analysis.

For biological annotation of the remaining gene modules, we performed pathway and ontology enrichment analysis using the ClusterProfiler^164^ and EnrichR^165^ packages in *R*. Enrichment was assessed against Gene Ontology terms, KEGG pathways, Reactome pathways, Hallmark gene sets (MSigDB), and PanglaoDB cell type markers.

To assess the distribution of meta-programs across individual samples, each malignant cell was annotated with the NMF module for which it exhibited the highest module score, calculated using Seurat’s AddModuleScore() function. To facilitate cross-cohort comparisons, tumor subclusters within the tRCC dataset were annotated with corresponding meta-programs that showed dominant activity within each subcluster. These annotations were based on enrichment of module scores and expression coherence. To compare our NMF-derived programs with previously published transcriptional programs, we retrieved gene signatures from published datasets and calculated module scores for these signatures across our NMF clusters.

### snRNA-seq regulon analysis

To infer transcription factor (TF) regulons and quantify their activity across tumor NMF clusters from snRNA-seq data, we applied the pySCENIC^166^ pipeline (v0.12.1) on subsetted tumor cells from merged object. Gene co-expression modules were first generated using the grn step, based on the filtered expression matrix and a curated hg38-based list of human TFs. These initial co-expression modules were then refined during the ctx step by evaluating motif enrichment in regulatory regions located +/− 10 kb from transcription start sites, using the hg38 motif ranking database.

To ensure biological relevance, only regulons with at least five target genes, a normalized enrichment score (NES) ≥ 2, and an area under the curve (AUC) threshold ≥ 0.1 were retained. Dropout masking was applied during this step to mitigate the effects of sparsity in single-nucleus RNA-seq data. Subsequently, regulon activity scores were calculated on a per-cell basis using the aucell step, producing a cell-by-regulon AUC matrix.

Differential regulon activity between epithelial-to-mesenchymal transition (EMT)-like and proximal tubule (PT)-like tumor subpopulations, defined based on tumor subclusters in Fig. 3D, was assessed using the FindMarkers function in Seurat. Regulons were ranked based on their average log fold change and adjusted *p*-value within EMT and PT clusters to identify TFs potentially driving lineage-specific transcriptional programs.

### snATAC-seq data analysis

All snATAC-seq samples were initially processed using Cell Ranger ATAC (v2.0, 10x Genomics) with default parameters, aligning to the GRCh38 reference genome (refdata-cellranger-arc-GRCh38-2020-A-2.0.0). Output barcode and fragment files were subsequently analyzed using the Signac^167^ R package (v1.12.0). Peak calling was performed with the CallPeaks() function using MACS2^168^. Quality control thresholds were applied based on the following parameters: nCount_RNA (1000 <count <25000), blacklist_fraction, nucleosome_signal, and TSS.enrichment, each filtered at +/− 3 median absolute deviations from the median per sample. Following QC, peak counts were normalized using TF-IDF normalization and dimensionality reduction was performed via Singular Value Decomposition using latent semantic indexing (LSI, components 2–30). Non-linear dimensionality reduction and clustering were then performed on the LSI components. Cluster annotation was carried out based on known chromatin accessibility markers and label transfer from matched snRNA-seq data. For integrated analyses, samples were harmonized using Harmony to correct for batch effects.

Differential chromatin accessibility between tRCC and ccRCC tumor cells was assessed using the FindMarkers() function in Signac. Genomic annotation of differentially accessible peaks was performed using the ClosestFeature() function to associate peaks with the nearest genes. Logistic regression was implemented to account for differences in sequencing depth across samples.

Motif enrichment analysis was then performed using AddMotifs() and the JASPAR2020 motif database. Top differentially accessible peaks were selected based on thresholds of |avg_log2FC| ≥ 1 and adjusted *p*-value ≤ 0.05. Overrepresented motifs in tRCC and ccRCC tumor cells were identified using the FindMotifs() function.

### CosMx spatial data analysis

Five-micron (5 µm) FFPE tissue sections were mounted on Leica BOND PLUS slides (Leica Biosystems, Cat. S21.2113.A). Sample preparation followed the manufacturer’s protocol for the Human Universal Cell Characterization 1000 Plex Panel (Nanostring, Part #122000157) as outlined in the CosMx SMI Manual Slide Preparation Manual (MAN-10159-01). Slides were initially baked overnight at 60 °C to enhance tissue adherence. Subsequent sample processing steps included sequential deparaffinization, target retrieval (15 min at 100 °C), and permeabilization with proteinase K (3 µg/mL for 15 min at 40 °C). Fiducials were then applied, followed by post-fixation and NHS-acetate treatment. Tissue sections were hybridized with denatured probes from the universal panel and the default add-on panel for 18 h at 37 °C.

After hybridization, slides were washed and stained with DAPI (15 min at room temperature) and a marker stain mix containing PanCK, CD45, CD68, and CD298/B2M (for cell segmentation). Slides were washed again and loaded onto the CosMx SMI instrument (Machine ID: CosMx_0020; Serial Number: INS2301H0020) for UV bleaching, cyclic imaging, and scanning. Raw images were processed and decoded using the default pipeline on Atomx SIP, a cloud-based service. The resulting Nanostring spatial transcriptomics data were imported into *R* and analyzed using the Seurat package. Cell type annotation was performed by label transfer from matched single-nucleus RNA-seq datasets and further validated by differential gene expression analysis using the FindMarkers() function. EMT program was identified based on module score analysis and cells with positive scores were considered to be EMT positive.

For spatial proximity analysis, cell centroid coordinates were extracted from the CosMx Seurat object using GetTissueCoordinates(). For each pairwise interaction of interest, nearest-neighbor distances were computed from each cell of the sender cell type to the closest cell of the receiver cell type using the nn2() function from the RANN R package. Distances were converted from pixels to microns using the CosMx platform scaling factor (0.18 µm/pixel). The fraction of sender cells residing within 20 µm of a receiver cell was reported as the proximity co-localization metric. This threshold was selected to capture direct cell–cell contact and proximal paracrine signaling, consistent with the spatial range of the ligand-receptor pairs examined.

### Bulk-RNA data analysis

Data from external tRCC cohorts were obtained as previously described^12^ for TCGA^35^ (with sample annotations from Chen Creighton et al.), refs. ^36,37^. Raw tRCC sequencing data and metadata for refs. ^22,23^ were obtained from the respective studies. Reads were aligned to the human reference genome (GRCh38.p13 assembly) using STAR (v2.7.10a) and quantified as paired-end reads against the GENCODE v45 transcript reference using RSEM (v1.3.1). Expression matrices from each dataset were log-transformed, centralized, and combined to reduce batch-related differences prior to downstream analysis. For immune deconvolution analysis, we used TIMER2.0^169^, and the results plotted are from TIMER and CIBERSORTx absolute mode. Expression scores for different meta-programs were identified by calculating average expression of genes comprising each meta-program (Supplementary Data 3).

To evaluate the clinical relevance of transcriptional programs and immune cells, we used gene expression and survival data from ref. ^23^. to calculate program scores and assess their association with patient outcomes (Fig. 5). Program scores were derived as the average expression of genes comprising each meta-program after log-transformation and centralization of the expression matrix. We performed multivariate survival analysis using Cox proportional hazards models (coxph function, survival R package), adjusting for clinical and molecular covariates including SPP1 macrophage signature (*SPP1*/*CD68*), EMT status (based on NMF signature; Supplementary Data 3), matrix CAFs (*COL1A1*/*POSTN*), fusion partner (categorized as sample with ASPSCR1-TFE3 fusion vs other partners), and tumor stage (categorized as early [I-II] vs. advanced [III-IV]). Kaplan–Meier curves were generated to compare overall survival (OS) between stratified groups, SPP1 macrophage high vs. low (dichotomized at the median), and statistical significance was evaluated using the log-rank test (survminer v0.4.9).

### tRCC vs ccRCC comparative data analysis

For comparative transcriptomic analysis between tRCC and ccRCC, we utilized both snRNA-seq and bulk RNA-seq datasets. For the snRNA-seq analysis, we subsetted tumor cells from tRCC and ccRCC samples; where ccRCC data were obtained from ref. ^38^., processed in alignment with their published pipeline, and restricted to the cells included in their original study. The resulting Seurat objects (from fresh frozen samples) were merged, and differential gene expression analysis was performed using the FindMarkers function in Seurat, applying the Wilcoxon rank-sum test. Genes with an average log fold change ≥ 1.5 and adjusted *p*-value < 0.05 were considered significantly differentially expressed.

For bulk RNA-seq data, expression matrices from each dataset^22,35,36^ were log-transformed and centralized. These normalized datasets were then combined, and differential expression between tRCC and ccRCC was assessed by comparing gene-wise *z*-scores and Wilcoxon test with benjamini-hochberg correction. Genes with a *z*-score difference ≥ 0.5 and adjusted *p*-value < 0.05 were considered significant.

Pathway enrichment analysis was performed on the set of intersecting differentially expressed genes identified in both snRNA-seq and bulk RNA-seq platforms using ClusterProfiler^164^ (v4.13.3).

### tRCC vs ccRCC cell–cell interaction analysis

To enable unbiased comparison of cell–cell communication between tRCC and ccRCC, snRNA-seq data were downsampled prior to interaction inference to control for differences in cell number and transcriptional complexity (nFeature_RNA) across datasets. Four cellular lineages were retained for analysis: Tumor, Macrophages, Fibroblasts, and Lymphoid cells. Downsampling was performed via a nFeature_RNA-matched binning procedure applied independently to each lineage. Briefly, nFeature_RNA distributions from both conditions were pooled and partitioned into ten equal-quantile bins with shared boundaries, and within each bin, cells were randomly sampled without replacement from the larger condition to match the smaller. This procedure was repeated across 100 independent iterations, yielding per-iteration matched subsets of 30241 Tumor, 2391 Macrophage, 1530 Fibroblast, and 921 Lymphoid cells per condition, with tRCC constituting the limiting condition for all lineages except Fibroblasts.

Cell–cell interaction analysis was performed independently on each of the 100 downsampled iterations using CellPhoneDBv5^170^, with the directional sender-receiver pair combined with the ligand-receptor (LR) pair defined as the primary unit of analysis. An interaction was considered detected in a given iteration if it appeared in the significant means output. To derive a consensus interaction set reflecting genuine biological signal rather than sampling artifacts, only interactions detected in at least 90 of 100 iterations were retained.

### TFE3-fusion transcriptional signature

The TFE3-fusion signature was derived using previously published bulk RNA-seq datasets from HEK-293T cells engineered to overexpress various TFE3 fusion constructs (ASPSCR1-TFE3, SFPQ-TFE3, NONO-TFE3, and PRCC-TFE3^12^) compared to mock-transfected controls, along with data from the tRCC cell line FUUR1^30^ following shRNA-mediated knockdown of the ASPSCR1-TFE3 fusion. Differentially expressed genes were first identified in the overexpression datasets by filtering for those with a fold change ≥ 2 and *p*-value ≤ 0.05 in at least two of the fusion constructs. This gene set was intersected with genes significantly downregulated upon TFE3 fusion knockdown in FUUR1 to define a consensus fusion-driven program, yielding a core list of 113 genes (Supplementary Data 3). The resulting TFE3-fusion signature was then used to quantify TFE3 fusion activity across datasets: for snRNA-seq, scores were calculated per nucleus using the AddModuleScore function in Seurat; for bulk RNA-seq data, activity scores were computed as the average expression of the signature genes after log-normalization and centering.

### T cell receptor sequencing and analysis

TCR sequencing was performed using the rhTCRseq protocol for bulk TCR sequencing and repertoire analysis as previously described in ref. ^171^. For data analysis, raw FASTQ files were processed using an in-house rhTCRseq pipeline. Reads were first aligned to either the TRA or TRB locus using BLAST and subsequently assembled into TCR clonotypes using MiXCR. CDR3 sequences were extracted, and clonotypes were collapsed based on CDR3 similarity to generate a final list of unique clonotypes and their respective frequencies. TCR sequencing data is provided in Supplementary Data 4.

### Multiplex immunoflourescence

An optimized multiplex immunofluorescence (mIF) panel was performed on 4 μm thick sections cut from formalin-fixed paraffin-embedded (FFPE) tumor samples. Tissue specimens from eleven patients with clear cell renal cell carcinoma (ccRCC) and ten patients with translocation renal cell carcinoma (tRCC) were stained using a 7-plex mIF panel consisting of DAPI, CD163, SPP1, MRC1, CD86, CD8, and PD1. Detailed information of the antibodies and dyes are mentioned in Supplementary Data 5. Automated staining was performed on a BOND Rx Autostainer (Leica Biosystems) utilizing the BOND Polymer Refine Detection Kit (Leica Biosystems) in combination with the Opal Tyramide Signal Amplification system (Akoya Biosciences).

Following staining, slides were scanned using the PhenoImager HT system (Akoya Biosciences, v2.0.0) for whole-slide multispectral images acquisition. Specific regions of interest (ROIs) were selected using Phenochart software (v2.0.0, Akoya Biosciences), followed by spectral unmixing in the InForm software (v2.6.0, Akoya Biosciences) using spectral libraries generated from monoplex-stained controls. Unmixed multispectral images were subsequently imported into the HALO platform (v4.0.5107.407; Indica Labs) for visualization and downstream analysis. Tumor areas were manually annotated by two pathologists (GRO and YNL) excluding necrotic areas, hemorrhage, and thick fibrous septa. The Indica Labs High-Plex FL module was used to perform DAPI-based nuclear segmentation and to set a specific threshold for each marker. A unique analysis algorithm was generated for each case and validated by two pathologists (GRO and YNL) to reach a final consensus.

For macrophage population analysis, at least three tumor ROIs (2790 × 2094 µm) enriched in CD163+ cells were selected per case. The following phenotypes were identified and quantified: SPP1+ macrophages (CD163 + SPP1+) and MRC1-positive macrophages (CD163 + MRC1+). For lymphocyte population analysis, at least five tumor regions (930 × 698 µm) enriched in CD8+ T cells were selected per case. PD-1-expressing T cells (CD8+PD-1+) phenotype was identified and quantified. Cell densities (number of cells/mm²) and respective percentages of immune cell phenotypes were calculated.

### Wound healing assay

FUUR1 cells stably transduced with a doxycycline-inducible shTFE3 construct (pLV708, shRNA: AATCAGATAAACAAATGAGGG) were seeded at 100,000 cells per well in 6-well plates and treated with doxycycline (2 μg/mL) or left untreated for 96 h to induce TFE3 knockdown prior to wounding. Wounds were generated by scratching the cell monolayer with a 20 μL pipette tip, after which cells were washed once with PBS to remove debris. Wound closure was monitored at 1, 8, 20, and 30 h post-scratch using a Celigo Image Cytometer (Nexcelom Bioscience), which was used to quantify the percentage of open wound area remaining at each timepoint using FIJI v2.16.0. The experiment was performed with 3 biological replicates and 2 technical replicates per condition. Geneticin selection (800 μg/mL) was maintained during cell expansion but was omitted during the wound healing experiment itself. All the constructs were confirmed by Sanger sequencing.

### Statistical analysis and reproducibility

Quantification and statistical analyses were performed as described in the corresponding figure legends and throughout the text and methods section. Where applicable, *p*-values, the statistical tests used to generate them, and the exact number of samples or nuclei (N/n) are indicated in the figure legends and the main text. Spatial transcriptomic analyses were performed on one section per independent tRCC tumor sample. Figure 6B shows sample TRCC6, and Fig. 6D shows sample TRCC14.

### Reporting summary

Further information on research design is available in the Nature Portfolio Reporting Summary linked to this article.

## Supplementary information

Supplementary Information

Description of Additional Supplementary Files

Supplementary Data 1

Supplementary Data 2

Supplementary Data 3

Supplementary Data 4

Supplementary Data 5

Reporting Summary

Transparent Peer Review file

## Source data

Source Data

## Data Availability

The de-identified single-nucleus sequencing data generated in this study, including both raw and processed data, have been deposited in the NCBI Gene Expression Omnibus (GEO) under accession codes GSE307120 and GSE269466. T cell receptor sequencing data is provided within the paper in supplementary data 4. Source data underlying the figures are provided with this paper in the accompanying source data files. Previously generated RNA-sequencing and chromatin immunoprecipitation sequencing data from tRCC cell line models are publicly available in GEO under accession codes GSE266517 and GSE266530, respectively. The previously published HEK293T cell line dataset is available under accession code GSE188885. The dataset reported by ref. ^22^ is available in GEO under accession code GSE167573. Data from The Cancer Genome Atlas (TCGA) Pan-Kidney cohort were obtained through the National Cancer Institute Genomic Data Commons^35^ [https://portal.gdc.cancer.gov/]. Data from the IMmotion151 cohort were obtained from European genome-phenome archive under accession number EGAS00001004353, and data from ref. ^37^ was obtained from the publication associated supplementary table [https://pathsocjournals.onlinelibrary.wiley.com/doi/10.1002/path.5470]. The RNA-sequencing data reported by ref. ^23^ are available through the National Omics Data Encyclopedia under project identifier OEP002630 [https://www.biosino.org/node/project/detail/OEP00002630]. Source data are provided with this paper.

## Code availability

No new computational tools or custom code/ algorithms were developed for this study; all analytical methods are described in detail within the “Methods” section.
