# Supplementary material for: Fusion-driven oncogenic programs shape the immune landscape in translocation renal cell carcinoma
Source: Nat Commun. 2026 Aug 26;17:10191. doi: 10.1038/s41467-026-76858-w (PMC13612822; doi:10.1038/s41467-026-76858-w)
Supplement: Supplementary file 1 [file 41467_2026_76858_MOESM1_ESM.pdf]

SUPPLEMENTARY INFORMATION

FUSION-DRIVEN ONCOGENIC PROGRAMS SHAPE THE IMMUNE LANDSCAPE IN TRANSLOCATION RENAL CELL CARCINOMA

Prathyusha Konda[1,2], Alexandra J. Wakolbinger[1*], Yantong Cui[1*], Gabriel Roberti de Oliveira[3], Yasmin L. Nabil[3], Cary N. Weiss[4,5], Maxwell D. Seager[3], Riva Deodhar[1], Jiao Li[1,2], Sayed Matar[3], Jinyu Wang[6], Jack Horst[1], Sabrina Y. Camp[1], Aseman B. Sheshdeh[5], Jonathan L. Hecht[7], David J. Einstein[8], Yashika Rustagi[1], Anwesha Nag[1], Aaron R. Thorner[1], Cheng-Zhong Zhang[6], Eliezer M. Van Allen[1,2,9], Sabina Signoretti[3], Toni K. Choueiri[1,2,10 #], Srinivas R. Viswanathan[1,2,10,#].

1 Department of Medical Oncology, Dana-Farber Cancer Institute, Boston, MA 02215, USA

2 Department of Medicine, Harvard Medical School, Boston, MA 02215, USA

3 Department of Pathology, Brigham and Women's Hospital, Boston, MA 02215, USA

4 Department of Pediatric Oncology, Dana-Farber Cancer Institute, Boston, MA 02215, USA

5 Division of Hematology/Oncology, Boston Children's Hospital; Harvard Medical School, Boston, MA, USA

6 Department of Biomedical Informatics, Dana-Farber Cancer Institute, Boston, MA 02215, USA

7 Department of Pathology, Beth Israel Deaconess Medical Center, Boston, MA 02215, USA

8 Division of Medical Oncology, Beth Israel Deaconess Medical Center, Boston, MA 02215, USA

9 Broad Institute of MIT and Harvard, Cambridge, MA 02142, USA

10 Department of Medicine, Brigham and Women's Hospital; Boston, MA 02215, USA

* These authors contributed equally

# To whom correspondence should be addressed:

Srinivas R. Viswanathan: srinivas_viswanathan@dfci.harvard.edu

Toni K. Choueiri: toni_choueiri@dfci.harvard.edu

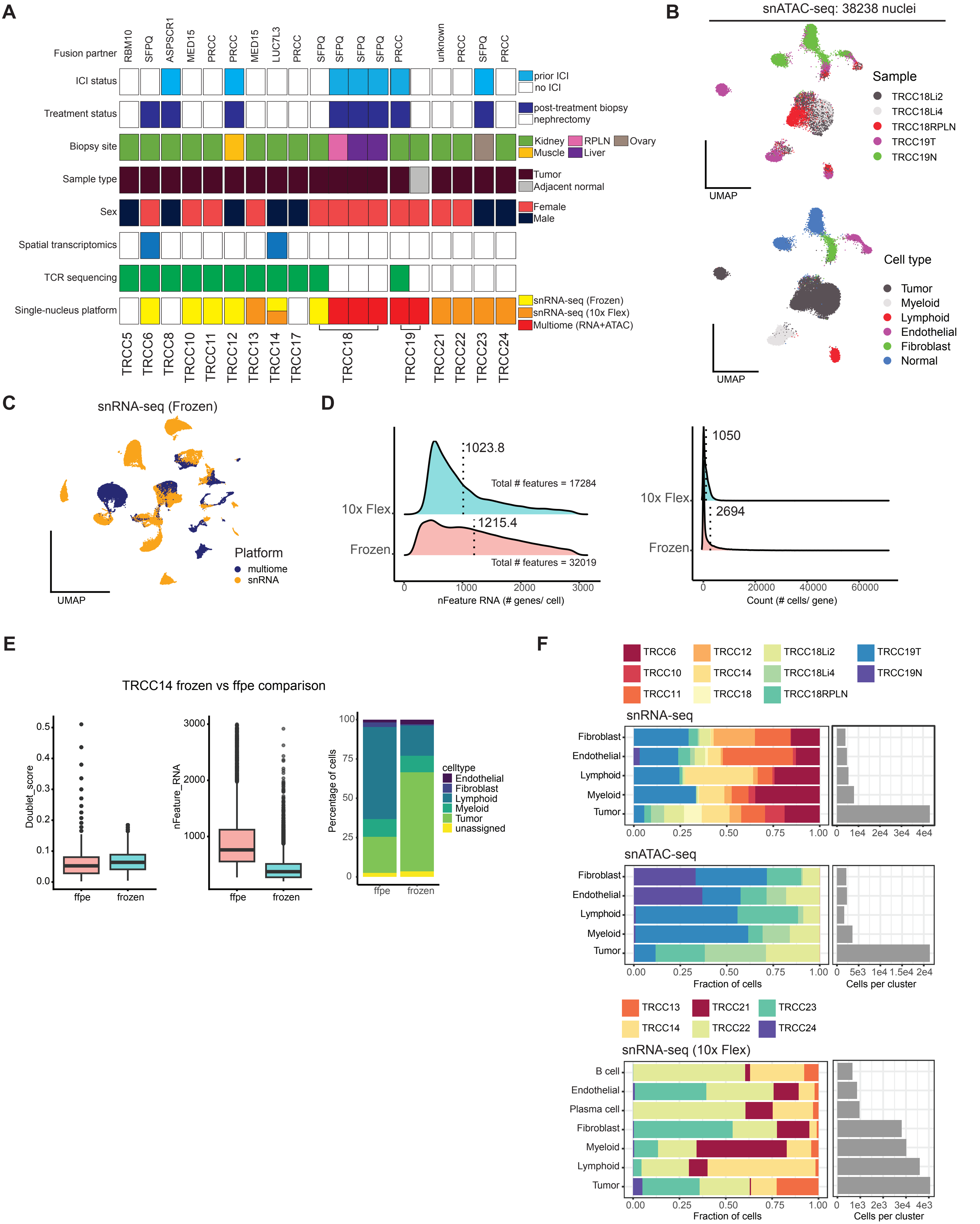
A
Fusion partner
RBM10 SFPQ ASPSCR1 MED15 PRCC PRCC MED15 LUC7L3 PRCC SFPQ SFPQ SFPQ SFPQ PRCC unknown PRCC SFPQ PRCC
ICI status
prior ICI
no ICI
Treatment status
post-treatment biopsy
nephrectomy
Biopsy site
Kidney RPLN Ovary
Muscle Liver
Sample type
Tumor
Adjacent normal
Sex
Female
Male
Spatial transcriptomics
TCR sequencing
Single-nucleus platform
snRNA-seq (Frozen)
snRNA-seq (10x Flex)
Multiome (RNA+ATAC)
TRCC5 TRCC6 TRCC8 TRCC10 TRCC11 TRCC12 TRCC13 TRCC14 TRCC17 TRCC18 TRCC19 TRCC21 TRCC22 TRCC23 TRCC24
B
snATAC-seq: 38238 nuclei
Sample
TRCC18Li2
TRCC18Li4
TRCC18RPLN
TRCC19T
TRCC19N
UMAP
Cell type
Tumor
Myeloid
Lymphoid
Endothelial
Fibroblast
Normal
UMAP
C
snRNA-seq (Frozen)
Platform
multiome
snRNA
UMAP
D
1023.8
Total # features = 17284
10x Flex
1215.4
Frozen
Total # features = 32019
0 1000 2000 3000
nFeature RNA (# genes/ cell)
1050
10x Flex
2694
Frozen
0 20000 40000 60000
Count (# cells/ gene)
E
TRCC14 frozen vs ffpe comparison
Doublet_score
ffpe frozen
nFeature_RNA
ffpe frozen
Percentage of cells
ffpe frozen
celltype
Endothelial
Fibroblast
Lymphoid
Myeloid
Tumor
unassigned
F
TRCC6 TRCC12 TRCC18Li2 TRCC19T
TRCC10 TRCC14 TRCC18Li4 TRCC19N
TRCC11 TRCC18 TRCC18RPLN
snRNA-seq
Fibroblast
Endothelial
Lymphoid
Myeloid
Tumor
0 1e4 2e4 3e4 4e4
snATAC-seq
Fibroblast
Endothelial
Lymphoid
Myeloid
Tumor
Fraction of cells
0 5e3 1e4 1.5e4 2e4
Cells per cluster
TRCC13 TRCC21 TRCC23
TRCC14 TRCC22 TRCC24
snRNA-seq (10x Flex)
B cell
Endothelial
Plasma cell
Fibroblast
Myeloid
Lymphoid
Tumor
Fraction of cells
0 1e3 2e3 3e3 4e3
Cells per cluster

Figure S1. Characteristics of tRCC snRNA-seq cohorts

A. Summary of clinicopathological characteristics, treatment history, biopsy site, fusion partner, and molecular profiling approaches used for tRCC samples in this study.
B. UMAPs of tRCC snATAC-seq data representing samples (top) and major cell lineages (bottom).
C. UMAP representation of snRNA-seq data from the frozen cohort, highlighting samples processed via multiome (RNA+ATAC) or RNA-only platforms.
D. Distribution of number of genes detected per nucleus in frozen and 10x flex fixed cohorts (left) and number of cells in which each gene was detected (right).
E. Box plots comparing doublet score (left), nfeature_RNA (middle), and stacked barplot showing lineage composition (right) in TRCC14 frozen vs ffpe sample. Data is represented as median with 25th and 75th percentiles defining the box limits.
F. Stacked bar plots showing lineage composition in three tRCC cohorts: snRNA-seq (Frozen), snATAC-seq, and snRNA-seq (10x Flex). Adjacent bar plots indicate total cell counts per lineage.

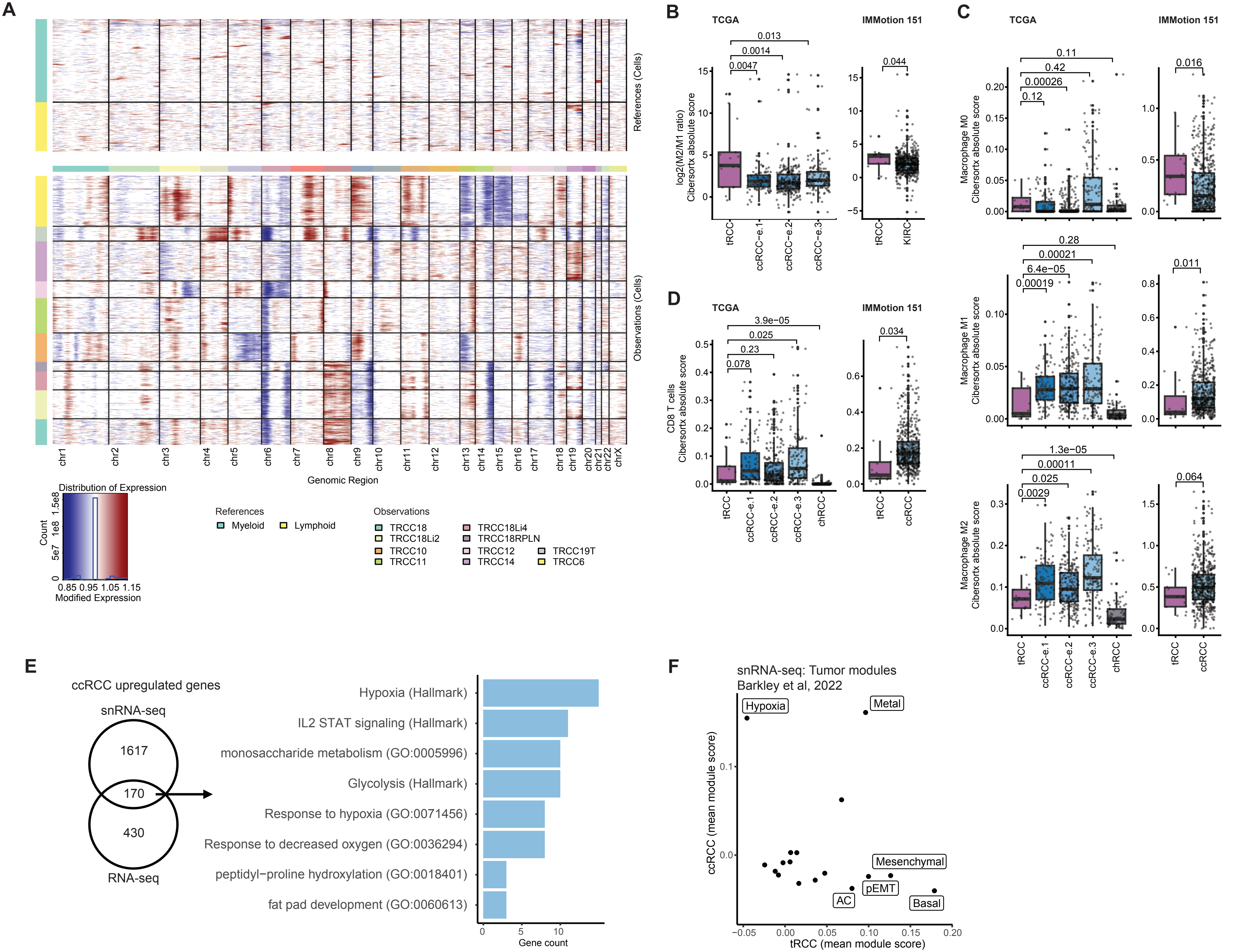
A
References (Cells)
Observations (Cells)
chr1
chr2
chr3
chr4
chr5
chr6
chr7
chr8
chr9
chr10
chr11
chr12
chr13
chr14
chr15
chr16
chr17
chr18
chr19
chr20
chr21
chr22
chrX
Genomic Region
Distribution of Expression
Count
0
5e7
1e8
1.5e8
0.85 0.95 1.05 1.15
Modified Expression
References
Myeloid
Lymphoid
Observations
TRCC18
TRCC18Li2
TRCC10
TRCC11
TRCC18Li4
TRCC18RPLN
TRCC12
TRCC14
TRCC19T
TRCC6
B
TCGA
IMMotion 151
log2(M2/M1 ratio)
Cibersortx absolute score
0.013
0.0014
0.0047
0.044
tRCC
ccRCC−e.1
ccRCC−e.2
ccRCC−e.3
KIRC
C
TCGA
IMMotion 151
Macrophage M0
Cibersortx absolute score
0.11
0.42
0.00026
0.12
0.016
0.28
0.00021
6.4e−05
0.00019
0.011
Macrophage M1
Cibersortx absolute score
1.3e−05
0.00011
0.025
0.0029
0.064
Macrophage M2
Cibersortx absolute score
tRCC
ccRCC−e.1
ccRCC−e.2
ccRCC−e.3
chRCC
tRCC
ccRCC
D
TCGA
IMMotion 151
3.9e−05
0.025
0.23
0.078
0.034
CD8 T cells
Cibersortx absolute score
tRCC
ccRCC−e.1
ccRCC−e.2
ccRCC−e.3
chRCC
tRCC
ccRCC
E
ccRCC upregulated genes
snRNA-seq
1617
170
430
RNA-seq
Hypoxia (Hallmark)
IL2 STAT signaling (Hallmark)
monosaccharide metabolism (GO:0005996)
Glycolysis (Hallmark)
Response to hypoxia (GO:0071456)
Response to decreased oxygen (GO:0036294)
peptidyl−proline hydroxylation (GO:0018401)
fat pad development (GO:0060613)
0 5 10
Gene count
F
snRNA-seq: Tumor modules
Barkley et al, 2022
Hypoxia
Metal
Mesenchymal
pEMT
AC
Basal
ccRCC (mean module score)
tRCC (mean module score)
−0.05 0.00 0.05 0.10 0.15 0.20

Figure S2. Transcriptomic and immune landscape of tRCC and ccRCC

A. Inferred copy number variants of malignant cells from the tRCC frozen cohort in this study. Reference myeloid and lymphoid cell populations in the upper panel; tRCC tumor cell observations in the lower panel, grouped by sample identity.
B. Boxplot showing the log2(M2/ M1) ratio from cibersortx absolute scores identified through bulk RNA-seq deconvolution in RCC subtypes. Data is represented as median with 25th and 75th percentiles defining the box limits. P values were calculated using two-sided Wilcoxon rank-sum tests.
C. Boxplots showing the cibersortx absolute scores of macrophage cell types identified through bulk RNA-seq deconvolution in RCC subtypes. Data is represented as median with 25th and 75th percentiles defining the box limits. P values were calculated using two-sided Wilcoxon rank-sum tests.
D. Boxplot showing the cibersortx absolute scores of CD8 T cells identified through bulk RNA-seq deconvolution in RCC subtypes. Data is represented as median with 25th and 75th percentiles defining the box limits. P values were calculated using two-sided Wilcoxon rank-sum tests.
E. Venn diagram depicting genes upregulated in ccRCCs vs tRCC in snRNA-seq and bulk RNA-Seq datasets (left). Bar plot with top enriched pathways in ccRCCs based on gene-ontology (right).
F. Scatterplot of module scores in tRCC vs ccRCC tumor cells from snRNA-seq data, based on a published study[46].

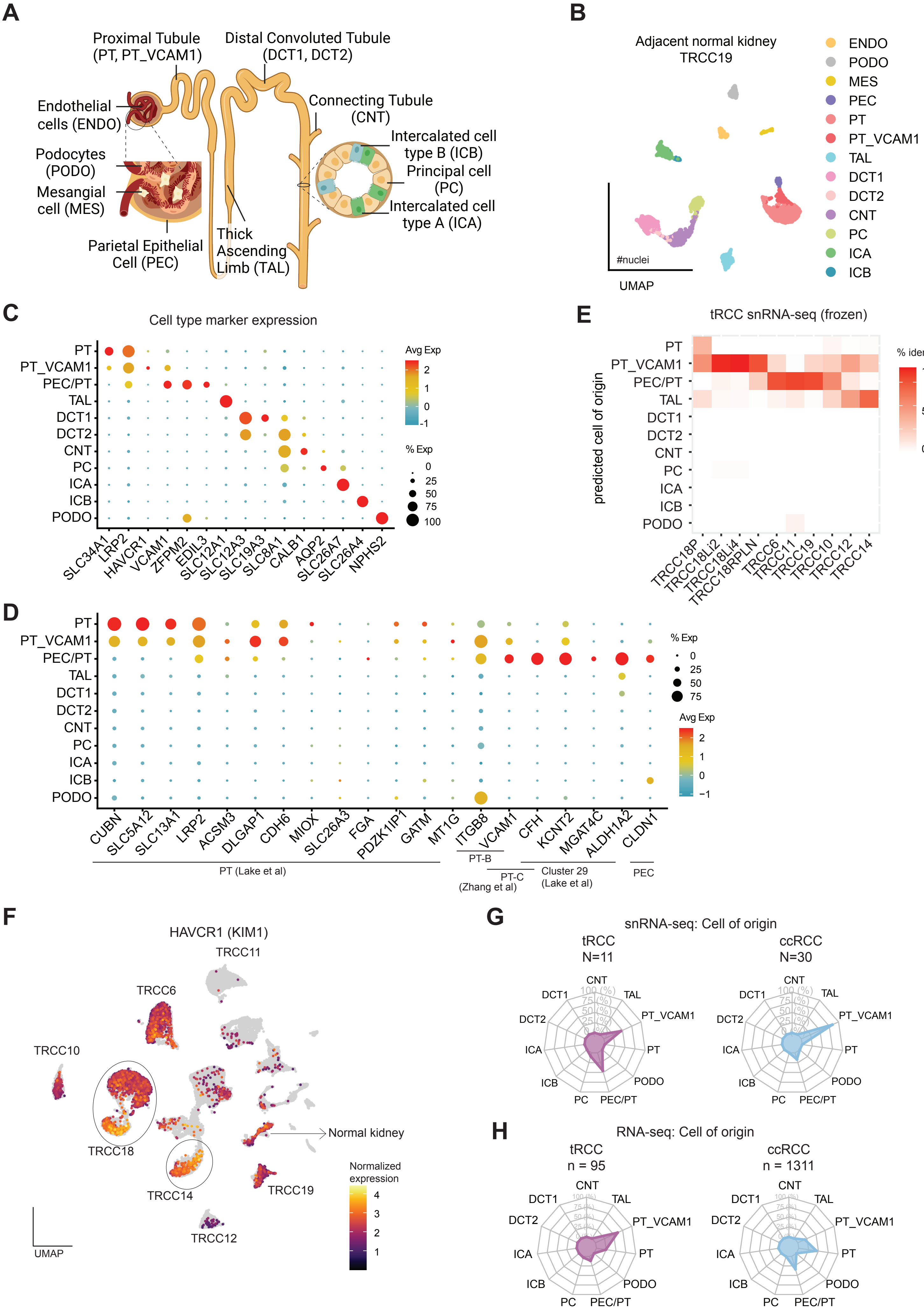

A
Proximal Tubule (PT, PT_VCAM1)
Distal Convoluted Tubule (DCT1, DCT2)
Endothelial cells (ENDO)
Connecting Tubule (CNT)
Intercalated cell type B (ICB)
Podocytes (PODO)
Principal cell (PC)
Mesangial cell (MES)
Intercalated cell type A (ICA)
Thick Ascending Limb (TAL)
Parietal Epithelial Cell (PEC)
B
Adjacent normal kidney TRCC19
ENDO PODO MES PEC PT PT_VCAM1 TAL DCT1 DCT2 CNT PC ICA ICB
#nuclei
UMAP
C
Cell type marker expression
PT PT_VCAM1 PEC/PT TAL DCT1 DCT2 CNT PC ICA ICB PODO
SLC34A1 LRP2 HAVCR1 VCAM1 ZFPM2 EDIL3 SLC12A1 SLC12A3 SLC19A3 SLC8A1 CALB1 AQP2 SLC26A7 SLC26A4 NPHS2
Avg Exp 2 1 0 -1
% Exp 0 25 50 75 100
E
tRCC snRNA-seq (frozen)
predicted cell of origin
PT PT_VCAM1 PEC/PT TAL DCT1 DCT2 CNT PC ICA ICB PODO
TRCC18P TRCC18Li2 TRCC18Li4 TRCC18RPLN TRCC6 TRCC11 TRCC19 TRCC10 TRCC12 TRCC14
% identity 100 50 0
D
PT PT_VCAM1 PEC/PT TAL DCT1 DCT2 CNT PC ICA ICB PODO
CUBN SLC5A12 SLC13A1 LRP2 ACSM3 DLGAP1 CDH6 MIOX SLC26A3 FGA PDZK1IP1 GATM MT1G ITGB8 VCAM1 CFH KCNT2 MGAT4C ALDH1A2 CLDN1
PT (Lake et al)
PT-B
PT-C (Zhang et al)
Cluster 29 (Lake et al)
PEC
% Exp 0 25 50 75
Avg Exp 2 1 0 −1
F
HAVCR1 (KIM1)
TRCC11
TRCC6
TRCC10
TRCC18
TRCC14
TRCC19
TRCC12
Normal kidney
Normalized expression 4 3 2 1
UMAP
G
snRNA-seq: Cell of origin
tRCC N=11
ccRCC N=30
CNT TAL PT_VCAM1 PT PODO PEC/PT PC ICB ICA DCT2 DCT1
100 (%) 75 (%) 50 (%) 25 (%) 0 (%)
H
RNA-seq: Cell of origin
tRCC n = 95
ccRCC n = 1311
CNT TAL PT_VCAM1 PT PODO PEC/PT PC ICB ICA DCT2 DCT1

Figure S3. tRCC and ccRCC tumors arise from a similar cell of origin

A. Schematic of human nephron depicting different normal kidney cell types.

B. UMAP from snRNA-seq of tumor-adjacent normal kidney sample, TRCC19N.

C. Dot plot depicting the expression of marker genes in normal kidney cell types in TRCC19N.

D. Dot plot depicting the expression of expanded marker gene panel in normal kidney cell types in TRCC19N, incorporating PT and cluster 29 markers from Lake et al, PT-B/PT-C markers from Zhang et al, and canonical PEC marker CLDN1.

E. Heatmap depicting percent identity between tumor cells in individual tRCC samples and normal kidney cell types (tRCC snRNA-seq frozen cohort).

F. UMAP showing expression of *HAVCR1* (KIM1) across tRCC tumor and non-tumor cells from the frozen cohort.

G. Radar plots indicating the similarity of tRCC and ccRCC to different normal kidney cell types (snRNA-seq frozen cohorts).

H. Radar plots indicating the similarity of samples from tRCC and ccRCC bulk RNA-seq cohorts to different normal kidney cell types.

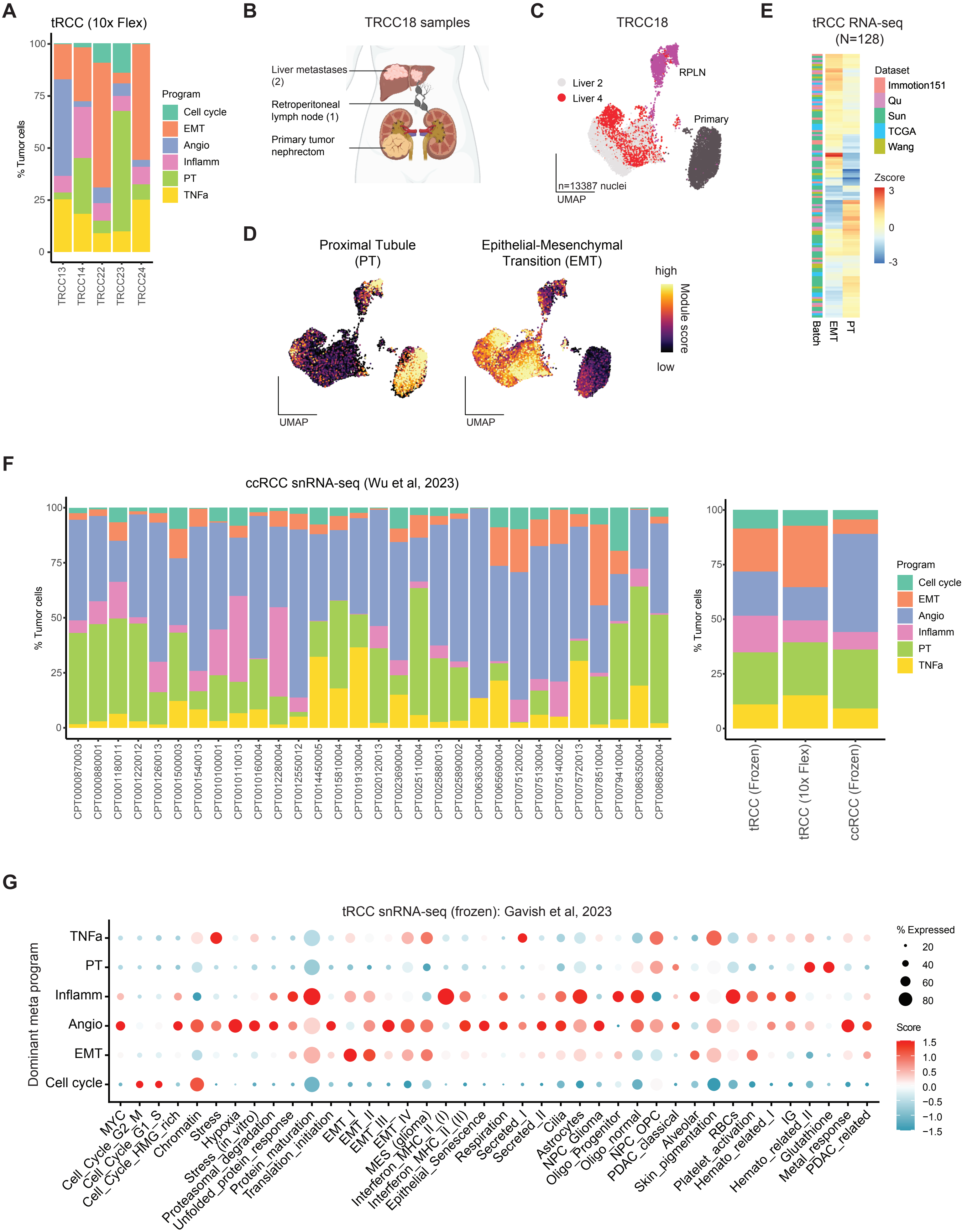

A
tRCC (10x Flex)
% Tumor cells
Program
Cell cycle
EMT
Angio
Inflamm
PT
TNFa
TRCC13
TRCC14
TRCC22
TRCC23
TRCC24
B
TRCC18 samples
Liver metastases (2)
Retroperitoneal lymph node (1)
Primary tumor nephrectom
C
TRCC18
Liver 2
Liver 4
RPLN
Primary
n=13387 nuclei
UMAP
D
Proximal Tubule (PT)
Epithelial-Mesenchymal Transition (EMT)
high
Module score
low
UMAP
E
tRCC RNA-seq (N=128)
Dataset
Immotion151
Qu
Sun
TCGA
Wang
Zscore
Batch
EMT
PT
F
ccRCC snRNA-seq (Wu et al, 2023)
% Tumor cells
CPT0000870003
CPT0000880001
CPT0001180011
CPT0001220012
CPT0001260013
CPT0001500003
CPT0001540013
CPT0010100001
CPT0010110013
CPT0010160004
CPT0012280004
CPT0012550012
CPT0014450005
CPT0015810004
CPT0019130004
CPT0020120013
CPT0023690004
CPT0025110004
CPT0025880013
CPT0025890002
CPT0063630004
CPT0065690004
CPT0075120002
CPT0075130004
CPT0075140002
CPT0075720013
CPT0078510004
CPT0079410004
CPT0086350004
CPT0086820004
tRCC (Frozen)
tRCC (10x Flex)
ccRCC (Frozen)
Program
Cell cycle
EMT
Angio
Inflamm
PT
TNFa
G
tRCC snRNA-seq (frozen): Gavish et al, 2023
Dominant meta program
TNFa
PT
Inflamm
Angio
EMT
Cell cycle
% Expressed
Score
MYC
Cell_Cycle_G2_M
Cell_Cycle_G1_S
Cell_Cycle_HMG_rich
Chromatin
Stress
Hypoxia
Stress_(in_vitro)
Proteasomal_degradation
Unfolded_protein_response
Protein_maturation
Translation_initiation
EMT_I
EMT_II
EMT_III
EMT_IV
MES_(glioma)
Interferon_MHC_II_(I)
Interferon_MHC_II_(II)
Epithelial_Senescence
Respiration
Secreted_I
Secreted_II
Cilia
Astrocytes
NPC_Glioma
Oligo_Progenitor
Oligo_normal
NPC_OPC
PDAC_classical
Alveolar
Skin_pigmentation
RBCs
Platelet_activation
Hemato_related_I
IG
Hemato_related_II
Glutathione
Metal_response
PDAC_related

Figure S4. Characterization of tumor programs in tRCC and ccRCC

A. Stacked bar plot representing the distribution of tRCC NMF programs across the tRCC 10x Flex cohort.
B. Schematic of samples related to primary and metastatic sites from patient TRCC18.
C. UMAP depicting distribution of cells from TRCC18 by biopsy site.
D. UMAP depicting proximal tubule (PT) and epithelial-mesenchymal transition (EMT) program scores across primary and metastatic sites of TRCC18.
E. Heatmap depicting distribution of EMT and PT program scores across tRCC samples from the bulk RNA-seq cohort, annotated by dataset.
F. Stacked bar plot representing the distribution of identified NMF programs across ccRCC snRNA-seq cohort (left) and comparison of overall proportions between all tRCC and ccRCC cohorts (right).
G. Dot plot showing module scores of tumor NMF programs defined previously in a pan-cancer study by Gavish et al.[45] and association with tRCC NMF programs identified in this study.

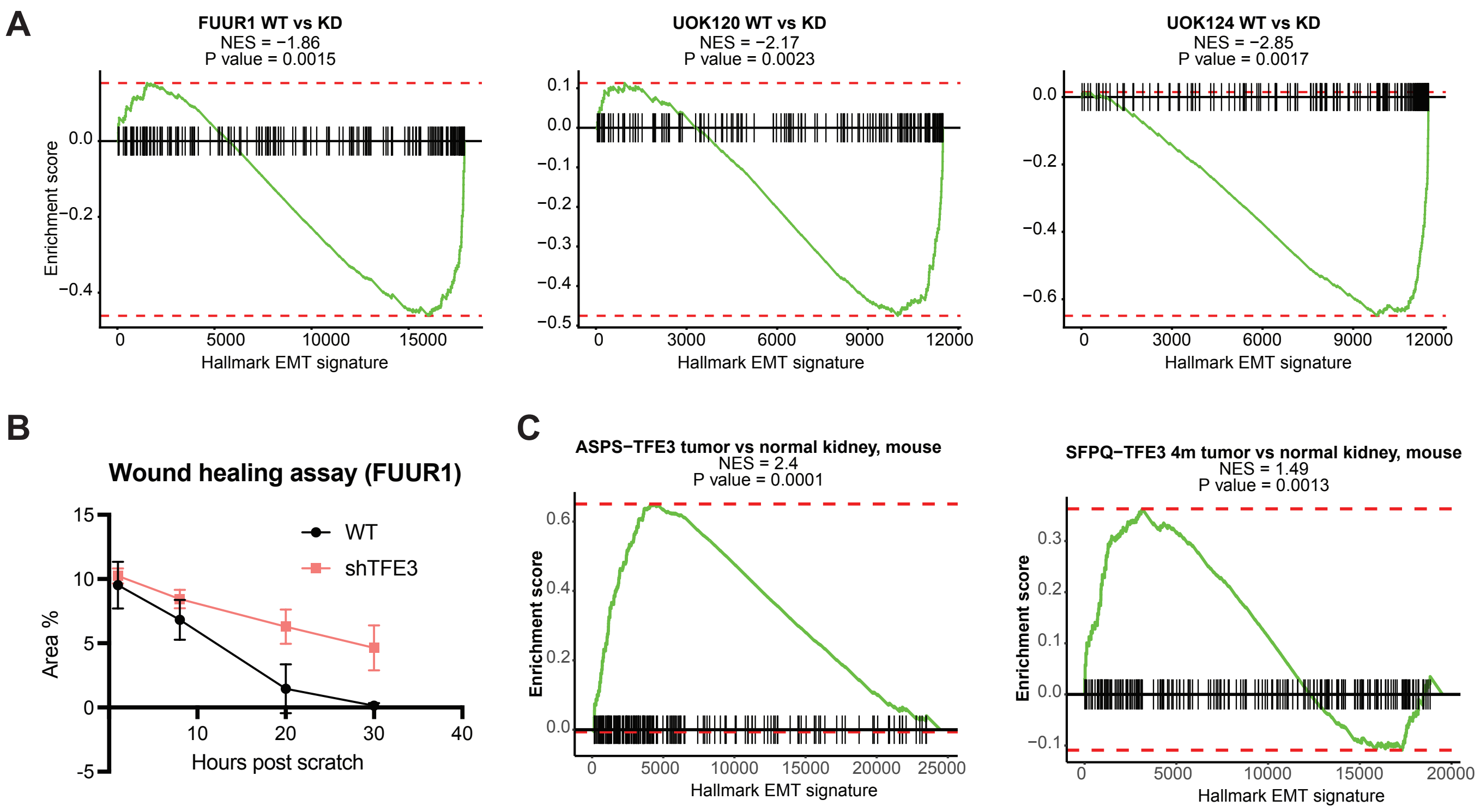

A
FUUR1 WT vs KD
NES = −1.86
P value = 0.0015
UOK120 WT vs KD
NES = −2.17
P value = 0.0023
UOK124 WT vs KD
NES = −2.85
P value = 0.0017
Enrichment score
Hallmark EMT signature
B
Wound healing assay (FUUR1)
WT
shTFE3
Area %
Hours post scratch
C
ASPS−TFE3 tumor vs normal kidney, mouse
NES = 2.4
P value = 0.0001
SFPQ−TFE3 4m tumor vs normal kidney, mouse
NES = 1.49
P value = 0.0013

Figure S5. Epithelial mesenchymal transition program in tRCC tumors

A. Gene set enrichment analysis (GSEA) plots showing the Hallmark epithelial-mesenchymal transition (EMT) gene signature upon TFE3 fusion knockdown in tRCC cell lines harboring ASPSCR1-TFE3 (FUUR1; NES = −1.86, *P* = 0.0015), PRCC-TFE3 (UOK120; NES = −2.17, *P* = 0.0023), or PRCC-TFE3 (UOK124; NES = −2.85, *P* = 0.0017) fusions. WT, wild-type; KD, knockdown.

B. Wound healing assay in FUUR1 cells expressing wild-type (WT) TFE3 fusion or TFE3 fusion knockdown (shTFE3), showing the percentage of open wound area remaining at 0, 6, 20, and 30 hours post-scratch. Data (N=6 replicates) represent mean ± SEM.

C. GSEA plots showing enrichment of the Hallmark EMT gene signature in murine models of ASPSCR1-TFE3[76] (NES = 2.4, *P* = 0.0001) and SFPQ-TFE3[77] (NES = 1.49, *P* = 0.0013) tumors compared to normal kidney tissue.

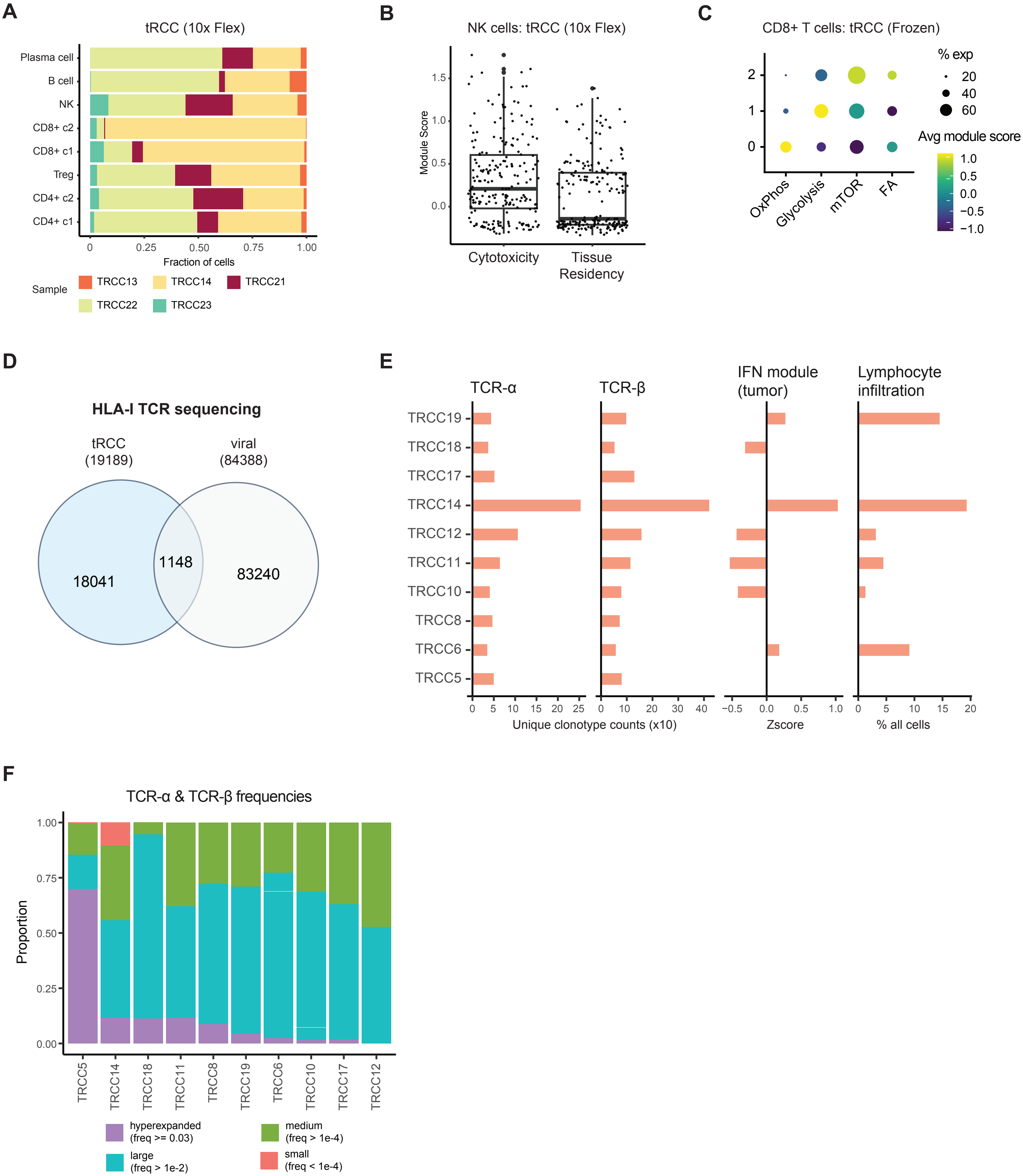
A
tRCC (10x Flex)
Plasma cell
B cell
NK
CD8+ c2
CD8+ c1
Treg
CD4+ c2
CD4+ c1
0
0.25
0.50
0.75
1.00
Fraction of cells
Sample
TRCC13
TRCC14
TRCC21
TRCC22
TRCC23
B
NK cells: tRCC (10x Flex)
Module Score
1.5
1.0
0.5
0.0
Cytotoxicity
Tissue Residency
C
CD8+ T cells: tRCC (Frozen)
2
1
0
OxPhos
Glycolysis
mTOR
FA
% exp
20
40
60
Avg module score
1.0
0.5
0.0
−0.5
−1.0
D
HLA-I TCR sequencing
tRCC (19189)
viral (84388)
18041
1148
83240
E
TCR-α
TCR-β
IFN module (tumor)
Lymphocyte infiltration
TRCC19
TRCC18
TRCC17
TRCC14
TRCC12
TRCC11
TRCC10
TRCC8
TRCC6
TRCC5
0 5 10 15 20 25
0 10 20 30 40
Unique clonotype counts (x10)
−0.5 0.0 0.5 1.0
Zscore
0 5 10 15 20
% all cells
F
TCR-α & TCR-β frequencies
Proportion
1.00
0.75
0.50
0.25
0.00
TRCC5
TRCC14
TRCC18
TRCC11
TRCC8
TRCC19
TRCC6
TRCC10
TRCC17
TRCC12
hyperexpanded (freq >= 0.03)
medium (freq > 1e-4)
large (freq > 1e-2)
small (freq < 1e-4)

Figure S6. Lymphocyte infiltration and T cell receptor (TCR) clonality in tRCC

A. Stacked bar plot showing the composition of lymphocytes in tRCC 10x Flex cohort.
B. Box plot of cytotoxicity and tissue residency module scores in NK cells from the tRCC 10x Flex cohort.
C. Dot plot of metabolic pathway module scores in CD8 T cell subclusters from the tRCC frozen cohort.
D. Venn plot showing intersection of TCR sequences from bulk-TCRα and TCRβ sequencing of tRCC samples and publicly annotated viral TCR sequences.
E. Bar plots of counts of unique TCRα and TCRβ clonotypes identified in each tRCC sample, corresponding interferon (IFN) module scores in tumor cells, and percentage lymphocyte infiltration from snRNA-seq data.
F. Proportions of TCR clonotypes in each tRCC sample based on their abundance: hyperexpanded, large, medium, and small. Proportions reflect values after removing overlapping viral sequences from D.

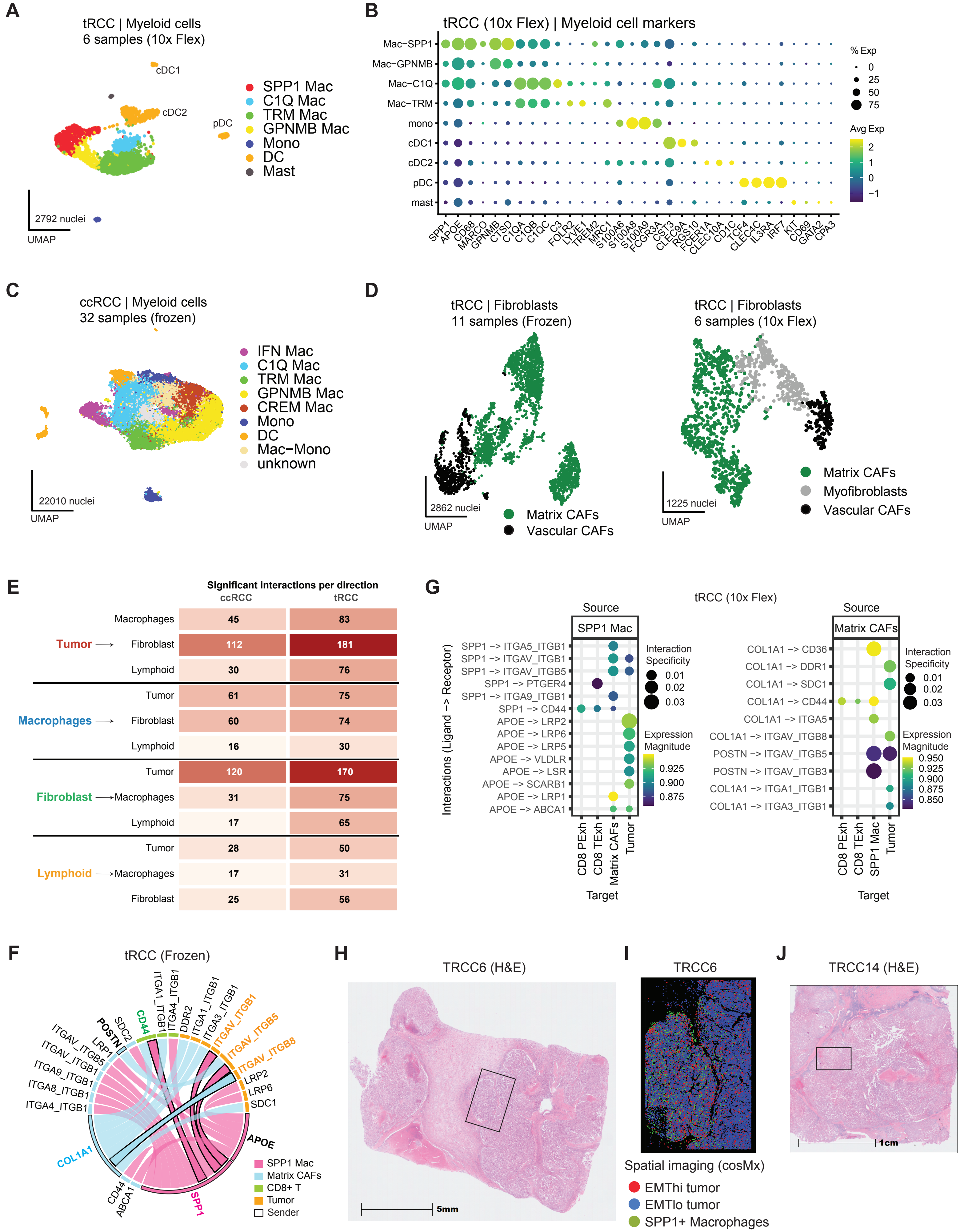

Source	Target	ccRCC	tRCC
Tumor →	Macrophages	45	83
	Fibroblast	112	181
	Lymphoid	30	76
Macrophages →	Tumor	61	75
	Fibroblast	60	74
	Lymphoid	16	30
Fibroblast →	Tumor	120	170
	Macrophages	31	75
	Lymphoid	17	65
Lymphoid →	Tumor	28	50
	Macrophages	17	31
	Fibroblast	25	56

Figure S7. Suppressive myeloid cells and fibroblasts in the tRCC TME

A. UMAP of myeloid cell subtypes in the tRCC (10x Flex) cohort.
B. Dot plot showing expression of marker genes in myeloid cell types in the tRCC 10x Flex cohort from A.
C. UMAP depicting myeloid cell subtypes identified in the ccRCC (frozen) cohort.
D. UMAPs depicting fibroblast subtypes identified in the tRCC frozen (left) & 10X flex (right) cohorts.
E. Heatmap depicting significant interactions between different cell types in tRCC and ccRCC frozen cohorts.
F. Chord diagram showing interactions of SPP1 macrophages and Matrix CAFs (senders) with tumor cells and CD8 T cells in the tRCC (frozen) cohort.
G. Dot plots highlighting interaction specificity and expression magnitude of interactions of SPP1 macrophages and Matrix CAFs (senders) with tumor cells and CD8 progenitor exhausted and terminal exhausted T cells in the tRCC (10X flex) cohort.
H. H&E staining of the full slide of sample TRCC6. The section highlighted indicates the field of view utilized for spatial imaging.
I. Spatial image plot from sample TRCC6 highlighting distribution of EMT-high tumor cells, EMT-low tumor cells, and SPP1+ macrophages.
J. H&E staining of full slide of sample TRCC14. The section highlighted indicates the field of view utilized for spatial imaging.
