# Supplementary material for: Fusion-driven oncogenic programs shape the immune landscape in translocation renal cell carcinoma
Source: Nat Commun. 2026 Aug 26;17:10191. doi: 10.1038/s41467-026-76858-w (PMC13612822; doi:10.1038/s41467-026-76858-w)
Supplement: Supplementary file 2 [file 41467_2026_76858_MOESM2_ESM.pdf]

Description of Additional Supplementary Files

Supplementary Data 1: Tab 1: Clinical characteristics of tRCC sequencing cohort. Related to Figure 1. Tab 2: Quality control metrics of snRNA-seq data of tRCC and ccRCC samples. Tab 3: Quality control metrics of snATAC-seq data of tRCC and ccRCC samples. Tab 4: Bulk RNA-seq datasets associated with tRCC and ccRCC used in this study. Tab 5: tRCC snRNA-seq dataset cell type marker genes. P value and adjusted P values identified by two-sided Wilxocon rank-sum test with Bonferroni correction.

Supplementary Data 2: Tab 1: tRCC-specific genes identified from snRNA-seq and bulk RNA-Seq. Related to Figure 2. Tab 2: Nephron marker genes associated with cell of origin analysis. P value and adjusted P values identified by two-sided Wilxocon rank-sum test with Bonferroni correction.

Supplementary Data 3: Tab 1: Genes included in each tRCC metaprogram. Tab 2: 113 gene fusion activity signature. Related to Figure 3.

Supplementary Data 4: TCRa and TCRb sequences from tRCC cohort profiled in this study. Related to Figure 4 and Figure S6.

Supplementary Data 5: Antibodies used for multiple immunofluorescence.
