# Supplementary material for: Fusion-driven oncogenic programs shape the immune landscape in translocation renal cell carcinoma
Source: Nat Commun. 2026 Aug 26;17:10191. doi: 10.1038/s41467-026-76858-w (PMC13612822; doi:10.1038/s41467-026-76858-w)
Supplement: Supplementary file 3 [file 41467_2026_76858_MOESM8_ESM.pdf]

nature portfolio

Corresponding author(s): Srinivas R Viswanathan & Toni K Choueiri

Last updated by author(s): Jul 16, 2026

Reporting Summary

Nature Portfolio wishes to improve the reproducibility of the work that we publish. This form provides structure for consistency and transparency in reporting. For further information on Nature Portfolio policies, see our Editorial Policies and the Editorial Policy Checklist.

Statistics

For all statistical analyses, confirm that the following items are present in the figure legend, table legend, main text, or Methods section.

n/a	Confirmed	
☐	☒	The exact sample size (*n*) for each experimental group/condition, given as a discrete number and unit of measurement
☒	☐	A statement on whether measurements were taken from distinct samples or whether the same sample was measured repeatedly
☐	☒	The statistical test(s) used AND whether they are one- or two-sided<br>*Only common tests should be described solely by name; describe more complex techniques in the Methods section.*
☐	☒	A description of all covariates tested
☐	☒	A description of any assumptions or corrections, such as tests of normality and adjustment for multiple comparisons
☐	☒	A full description of the statistical parameters including central tendency (e.g. means) or other basic estimates (e.g. regression coefficient) AND variation (e.g. standard deviation) or associated estimates of uncertainty (e.g. confidence intervals)
☐	☒	For null hypothesis testing, the test statistic (e.g. *F*, *t*, *r*) with confidence intervals, effect sizes, degrees of freedom and *P* value noted<br>*Give P values as exact values whenever suitable.*
☒	☐	For Bayesian analysis, information on the choice of priors and Markov chain Monte Carlo settings
☒	☐	For hierarchical and complex designs, identification of the appropriate level for tests and full reporting of outcomes
☐	☒	Estimates of effect sizes (e.g. Cohen's *d*, Pearson's *r*), indicating how they were calculated

Our web collection on statistics for biologists contains articles on many of the points above.

Software and code

Policy information about availability of computer code

Data collection	No software was used specifically for data collection in this study
Data analysis	All analyses described uses publicly available software as detailed in the relevant sub-sections of the Methods and Results section. No new computational tools or custom code/ algorithms were developed for this study.<br><br>R, version 4.1.2; 10x Genomics Cell Ranger, version 3.1.0; 10x Genomics Cell Ranger ATAC, version 2.0; Cell Ranger ARC human reference, refdata-cellranger-arc-GRCh38-2020-A-2.0.0; Seurat, version 5.4.0; Signac, version 1.12.0; Scrublet, version 0.2.3; SingleR, version 1.8.1; LIANA, version 0.1.14; CellPhoneDB, version 5; NMF R package, version 0.23.0; clusterProfiler, version 4.13.3; pySCENIC, version 0.12.1; JASPAR2020 motif database; RANN, version 2.6.1; STAR, version 2.7.10a; RSEM, version 1.3.1; GENCODE, release 45; TIMER2.0; survival R package, version 3.8-3; survminer, version 0.5.1; PhenoImager HT software, version 2.0.0; Phenochart, version 2.0.0; inForm, version 2.6.0; HALO, version 4.0.5107.407; and Fiji/ImageJ, version 2.16.0.

For manuscripts utilizing custom algorithms or software that are central to the research but not yet described in published literature, software must be made available to editors and reviewers. We strongly encourage code deposition in a community repository (e.g. GitHub). See the Nature Portfolio guidelines for submitting code & software for further information.

Data

Policy information about availability of data

All manuscripts must include a data availability statement. This statement should provide the following information, where applicable:

- Accession codes, unique identifiers, or web links for publicly available datasets
- A description of any restrictions on data availability
- For clinical datasets or third party data, please ensure that the statement adheres to our policy

The de-identified single-nucleus sequencing data generated in this study, including both raw and processed data, have been deposited in the NCBI Gene Expression Omnibus (GEO) under accession code GSE269466. T cell receptor sequencing data sis available within the paper in the supplementary information. Source data underlying the figures are provided with this paper in the accompanying source data files.
Previously generated RNA-sequencing and chromatin immunoprecipitation sequencing data from tRCC cell line models are publicly available in GEO under accession codes GSE266517 and GSE266530, respectively. The previously published HEK293T cell line dataset is available under accession code GSE188885. The dataset reported by Sun et al22 is available in GEO under accession code GSE167573. Data from The Cancer Genome Atlas (TCGA) Pan-Kidney cohort were obtained through the National Cancer Institute Genomic Data Commons35. Data from the IMmotion151 cohort were obtained from Motzer et al36, and data from the Wang et al37 cohort were obtained from the corresponding published study. The RNA-sequencing data reported by Qu et al23 are available through the National Omics Data Encyclopedia under project identifier OEP002630.

Research involving human participants, their data, or biological material

Policy information about studies with human participants or human data. See also policy information about sex, gender (identity/presentation), and sexual orientation and race, ethnicity and racism.

Reporting on sex and gender	Sex was reported based on patient self-report. Sex or Gender were not prospectively considered in the study design.
Reporting on race, ethnicity, or other socially relevant groupings	Race and/ ethnicity information is not collected in this study.
Population characteristics	Treatment categories are included in Supplementary Data 1.
Recruitment	N/A
Ethics oversight	Samples were collected with informed consent in concordance with Institutional Review Board (IRB) approval at the Dana-Farber/Harvard Cancer Center.

Note that full information on the approval of the study protocol must also be provided in the manuscript.

Field-specific reporting

Please select the one below that is the best fit for your research. If you are not sure, read the appropriate sections before making your selection.

☒ Life sciences ☐ Behavioural & social sciences ☐ Ecological, evolutionary & environmental sciences

For a reference copy of the document with all sections, see nature.com/documents/nr-reporting-summary-flat.pdf

Life sciences study design

All studies must disclose on these points even when the disclosure is negative.

Sample size	Sample sizes were chosen based on the availability of samples. No specific statistical methods were used to predetermine sample size. For bulk RNAseq and single-nucleus sequencing of ccRCC samples, we collected samples from published datasets, as indicated in the methods.
Data exclusions	Quality control was applied to exclude cells with low quality. Detailed methods are described in the Methods section. Datasets used for specific analysis are indicated in the study. No additional data exclusions related to samples were considered.
Replication	The study incorporates multiple co-horts, including single-nucleus and bulk RNA seq cohorts, and where applicable, results were validated/ replicated in these cohorts. We also validated multiple findings using in vitro experimentation and IHC on ad-ditional tumor samples.
Randomization	The current work was not an experimental study so did not require randomisation into experimental groups.
Blinding	The current work was not an experimental study so did not require blinding to group allocation during data collection or analysis.

Reporting for specific materials, systems and methods

We require information from authors about some types of materials, experimental systems and methods used in many studies. Here, indicate whether each material, system or method listed is relevant to your study. If you are not sure if a list item applies to your research, read the appropriate section before selecting a response.

Materials & experimental systems

n/a	Involved in the study
☐	☒ Antibodies
☐	☒ Eukaryotic cell lines
☒	☐ Palaeontology and archaeology
☒	☐ Animals and other organisms
☒	☐ Clinical data
☒	☐ Dual use research of concern
☒	☐ Plants

Methods

n/a	Involved in the study
☒	☐ ChIP-seq
☒	☐ Flow cytometry
☒	☐ MRI-based neuroimaging

Antibodies

Antibodies used	The following primary antibodies were used for multiplex immunofluorescence staining on the BOND RX automated stainer (Leica Biosystems): rabbit anti-SPP1 (clone EPR21139-316; Abcam, Cat# ab214050, RRID: AB_2894860; 1:1500), rabbit anti-CD86 (clone E2G8P; Cell Signaling Technology, Cat# 91882, RRID: AB_2797422; 1:100), rabbit anti-MRC1 (clone E2L9N; Abcam, Cat# 91992, RRID: AB_2800175; 1:400), mouse anti-CD8 (clone C8/144B; Agilent, Cat# M7103, RRID: AB_2075537; 1:1500), mouse anti-PD-1 (clone EH33; gift from Dr. Gordon Freeman, PMID: 16819321; 1:2500), and rabbit anti-CD163 (clone 10D6; Leica Biosystems, Cat# NCL-L-CD163, RRID: AB_2756375; 1:200). All antibodies were diluted in BOND Primary Antibody Diluent (Leica Biosystems, Cat# AR9352), with the exception of anti-PD-1, which was diluted in Da Vinci Green Diluent (BioCare Medical, Cat# PD900 M). Mouse primary antibodies (anti-CD8 and anti-PD-1) required a Post Primary Leica reagent prior to secondary detection. Signal detection was performed using the BOND Polymer Refine Detection Kit (Leica Biosystems) with tyramide signal amplification (TSA), employing the following Opal fluorochromes (Akoya Biosciences): Opal 520 (1:100) for SPP1, Opal 570 (1:150) for CD86, Opal 620 (1:100) for MRC1, Opal 480 (1:100) for CD8, Opal 690 (1:100) for PD-1, and Opal 780 (1:25) with TSA Plus DIG (1:100) for CD163.
Validation	All primary antibodies were validated by the respective commercial suppliers. Validation documentation, including specificity testing and reactivity data, is available through the manufacturer's product pages referenced by the catalogue numbers and Research Resource Identifiers (RRIDs) provided above.

Eukaryotic cell lines

Policy information about cell lines and Sex and Gender in Research

Cell line source(s)	The human tRCC cell line FU-UR-1 was obtained from the laboratory of M. Ishiguro at Fukuoka University School of Medicine.
Authentication	STR profiling was performed for cell line authentication.
Mycoplasma contamination	All cell lines were tested negative for mycoplasma contamination
Commonly misidentified lines (See ICLAC register)	No commonly misidentified lines were used

Plants

Seed stocks	NA
Novel plant genotypes	NA
Authentication	NA
