# Supplementary material for: Fusion-driven oncogenic programs shape the immune landscape in translocation renal cell carcinoma
Source: Nat Commun. 2026 Aug 26;17:10191. doi: 10.1038/s41467-026-76858-w (PMC13612822; doi:10.1038/s41467-026-76858-w)
Supplement: Supplementary file 4 [file 41467_2026_76858_MOESM9_ESM.pdf]

nature portfolio

Peer Review File

Fusion-driven oncogenic programs shape the immune landscape in translocation renal cell carcinoma

Corresponding Author: Dr Srinivas Viswanathan

This file contains all reviewer reports in order by version, followed by all author rebuttals in order by version.

Version 0:

Reviewer comments:

Reviewer #1

(Remarks to the Author)
This is an important and timely study by Konda, Weiss, Cui, Viswanathan, and colleagues, who profiled a cohort of 20 tRCC samples using single-nucleus RNA sequencing (snRNA-seq), single-nucleus ATAC sequencing (snATAC-seq), spatial transcriptomics, and T cell receptor (TCR) sequencing. Although not all cases were analyzed on every platform, the findings provide valuable insights into the biology of tRCC. The authors also integrated bulk transcriptomic data from 122 tRCC samples with 1,300 ccRCC bulk and 32 ccRCC single-cell transcriptomic profiles, revealing notable differences in TME composition between tRCC and ccRCC. These findings may help explain differences in immunotherapy response between RCC subtypes and inform therapeutic strategies for this rare tumor type.
Comments to be addressed are listed below:
1. Please add additional columns supplementary table-1 to provide some sample-wise QC metrics for each platform. These are essential for assessing data quality and will facilitate future integrative analyses. It would also be informative to include similar QC metrics for the “32 snRNA-seq and 24 snATAC-seq ccRCC samples” that were reprocessed by the authors. Additionally, a master metadata table summarizing all 128 tRCC and 1,311 ccRCC bulk transcriptome samples would provide a one-stop reference for independent future analyses.
2. The following sentence requires clarification:“Given the rarity of tRCC, the cohort was heterogeneous with respect to tumor site (11 primary, 5 metastases) and treatment history (8 treatment naïve, 8 with prior systemic therapy) (Figure S1A & Table S1).”
Based on Figure S1A, there appear to be 8 treated and 10 untreated samples across the cohort, and 7 treated samples among those profiled by snRNA-seq. Also, since one patient contributed multiple sites, counting tumor sites individually in this statement may be misleading. Please revise accordingly.
3. The statement “While frozen and FFPE snRNA-seq platforms yielded relatively comparable feature distributions, cell type proportions differed between platforms” would be strengthened by a direct comparison for TRCC-14, the case profiled using both methods.
4. For the TCGA data (S.Fig 1F), please clarify whether the RCC sample annotation from prior pan-RCC studies—Chen & Creighton et al. (Cell Rep 2016; PMID: 26947078) or Ricketts et al. (PMID: 29617669)—was used. These studies reclassified and curated several RCC samples and defined ccRCC subtypes (CC-e1 to CC-e3).
o The misclustering of ccRCC and chRCC in Fig. 1D suggests possible annotation issues.
o The immune deconvolution in Fig. 1E may also be misleading, as chRCC is generally immune cold, while ccRCC comprises immune-rich, immune-desert, and endothelial-rich subtypes.
o Incorporating Chen et al.’s classification would likely refine these comparisons.
5. The statement “Immune deconvolution analyses (CIBERSORTx) inferred increased TAM in tRCC compared to both ccRCC and chRCC, as well as reduced CD8+ T cells in tRCC relative to ccRCC” may be misleading, since the difference between tRCC and ccRCC is not statistically significant.
o Reanalyzing this using Chen et al.’s ccRCC subtypes may reveal more nuanced patterns.
o It would also be valuable to validate these findings in the snRNA-seq dataset (compare tRCC with Ref 35) to support the claim.
6. Please provide tables listing top marker genes for each cell type identified in the snRNA-seq dataset (this study) and for the public datasets used in Fig. S2F, ideally as supplementary tables.
7. A more detailed discussion of PEC populations is warranted. Are the cells labeled as PEC in this dataset restricted to the Bowman’s capsule lining, or do they include rare PEC-like populations within PT?
o Zhang et al. (Ref 51) described PT-A and rare PT-B (ITGB8+, VCAM+) and PT-C (CFH+, VCAM+) populations.

o Lake et al. (PMID: 31249312) identified similar clusters (PT-4 and cluster 29).
o Given that PEC is predicted as a potential cell of origin for a subset of tRCC cases in this study, placing these findings in the context of prior PT/PEC annotations would strengthen the manuscript.
8. Typographical error: Line 195: “MI67” should be corrected to “MKI67.”
9. Endothelial fraction and malignant cell angiogenesis metaprogram, In Fig. 1B, TRCC6 and TRCC11 show high endothelial cell fractions, while 18Li2, Li4, and RPLN do not. However, the text (line 206 onwards) associates angiogenic metaprograms with all these samples.
o Please clarify whether angiogenic metaprogram expression correlates with endothelial fraction or histologic vascularity.
10. Including ligand–receptor cell–cell communication analysis for ccRCC (using ccRCC snRNAseq) alongside the tRCC results in Fig. 5D would help highlight key differences between tumor types.
11. The statement “Despite the genomic homogeneity of tRCC... we observed significant transcriptional heterogeneity both within and among tumors” raises the question of whether subclonal CNV differences contribute to this heterogeneity. Consider inferCNV analysis or similar approaches to explore this.
12. Thank you for providing reviewer access to the GEO data. The GSE307120 entry appears incomplete:
o The 10X Flex data are not listed.
o A sample metadata table linking GEO samples to Supplementary Table-1 is missing, and the run selector link is empty.
o If possible, please also include count matrices to enable downstream reanalysis by future users.

Reviewer #2

(Remarks to the Author)
In this manuscript, Konda et al., present a comprehensive multi-omic study of tRCC. They provide snRNA-seq, snATAC-seq, spatial transcriptomics, and TCR-seq and contextualize the findings relative to ccRCC and pan-cancer datasets. The study has an impressive breath, but there appears to be a heightened focus on revalidating prior findings and there is a missed opportunity for discovery. In addition, most findings are correlative in nature.

Major Comments.
The cohort size is modest (15 tRCC cases) and highly heterogeneous. As shown in the t-SNE plots of Figure 1 while malignant cells from different samples (primary and metastatic) from the same patient (e.g., TRCC18) cluster together, samples harboring the same fusion (e.g., PRCC–TFE3: TRCC11, TRCC12, TRCC19) were broadly dispersed. Authors should clarify how this may confound the interpretation of the results.

A more systematic approach (e.g., enrichment scores, adjusted p-values/FDR, effect sizes) for all pathway enrichment analyses (Figure 2) should be provided beyond selected pathways.

An explanation is needed for why the proximal tubule (PT) and EMT signatures appear mutually exclusive between primary and metastatic samples (Figure S3).

The snATAC–ChIP integration studies should be extended evaluating enrichment at pathway/ gene set levels (e.g. EMT).

While limited by numbers, it would be interesting to assess whether EMT, SPP1, and mCAF signatures predict survival independently of fusion partner type.

It would be good to expand on why the TME differs between ASPS and other fusions and how it may affect ICI responsiveness.

The immune deconvolution analyses indicating macrophage enrichment and a reduction in CD8 T cells should be validated by IHC ideally in a larger cohort.

The link between TFE3 fusion and EMT is intriguing but remains correlative. Functional validation such as knockdown/rescue experiments or CRISPR-based perturbations would be important to strengthen the causal relationship between TFE3 fusion and EMT induction.

Discovery of $SPP1^+$ macrophages and mCAFs as suppressive hubs is novel. While spatial data confirmed proximity, functional validation would greatly strengthen the therapeutic implications, but understandably, this may be beyond the scope of this paper.

Minor comments:
Please clarify the methodology used to process and integrate snRNA-seq data from fresh frozen (3′ snRNA-seq and 3′ Multiome) and FFPE samples. Since multiple platforms were combined, how were batch effects identified, addressed, and corrected? Providing additional QC metrics would further strengthen confidence in the integration and interpretation of these data.

Please provide the specific gene sets used to calculate the EMT score in Figure 3 and explain why COL1A1, SPP1, or VCAN (top-ranked in the NFM EMT signature) are not among the 113 fusion-regulated genes (Figure 3C).

Did the authors account for the 15 TCGA tRCC cases that were not annotated or subclassified? If so, were these excluded prior to analysis?

Reviewer #3

(Remarks to the Author)
I co-reviewed this manuscript with one of the reviewers who provided the listed reports. This is part of the Nature Communications initiative to facilitate training in peer review and to provide appropriate recognition for Early Career Researchers who co-review manuscripts.

Reviewer #4

(Remarks to the Author)
I co-reviewed this manuscript with one of the reviewers who provided the listed reports. This is part of the Nature Communications initiative to facilitate training in peer review and to provide appropriate recognition for Early Career Researchers who co-review manuscripts.

Reviewer #5

(Remarks to the Author)
In this manuscript, Konda et al describe a comprehensive analysis of single-cell transcriptomes and chromatin accessibility profiles from translocation renal cell carcinoma (tRCC), an aggressive RCC defined by oncogenic TFE3 gene fusions. The authors assembled a cohort of 20 tRCC samples which were profiled using single-nucleus RNA sequencing (snRNA-seq), along with single nucleus ATAC sequencing (snATAC-seq), spatial transcriptomic analysis, and T Cell Receptor (TCR) sequencing. By integrating 128 bulk transcriptomic tRCC profiles from prior studies and a systematic comparison with 1300 published ccRCC bulk and 32 single-cell transcriptional profiles, authors present a detailed picture of how TFE3 fusions drive malignancy and contribute to immunotherapy resistance.

This is an important contribution to understanding the biology of an understudied cancer type. There are several issues that should first be addressed.

MAJOR COMMENTS:
- For the analysis in Figure 1E, a claim is made that there are more macrophages in tRCC, upon immune deconvolution. This claim could use further support. First, what was counted as macrophage from CIBERSORTx (assuming LM22 matrix was used)? M2? M1?. Second, it looks like the relative abundance of immune populations were shown, but we know there are dramatic differences in immune infiltration between tumor types. It would be helpful to examine the differences using "absolute" mode for CIBERSORTx. Lastly, there is no statistical comparison done – it would be more convincing if a statistical test were performed showing that there is significantly higher macrophages levels in tRCC compared to the other tumor types.

- In figure 3, the authors chose to separately analyze the snRNAseq from frozen and FFPE, which is reasonable. However, for the tumor metaprogram analysis, it is unclear whether this is found in the snRNA-seq from frozen, from FFPE, or from both. The authors should clarify this, and if only performed in one (e.g. frozen), it would be helpful to validate in the other.

- For figure 4, it would be helpful to gain some confidence around the claim that CD8 c1 is a progenitor exhausted cluster, vs simply a non-exhausted population. Does this cluster express intermediate levels of PD1? Any TOX expression? It is very possible that c1 is simply a non-exhausted CD8 T cell population.

- For the analysis in 4E, again here to make the claim that a certain population is expanded, a statistical comparison should be done on a per sample basis (e.g. the proportion of CD8 c1 in each sample with ICI compared to each sample without ICI).

- For the spatial analysis in figures 5F and 5G, it would be helpful to provide some quantification / summary statistics to confirm the claim of the receptor-ligand interactions.

MINOR COMMENTS:
- The total number of cells (post QC) for each dataset (snRNASeq) can be mentioned in the main text or in a supplemental table
- A note on how Harmony variables were defined (e.g. by sample, batch or patient) could be included.

Reviewer #6

(Remarks to the Author)
Reviewer Comments
The authors investigate tRCC, a relatively rare renal tumor, using a multimodal approach that includes snRNA-seq, snATAC-seq, TCR-seq, and spatial transcriptomic analyses to elucidate its cell of origin and to clarify, from the perspective of TME, why responses to ICIs tend to be poor. To the best of my knowledge, there have been no prior tRCC studies utilizing snRNA-seq and snATAC-seq, and the study design is interesting. The authors also perform analyses using publicly available datasets. However, considering the limited number of in-house cases, especially in light of pre- and post-chemotherapy sampling and the heterogeneity of fusion partners, the robustness of the conclusions may be insufficient.

Furthermore, the proposed formation of a suppressive TME involving progenitor-exhausted $CD8^{+}$ T cells, anti-inflammatory $SPP1^{+}$ macrophages, and mCAFs lacks immunohistochemical validation, and the cases subjected to spatial transcriptomic analysis may not appear representative of tRCC in this study.

1. Compared with other cohorts (PMID: 40057038), many of the cases in your study are at an advanced stage. This tendency appears to be the same even among cases that did not receive chemotherapy. I would like to know whether you intentionally selected advanced-stage cases when choosing the samples, or whether the samples were selected randomly.

2. In Figure 2, the cases that underwent snRNA-seq should be clearly indicated. Given the small size of the cohort and the inclusion of both pre- and post-chemotherapy samples, specifying which cases were analyzed by snRNA-seq is crucial for assessing the validity of the study's results. If my understanding is correct that these are scRNA-seq cases that were not processed using 10x Flex, more than half of them appear to be post-chemotherapy cases. Since chemotherapy is known to affect both tumor cells and the immune microenvironment, it would be important to consider and discuss whether it may have influenced your results.

3. Many of the genes used for the EMT signal appear to be CAF markers, so I wonder whether this can appropriately be referred to as a "tumor EMT signature." How were the genes for the EMT signal in Figure 3 selected? As key EMT markers are not included, I believe you should consider adding and analyzing representative EMT-related genes such as VIM, CDH2, S100A4, ITGA5, ITGB1, COL5A1, SNAI1/2, ZEB1/2, and TWIST1.

4. I would like to know why you chose to exclude typical PT markers such as LRP2 and AQP1.

5. In Figure 4E, the comparison between ICI and non-ICI cases is based on only a single ICI-treated case, so the conclusions that can be drawn from these data appear to be weak. In addition, while Figure 4E suggests that the CD8+c1 subtype is more frequent in the ICI group, Figure 4F shows that $CD8^{+}$ T cells separate cleanly into ICI and non-ICI groups, which is difficult to interpret.

6. In ccRCC, $SPP1^{+}$ macrophage infiltration has been reported (PMID: 36423636), and these cells have been implicated in $CD8^{+}$ T-cell dysfuncton(PMID: 40680256). I believe it would be appropriate to explicitly acknowledge their role in the immunosuppressive microenvironment of ccRCC and then discuss tRCC in that context. In addition, how do you explain the absence of $SPP1^{+}$ macrophages in ccRCC in Figure 5B?

7. The cases shown in Figures 5E and 5F are post-chemotherapy, and I'm not sure whether a case treated with an mTOR inhibitor can be a representative example. The case in Figure 5G also harbors a LUC7L3 fusion, which is a very rare subtype even among tRCC. In light of these points, can the findings derived from these cases truly be generalized to tRCC as a whole? In addition, $SPP1^{+}$ macrophages appear to be quite abundant; however, histologically it is not generally recognized that tRCC is characterized by prominent macrophage infiltration. Moreover, $SPP1^{+}$ macrophages themselves seem to represent only a relatively small subset. Could you demonstrate the colocalization of SPP1 with macrophage markers by multiplex staining, for example using CD68 or other macrophage markers in the other cases?

8. Please correct the label in Supplementary Figure 3B from "Primary tumor nephrectomy" to "Primary tumor nephrectomy."

9. For case TRCC22, the stage is listed as IV, while the pathological status is given as pT3N0, and there is no description of distant metastasis. It would be better to clarify whether distant metastasis was present (Stage IV) or whether the correct stage is Stage III.

10. In the Results section, within the subsection "Suppressive macrophages and fibroblasts in the tRCC TME," there is a sentence that ends with a comma instead of a period. Please correct this.

11. Please check the spelling of "temsirolimus" in Supplementary Table 1.

Version 1:

Reviewer comments:

Reviewer #1

(Remarks to the Author)
The authors have fully addressed all my comments and questions, and I recommend the manuscript for further consideration for publication.

Reviewer #2

(Remarks to the Author)
The authors have addressed my comments and I support publication.

Reviewer #3

(Remarks to the Author)
I co-reviewed this manuscript with one of the reviewers who provided the listed reports. This is part of the Nature Communications initiative to facilitate training in peer review and to provide appropriate recognition for Early Career Researchers who co-review manuscripts.

Reviewer #4

(Remarks to the Author)
I co-reviewed this manuscript with one of the reviewers who provided the listed reports. This is part of the Nature Communications initiative to facilitate training in peer review and to provide appropriate recognition for Early Career Researchers who co-review manuscripts.

Reviewer #5

(Remarks to the Author)
The authors have fully addressed my comments

Reviewer #6

(Remarks to the Author)
The revisions appropriately address the reviewers' comments. I have no further comments.

Open Access This Peer Review File is licensed under a Creative Commons Attribution 4.0 International License, which permits use, sharing, adaptation, distribution and reproduction in any medium or format, as long as you give appropriate credit to the original author(s) and the source, provide a link to the Creative Commons license, and indicate if changes were made.
In cases where reviewers are anonymous, credit should be given to 'Anonymous Referee' and the source.
The images or other third party material in this Peer Review File are included in the article's Creative Commons license, unless indicated otherwise in a credit line to the material. If material is not included in the article's Creative Commons license and your intended use is not permitted by statutory regulation or exceeds the permitted use, you will need to obtain permission directly from the copyright holder.
To view a copy of this license, visit https://creativecommons.org/licenses/by/4.0/

RESPONSE TO REVIEWER COMMENTS

Reviewer #1 (Remarks to the Author)

This is an important and timely study by Konda, Weiss, Cui, Viswanathan, and colleagues, who profiled a cohort of 20 tRCC samples using single-nucleus RNA sequencing (snRNA-seq), single-nucleus ATAC sequencing (snATAC-seq), spatial transcriptomics, and T cell receptor (TCR) sequencing. Although not all cases were analyzed on every platform, the findings provide valuable insights into the biology of tRCC. The authors also integrated bulk transcriptomic data from 122 tRCC samples with 1,300 ccRCC bulk and 32 ccRCC single-cell transcriptomic profiles, revealing notable differences in TME composition between tRCC and ccRCC. These findings may help explain differences in immunotherapy response between RCC subtypes and inform therapeutic strategies for this rare tumor type.

We thank the reviewer for the encouraging assessment of our study. We appreciate the reviewer's recognition of the breath of our multi-omic profiling and analyses and the potential impact of these findings on understanding tRCC biology and therapeutic vulnerabilities. We have carefully revised the manuscript in response to the reviewer's insightful comments, and we hope the reviewer agrees that these revisions have strengthened our work.

Comments to be addressed are listed below:

1. Please add additional columns supplementary table-1 to provide some sample-wise QC metrics for each platform. These are essential for assessing data quality and will facilitate future integrative analyses. It would also be informative to include similar QC metrics for the "32 snRNA-seq and 24 snATAC-seq ccRCC samples" that were reprocessed by the authors. Additionally, a master metadata table summarizing all 128 tRCC and 1,311 ccRCC bulk transcriptome samples would provide a one-stop reference for independent future analyses.

We thank the reviewer for this suggestion. We have now included sample-wise QC metrics for each platform in Supplementary Table 1, including 18 tRCC snRNA-seq and 6 snATAC-seq samples, and the 32 snRNA-seq and 24 snATAC-seq ccRCC samples that were reprocessed in this study (sheets snRNA-seq_data_qc and snATAC-seq_data_qc in TableS1). We have also generated a comprehensive master metadata table summarizing all 128 tRCC and 1311 ccRCC bulk transcriptome samples (RNA-seq_datasets_used in TableS1). These additions should greatly facilitate future integrative analyses.

2. The following sentence requires clarification: "Given the rarity of tRCC, the cohort was heterogeneous with respect to tumor site (11 primary, 5 metastases) and treatment history (8 treatment naïve, 8 with prior systemic therapy) (Figure S1A & Table S1)."

- Based on Figure S1A, there appear to be 8 treated and 10 untreated samples across the cohort, and 7 treated samples among those profiled by snRNA-seq. Also, since one patient contributed multiple sites, counting tumor sites individually in this statement may be misleading. Please revise accordingly.

We thank the reviewer for pointing out this issue. The statement "Given the rarity of tRCC, the cohort was heterogeneous with respect to tumor site (11 primary, 5 metastases) and treatment history (8 treatment naïve, 8 with prior systemic therapy)" was intended to be in reference to the snRNA-seq cohort only. This includes 8 treatment naïve and 8 with prior systemic therapy, whereas the full cohort, including samples only used for TCR-sequencing include 8 treated and 10 untreated. As noted, one patient contributed multiple tumor sites. We have revised the manuscript text (lines 93-96) to reflect the above clarifications.

3. The statement "While frozen and FFPE snRNA-seq platforms yielded relatively comparable feature distributions, cell type proportions differed between platforms" would be strengthened by a direct comparison for TRCC-14, the case profiled using both methods.

We thank the reviewer for this suggestion. We have now included a direct, head-to-head comparison of TRCC-14 profiled using both frozen and FFPE snRNA-seq platforms. As shown in the Rebuttal Figure 1 (Figure S1E in revised manuscript) below, QC metrics such as doublet scores are comparable between platforms, while FFPE exhibits slightly higher nFeature_RNA than frozen tissue for this sample. Consistent with our original statement, cell type proportions differ between platforms, with greater tumor representation in the frozen samples and greater lymphoid representation in the FFPE samples, as supported by the head-to-head comparison for TRCC14. These additional graphs have been incorporated into the revised manuscript.

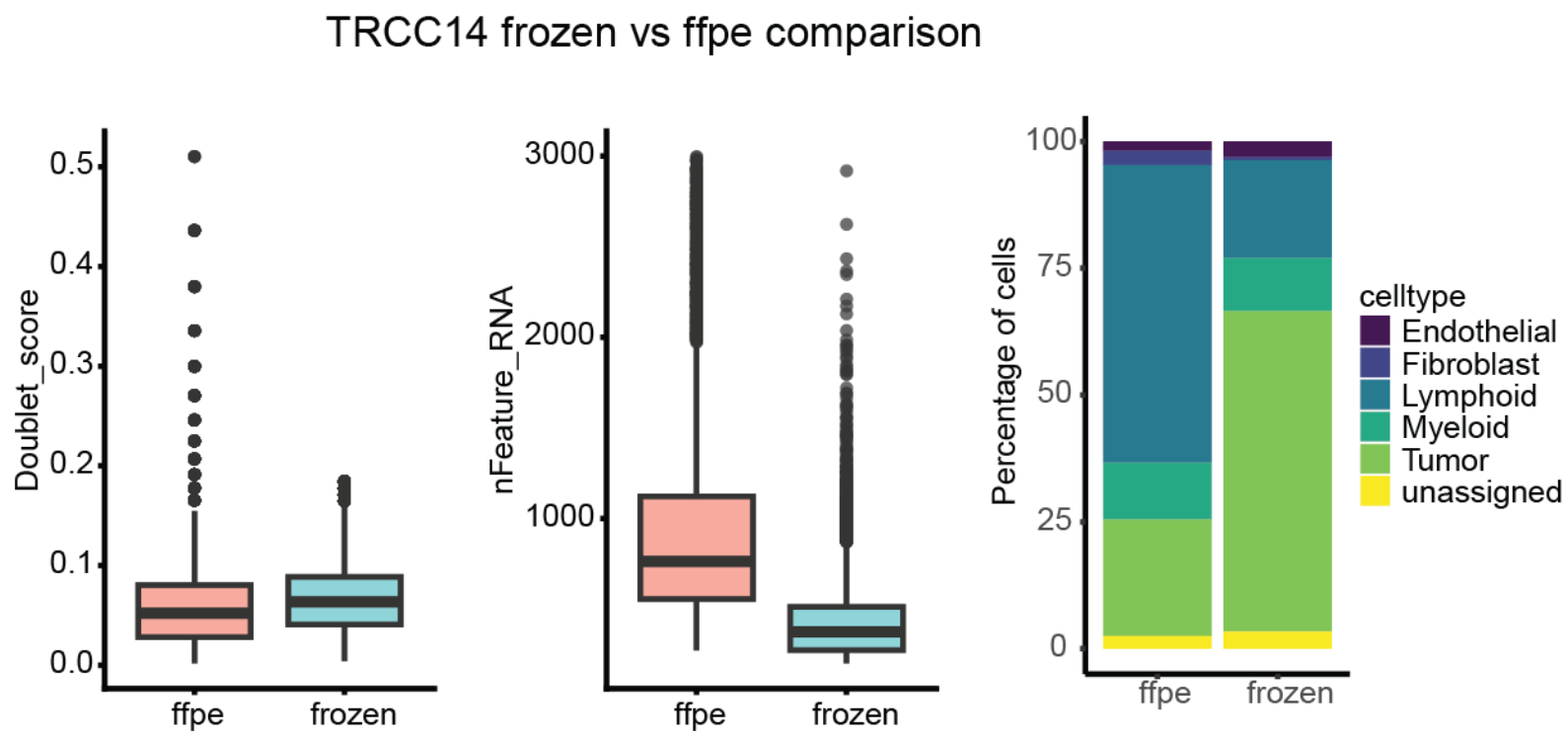

__Rebuttal Figure 1 (Fig. S1E in revised manuscript).__ *Comparison of TRCC14 profiled by frozen and FFPE snRNA-seq platforms. Boxplots show doublet scores (left), nFeature_RNA (middle), and stacked bar plot (right) shows the proportion of cell types identified in each platform, demonstrating differences in cell type proportions between frozen and FFPE preparations.*

4. For the TCGA data (S.Fig 1F), please clarify whether the RCC sample annotation from prior pan-RCC studies—Chen & Creighton et al. (Cell Rep 2016; PMID: 26947078) or Ricketts et al. (PMID: 29617669)—was used. These studies reclassified and curated several RCC samples and defined ccRCC subtypes (CC-e1 to CC-e3).

o The misclustering of ccRCC and chRCC in Fig. 1D suggests possible annotation issues.

o The immune deconvolution in Fig. 1E may also be misleading, as chRCC is generally immune cold, while ccRCC comprises immune-rich, immune-desert, and endothelial-rich subtypes.

o Incorporating Chen et al.'s classification would likely refine these comparisons.

We thank the reviewer for these important suggestions. As suggested, we have reannotated TCGA RCC samples using the Chen & Creighton et al.[1] classification, stratifying ccRCC into subtypes CC-e1/CC-e2/CC-e3. Following reannotation, ccRCC and chRCC cluster distinctly in the revised t-SNE representation (Rebuttal Figure 2A; Figure 1D in revised manuscript), resolving the prior misclustering. We have also updated the methods section to indicate the specific reannotation.

Regarding the immune deconvolution in Figure 1E, we agree with the reviewer that the original stacked barplot presentation was not ideal, as displaying cell type proportions within TME implied comparable immune infiltration across subtypes, which does not reflect the immune-cold nature of chRCC (as this reviewer rightly points out). We have therefore replaced our prior panel with a boxplot displaying immune cell estimates for macrophage and CD8+ T cell populations only, using the TIMER algorithm (which accounts for tumor purity) (Rebuttal Figure 2B; Figure 1E in revised manuscript). This revised presentation more accurately reflects the distinct immune landscapes across RCC subtypes for the cell types of focus in this manuscript, and is consistent with the known biology of chRCC as an immune-cold tumor[2]. We have updated the text (lines 117-129) to reflect these updates.

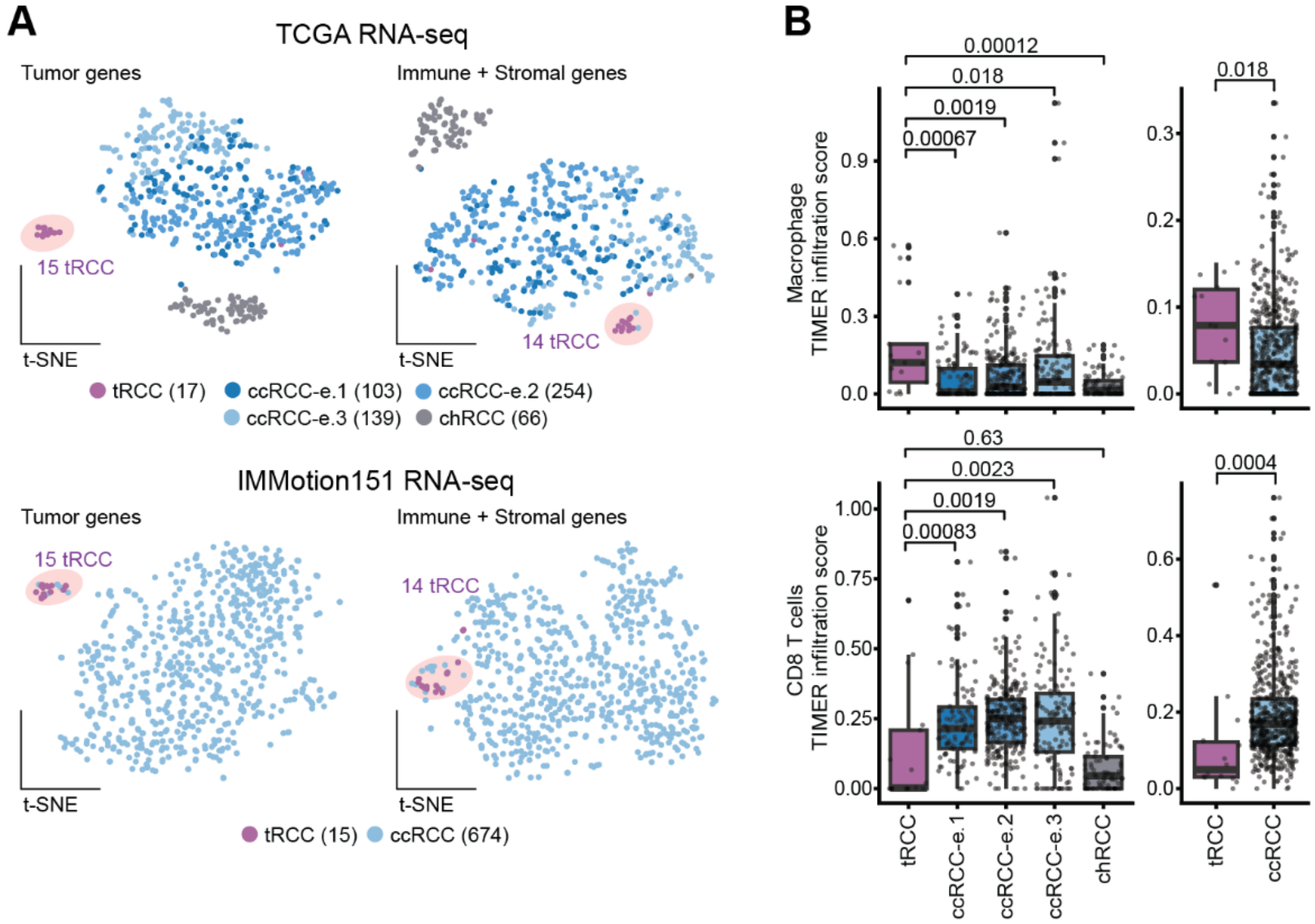

Rebuttal Figure 2. *t-SNE and immune deconvolution analyses following reannotation of TCGA RCC samples using the Chen & Creighton et al. (2016) classification. A. t-SNE plots of TCGA (top) and IMmotion151 (bottom) RNA-seq cohorts using tumor genes (left) and immune and stromal genes (right), with ccRCC stratified into subtypes CC-e1, CC-e2, and CC-e3.* **(Figure 1D in revised manuscript)** *B. Boxplots showing TIMER-based macrophage (top) and CD8+ T cell (bottom) infiltration scores across RCC subtypes in TCGA (left) and IMmotion151 (right) cohorts* **(Figure 1E in revised manuscript).** *P-values for indicated comparisons were calculated by Wilcoxon's rank-sum test.*

5. The statement "Immune deconvolution analyses (CIBERSORTx) inferred increased TAM in tRCC compared to both ccRCC and chRCC, as well as reduced CD8+ T cells in tRCC relative to ccRCC" may be misleading, since the difference between tRCC and ccRCC is not statistically significant.

o Reanalyzing this using Chen et al.'s ccRCC subtypes may reveal more nuanced patterns.

o It would also be valuable to validate these findings in the snRNA-seq dataset (compare tRCC with Ref 35) to support the claim.

We thank the reviewer for this careful evaluation. We have addressed this comment through several analytical and experimental updates.

First, following reannotation of TCGA samples using Chen et al.'s ccRCC subtype classifications, we reanalyzed immune deconvolution using TIMER[3], which accounts for tumor purity, as well as with CIBERSORTx[4] absolute mode. TIMER analysis demonstrates significantly higher macrophage infiltration in tRCC compared to all three ccRCC subtypes (c1/c2/c3) across both the TCGA and IMmotion151 cohorts (Rebuttal Figure 2B; Figure 1E in revised manuscript). For CD8+ T cells, the reduction in tRCC relative to ccRCC is statistically significant by both TIMER and CIBERSORTx absolute mode and is consistent across both bulk RNA-seq cohorts.

We acknowledge that TIMER and CIBERSORTx yield partially discordant results for macrophage subpopulations. TIMER infers higher overall macrophage abundance in tRCC, while CIBERSORTx absolute mode, which provides granular M0/M1/M2 estimates but does not account for tumor purity, suggests a higher M2/M1 ratio in tRCC relative to ccRCC, with a non-significant trend toward higher M0. Both graphs are presented in the revised manuscript (Rebuttal figures 2B and 3; Figures 1E and S2B-D in revised manuscript) with this interpretation made explicit in the text (lines 117-129). Overall, these findings are consistent with our higher-resolution snRNA-seq profiling of macrophage populations in tRCC vs. ccRCC, suggesting substantial qualitative and quantitative differences in this TME compartment between the two RCC subtypes.

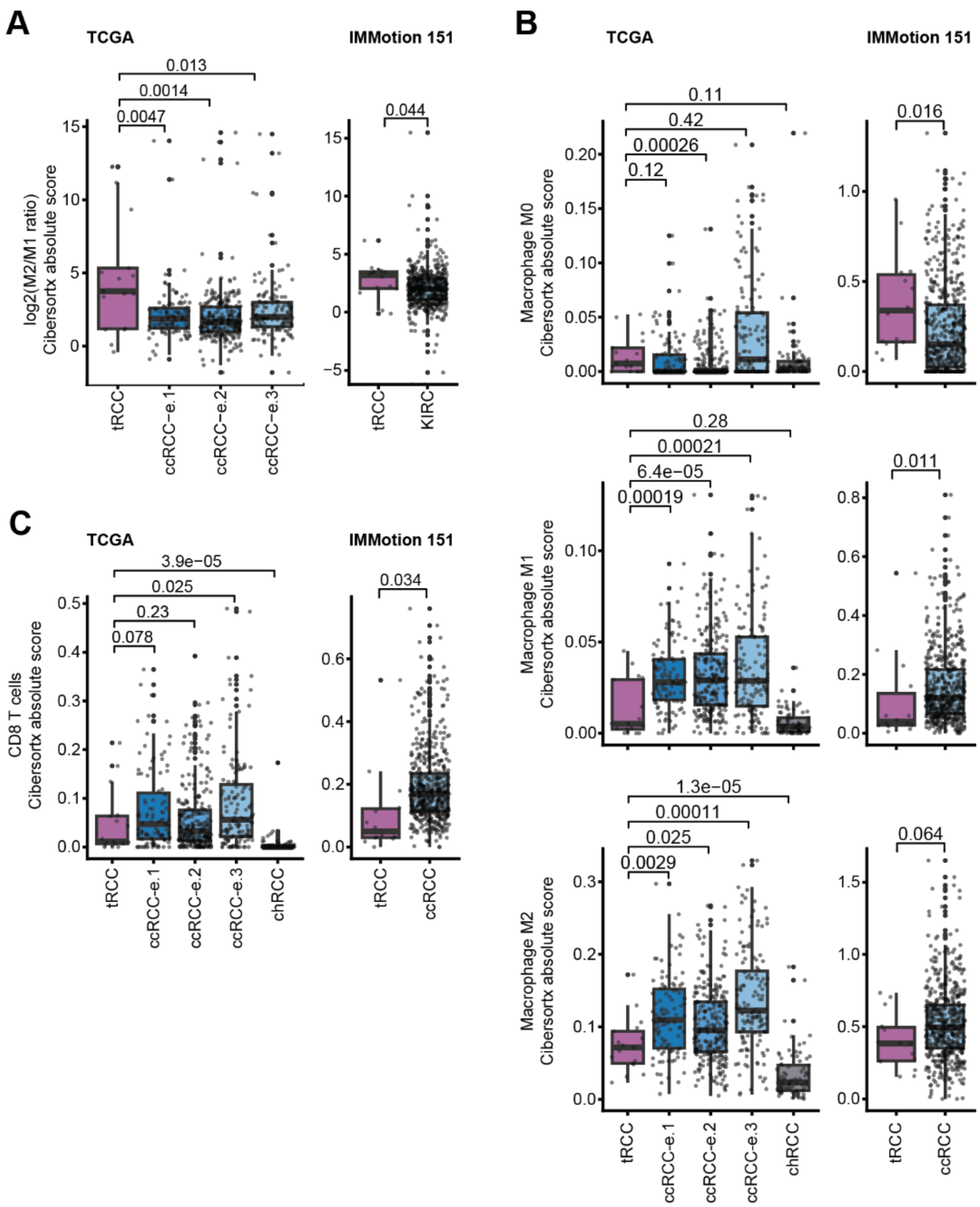

Rebuttal Figure 3. *CIBERSORTx absolute mode immune deconvolution of TCGA and IMmotion151 datasets. A. M2/M1 ratio **(Figure S2B in revised manuscript)**, B. macrophage subpopulations (M0, M1, M2) **(Figure S2C in revised manuscript)** and C. CD8+ T cells **(Figure S2D in revised manuscript)** across RCC subtypes in TCGA (left) and IMmotion151 (right) cohorts. tRCC shows a non-significant trend toward higher M0 macrophages relative to ccRCC subtypes, while M1 and M2 macrophages are lower in tRCC relative to ccRCC subtypes in the TCGA cohort. CD8+ T cells are lower in tRCC relative to ccRCC in both cohorts. P-values for indicated comparisons were calculated by Wilcoxon's rank-sum test.*

Finally, we have now validated the reduction in CD8+ T cell infiltration in tRCC using multiplex immunofluorescence (IF) on 10 tRCC cases (Rebuttal Figure 4; Figure 1F in revised manuscript), which demonstrates lower CD8+ and CD8+/PD1+ populations in tRCC relative to ccRCC (with the exception of one outlier tRCC case). This orthogonal validation strengthens our conclusion, complementing the comparative single-cell analysis with the published ccRCC cohort[5] already presented in the manuscript.

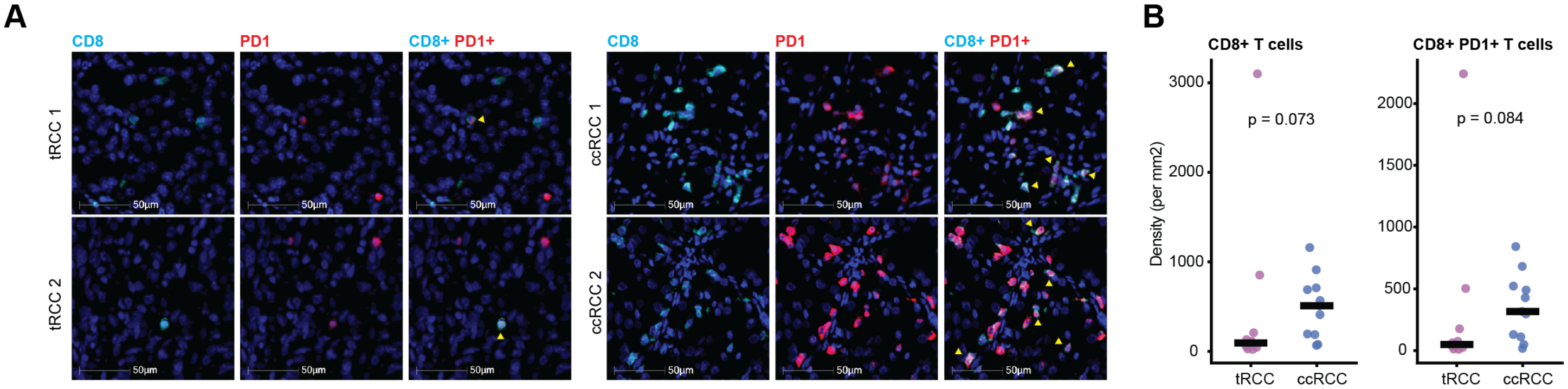

Rebuttal Figure 4 (Figure 1F in revised manuscript). *Multiplex immunofluorescence validation of CD8+ T cell infiltration in tRCC and ccRCC. A. Representative multiplex IF images showing CD8 (cyan) and PD1 (red) staining in two tRCC (left) and two ccRCC (right) cases. Yellow arrowheads indicate CD8+PD1+ double-positive cells. Scale bars, 50 µm. B. Quantification of CD8+ T cell density (left) and CD8+PD1+ T cell density (right) per* mm^2 *in tRCC (N=10) and ccRCC (N=11) cases. Horizontal bars indicate median values. P-values were calculated by Wilcoxon rank-sum test.*

Regarding direct comparison of macrophage abundance between tRCC and ccRCC at the snRNA-seq level using the Wu et al. dataset[5], in addition to baseline differences in sequencing metrics, we note that the published ccRCC snRNA-seq data underwent downsampling by the original authors in a manner that may preclude reliable quantitative comparison of absolute cell type proportions across datasets, as it is unclear whether cell type proportions were preserved during downsampling. For this reason, and to account for potential sample preparation differences with the Wu. et al. study, we have limited our analysis to proportional abundance of tumor programs and macrophage subsets in tRCC vs. ccRCC (as shown in Figs. 3 and 5).

Given these limitations, we believe that the TIMER-based bulk deconvolution analysis, which accounts for tumor purity and is applied uniformly across tumor types within a given study, and the multiplex immunofluorescence validation, represent more reliable orthogonal approaches for the tRCC:ccRCC comparison than cross-dataset snRNA-seq cell fraction comparisons. We have therefore relied on these approaches for the quantitative claims in the manuscript.

6. Please provide tables listing top marker genes for each cell type identified in the snRNA-seq dataset (this study) and for the public datasets used in Fig. S2F, ideally as supplementary tables.

We thank the reviewer for this suggestion. We have now included tables listing the top marker genes for each cell type identified in our snRNA-seq dataset, as well as for the public datasets used in Fig. S2F. These have been added to supplementary tables (sheet tRCC_snRNA_marker_genes in revised Table S1 and Nephorn_marker_genes in revised Table S2).

7. A more detailed discussion of PEC populations is warranted. Are the cells labeled as PEC in this dataset restricted to the Bowman's capsule lining, or do they include rare PEC-like populations within PT?

o Zhang et al. (Ref 51) described PT-A and rare PT-B (ITGB8+, VCAM+) and PT-C (CFH+, VCAM+) populations.

o Lake et al. (PMID: 31249312) identified similar clusters (PT-4 and cluster 29).

o Given that PEC is predicted as a potential cell of origin for a subset of tRCC cases in this study, placing these findings in the context of prior PT/PEC annotations would strengthen the manuscript.

We thank the reviewer for raising this important point and for suggesting contextualization with the reports by Zhang et al. and Lake et al. We believe it is most likely that the cluster annotated as PEC based on marker expression from our tumor-adjacent normal sample (TRCC19N) is not restricted to canonical Bowman's capsule-lining PECs but rather represents a transitional population at the PT/PEC interface, consistent with the rare progenitor populations described by Zhang et al.[6] and Lake et al.[7]

In the Rebuttal Figure 5A (Figure S3D in the revised manuscript), we have re-examined this cluster from TRCC19N using an expanded marker panel incorporating PT-B markers (*ITGB8*, *VCAM1*; Zhang et al.[6]), PT-C markers (*CFH*, *VCAM1*; Zhang et al.[6]), and Cluster 29 markers (*KCNT2*, *MGAT4C*, *CFH*, *ALDH1A2*; Lake et

al.[7]), alongside canonical PEC marker (*CLDN1*[8]). We show that, while this cluster expresses *CLDN1*, it also expresses multiple PT-like markers (but notably, no solute carrier genes), indicating overlap with both canonical PEC and the PT-B/PT-C/Cluster 29 populations described in the prior kidney atlases, and consistent with the well-recognized biological continuum[9–11] between Bowman's capsule-lining PECs and VCAM1-expressing proximal tubule progenitors. We have therefore reannotated this cluster as "PEC/PT" progenitor to reflect this transitional identity.

Notably, among the few tRCC cases (TRCC6, TRCC11, TRCC19) where PEC was the top predicted cell of origin, TRCC6 tumor cells showed detectable *CLDN1* expression most consistent with a more canonical PEC-like origin (Rebuttal Figure 5B), while other cases showed a more mixed PT-B/PT-C-like profile with overlapping marker gene expression, including VCAM1 expression. In our revised manuscript, we are cautious to classify these cases conservatively as arising from a VCAM1-expressing cell at the PT/PEC interface rather than from a strictly-defined canonical PEC, and have revised the results accordingly (lines 185-191 & 209-213). Overall, PT_VCAM1 remains the predominant predicted cell of origin across the majority of tRCC samples in both our single-cell and bulk RNA-seq cohorts, suggesting a common or, at the very least, highly similar, cell of origin between tRCC and ccRCC.

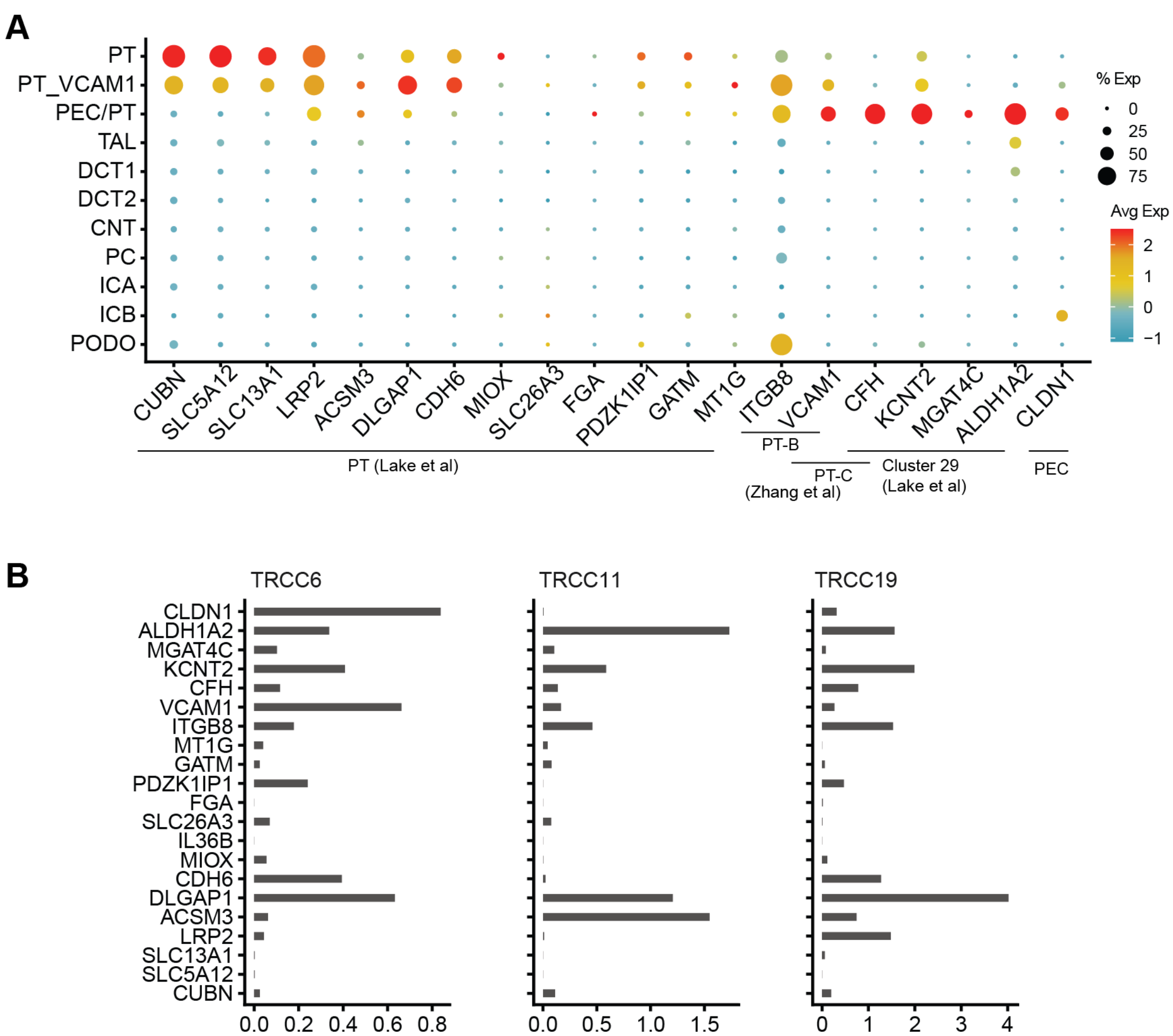

Rebuttal Figure 5. *Characterization of proximal tubule and parietal epithelial cell identity in tumor-adjacent normal kidney. A. Dot plot depicting expression of an expanded marker gene panel across normal kidney cell types identified in tumor-adjacent normal sample TRCC19N. Markers are grouped by reference population: PT markers from Lake et al. (CUBN, SLC5A12, SLC13A1, LRP2, ACSM3, DLGAP1, CDH6, MIOX, SLC26A3, FGA, PDZK1P1, GATM, MT1G); PT-B/PT-C markers from Zhang et al. (ITGB8, VCAM1, CFH); Cluster 29 markers from Lake et al. (KCNT2, MGAT4C, CFH, ALDH1A2); and canonical PEC marker (CLDN1)* ***(Figure S3D in revised manuscript).*** *B. Bar plots showing per-cell average expression of the marker gene panel in malignant cells from tRCC tumors assigned to PEC/PT progenitor group (TRCC6, TRCC11, TRCC19).*

8. Typographical error: Line 195: "MI67" should be corrected to "MKI67."

We thank the reviewer for catching this error. "MI67" has been corrected to "MKI67" in the revised manuscript.

9. Endothelial fraction and malignant cell angiogenesis metaprogram, In Fig. 1B, TRCC6 and TRCC11 show high endothelial cell fractions, while 18Li2, Li4, and RPLN do not. However, the text (line 206 onwards) associates angiogenic metaprograms with all these samples.

o Please clarify whether angiogenic metaprogram expression correlates with endothelial fraction or histologic vascularity.

We thank the reviewer for this important question. At the single-cell level, expression of the angiogenesis metaprogram in malignant cells does not clearly correlate with endothelial cell fraction across samples (Rebuttal Figure 6). We would also like to clarify that the tumor cells were assigned to metaprograms based on metaprogram with highest module score in the cell; the proportion of cells expressing the metaprograms should be interpreted accordingly as it is possible for a given cell to also express other metaprogram(s) to a lesser degree. We do not have histologic vascularity data to formally address microvessel density correlation with metaprograms. Together, these data suggest that angiogenesis metaprogram expression in tumor cells reflects a transcriptional program intrinsic to malignant cells rather than a direct readout of the vascular composition of the TME.

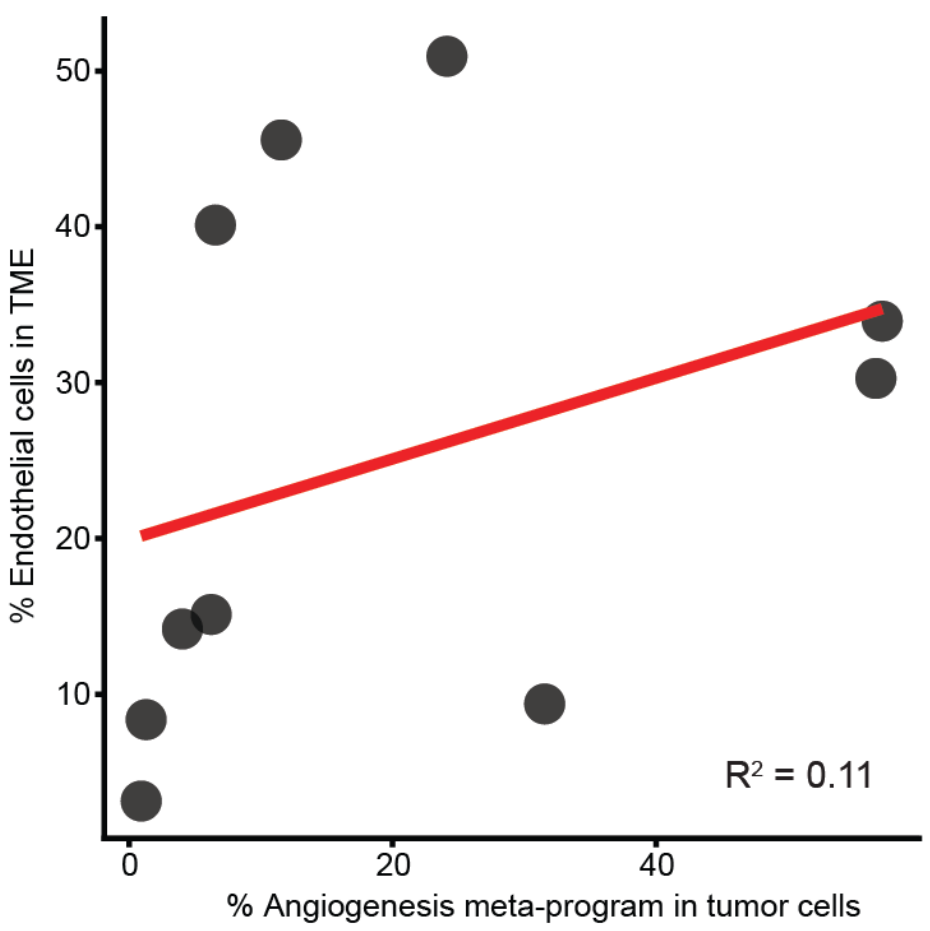

Rebuttal Figure 6. *Scatterplot showing the percentage of tumor cells expressing the angiogenesis meta-program (x-axis) versus the percentage of endothelial cells in the non-tumor compartment (y-axis) across tRCC snRNA-seq samples (frozen cohort). Red line indicates the linear regression fit.*

10. Including ligand–receptor cell–cell communication analysis for ccRCC (using ccRCC snRNAseq) alongside the tRCC results in Fig. 5D would help highlight key differences between tumor types.

We thank the reviewer for this excellent suggestion. We have now incorporated a more comprehensive comparative ligand-receptor cell-cell communication analysis between tRCC and ccRCC into the revised manuscript, addressing this comment at two levels.

First, in Rebuttal Figure 7A (Figure 5E in the revised manuscript), we present a comparative analysis of macrophage -> tumor cell interactions in tRCC versus ccRCC using cellphoneDB[12], identifying 62 tRCC-specific, 48 ccRCC-specific, and 26 shared interactions. This analysis reveals a striking difference in the nature of macrophage-tumor crosstalk between the two subtypes. In tRCC, macrophage -> tumor interactions are dominated by *SPP1*-integrin axes (*SPP1 -> ITGB1*/*ITGA9*, *SPP1 -> ITGAV*/*ITGB3*) and ECM-integrin signaling (*COL1A1*/*COL1A2*/*FN1 -> ITGB1*/*ITGAV*), consistent with the immunosuppressive and pro-stromal function of SPP1+ macrophages as the dominant myeloid population in tRCC. By contrast, ccRCC-specific macrophage -> tumor interactions are dominated by growth factor receptor signaling including *HBEGF -> EGFR*, *TGFA -> EGFR*, *VEGFA*/*B -> NRP1*, and *IGF1 -> IGF1R*, consistent with the well-established role of angiogenic and proliferative signaling in ccRCC biology driven by HIF pathway activation. These findings suggest that macrophages engage with tumor cells through fundamentally different signaling axes in tRCC versus ccRCC, reflecting both the distinct macrophage subtype composition and the distinct oncogenic programs operative in each subtype.

Second, as shown in Rebuttal Figure 7B (Figure S7E in the revised manuscript), we also present a broader summary heatmap of the number of significant interactions per cell type pair in tRCC vs. ccRCC, in all directions. This analysis demonstrates that tRCC exhibits generally higher numbers of significant interactions across nearly all cell type pairs relative to ccRCC, suggesting a more densely interconnected and potentially more immunosuppressive tumor ecosystem in tRCC overall.

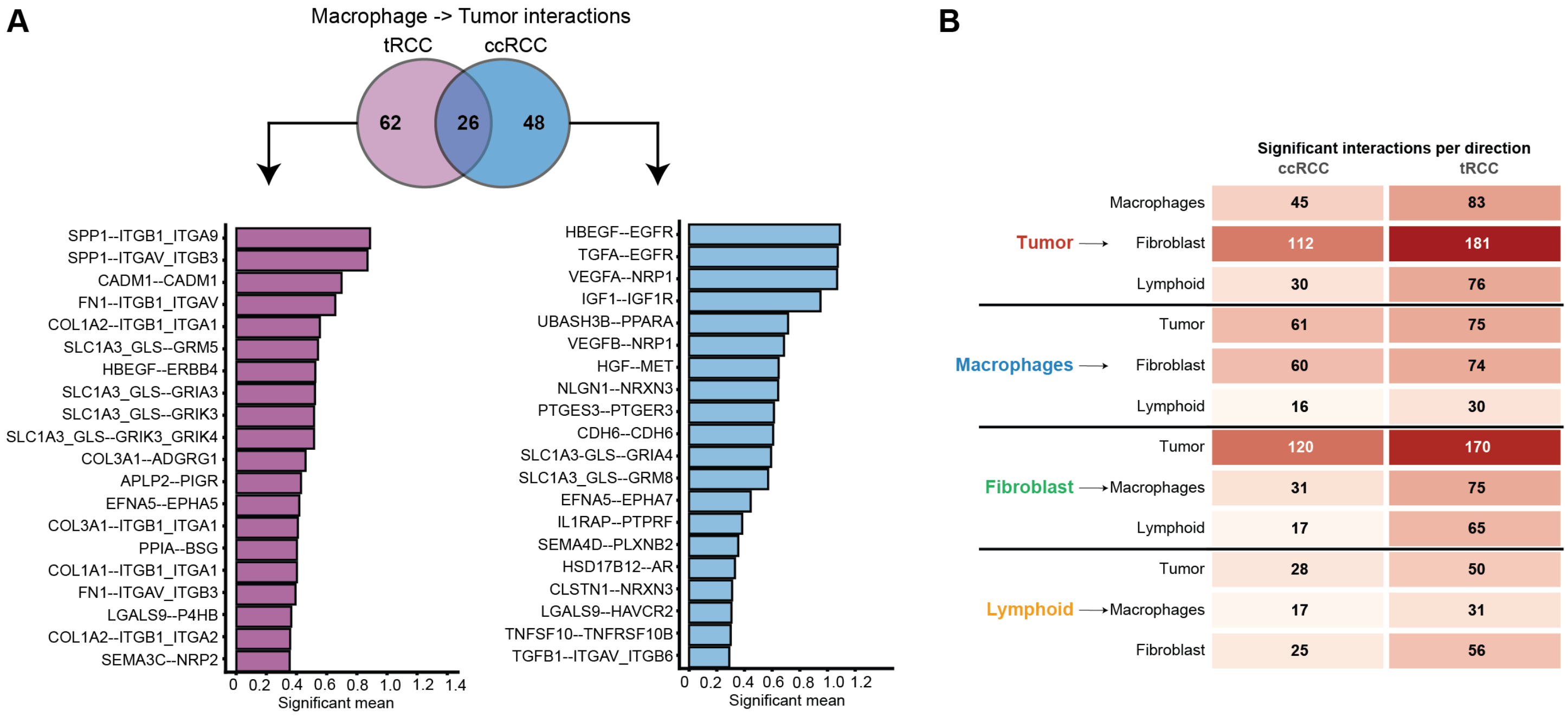

Rebuttal Figure 7. *Comparative ligand-receptor cell-cell communication analysis between tRCC and ccRCC. A. Venn diagram (top) showing the number of tRCC-specific, ccRCC-specific, and shared macrophage-to-tumor interactions identified via CellPhoneDB. Bar plots show the top tRCC-specific (left, pink) and ccRCC-specific (right, blue) interactions ranked by significant mean interaction strength. tRCC-specific interactions are dominated by SPP1-integrin axes and ECM-integrin signalin, while ccRCC-specific interactions are enriched for growth factor receptor signaling.* **(Figure 5E in revised manuscript).** *B. Heatmap summarizing the number of significant ligand-receptor interactions per cell type pair across all signaling directions in tRCC and ccRCC frozen snRNA-seq cohorts.* **(Figure S7E in revised manuscript)**.

Together, these analyses directly address the reviewer's suggestion and highlight that the immunosuppressive crosstalk we characterize in tRCC is not merely a quantitative amplification of interactions present in ccRCC, but reflects qualitatively distinct signaling axes that likely underlie the differential immune responsiveness observed between these two RCC subtypes. A discussion of these results is included in the revised text (lines 419-429 in revised manuscript).

11. The statement "Despite the genomic homogeneity of tRCC... we observed significant transcriptional heterogeneity both within and among tumors" raises the question of whether subclonal CNV differences contribute to this heterogeneity. Consider inferCNV analysis or similar approaches to explore this.

We thank the reviewer for this important suggestion. We have now performed inferCNV[13] analysis on malignant cells across our cohort to evaluate for inferred copy number alterations. Large-scale chromosomal alterations inferred from snRNA-seq (Rebuttal Figure 8; Figure S2A in revised manuscript) were largely consistent with matched whole-genome sequencing (WGS) data analyzed using TitanCNA published in our previous study[14], demonstrating concordance at arm-level and whole-chromosome level events.

For example, high-confidence CNV events identified by inferCNV overlapped with WGS-defined alterations in multiple samples, including chr6 loss and chr8 gain in BTRCC18; chr1p loss and chr1q gain in DTRCC10 and DTRCC6; and recurrent chr6q loss across several tumors.

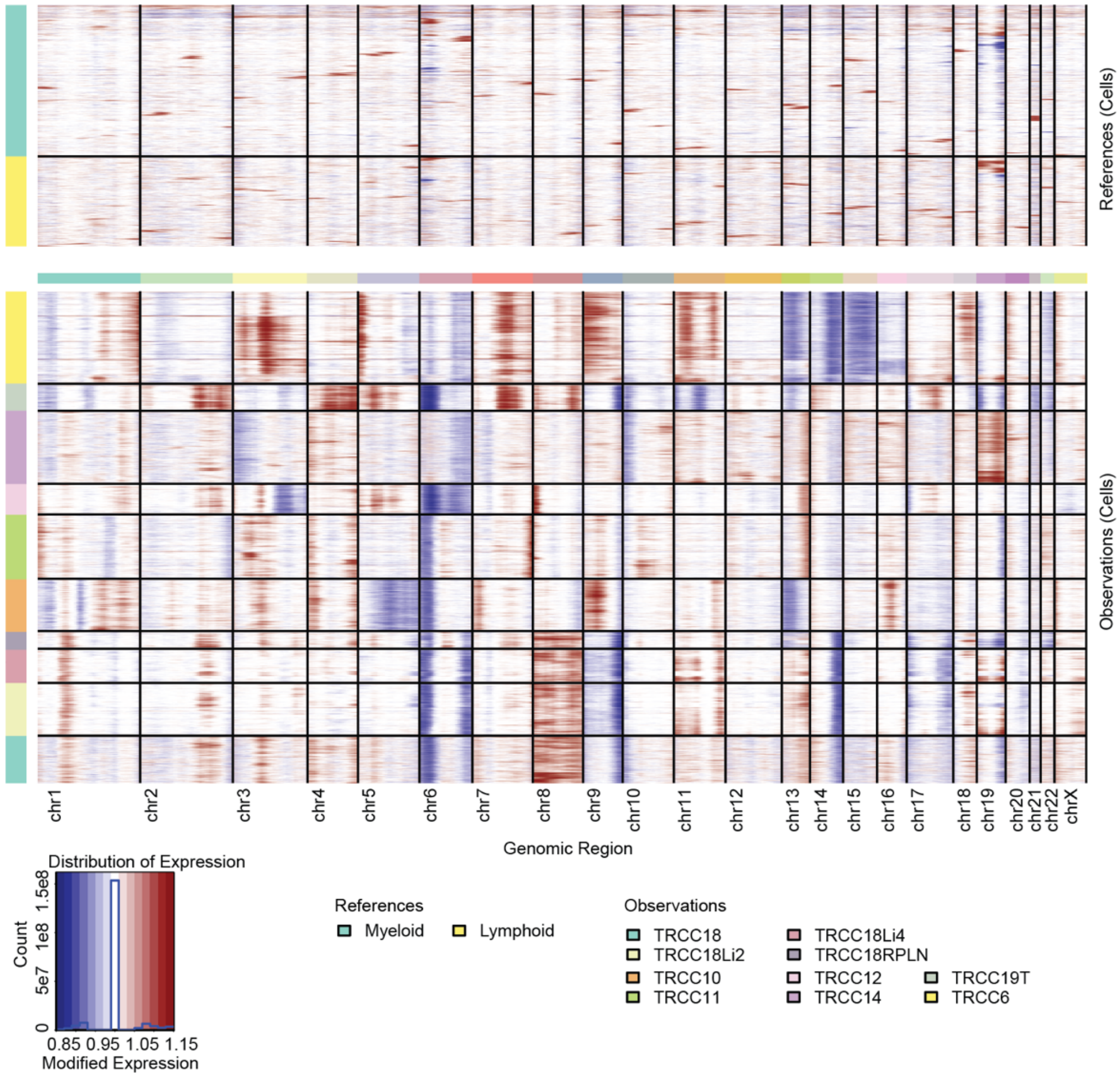

Rebuttal Figure 8 (Figure S2A in revised manuscript). InferCNV analysis of malignant cells across the tRCC snRNA-seq frozen cohort. Heatmap displays inferred copy-number variation across genomic regions for reference cells (myeloid and lymphoid; top) and tRCC tumor cells (bottom), with individual samples color-coded as indicated. Red indicates copy-number gain; blue indicates copy-number loss.

Importantly, within the limits of inferring subclonal copy number alterations from snRNA-seq data, we did not observe evidence for substantial copy number heterogeneity within malignant cells of a given tumor, suggesting that the heterogeneity in transcriptional states we observe within samples (Fig. 3) is not fully attributable to subclonal genomic alterations. Overall, these results suggest that the transcriptional diversity observed both within and among tumors is not primarily driven by somatic genetic changes but likely reflects epigenetic, regulatory, or microenvironmental influences that modulate transcriptional state. We have now included these analyses and a summary of concordant CNV regions in the revised manuscript (Figure S2A, lines 100-106 in revised manuscript).

12. Thank you for providing reviewer access to the GEO data. The GSE307120 entry appears incomplete:

o The 10X Flex data are not listed.

o A sample metadata table linking GEO samples to Supplementary Table-1 is missing, and the run selector link is empty.

o If possible, please also include count matrices to enable downstream reanalysis by future users.

We thank the reviewer for careful review of our GEO submission. We have confirmed that the 10X Flex datasets have now been uploaded to GSE307120 and will be publicly accessible upon publication of the manuscript. We also included processed count matrices within the processed.tar.gz files for each dataset. These files contain

gene-by-cell count matrices suitable for direct use in standard single-cell analysis workflows. In addition, we have contacted GEO support regarding the SRA Run Selector and the Sample metadata and were informed that both will be enabled once the submission is public.

Reviewer #2 (Remarks to the Author)

In this manuscript, Konda et al., present a comprehensive multi-omic study of tRCC. They provide snRNA-seq, snATAC-seq, spatial transcriptomics, and TCR-seq and contextualize the findings relative to ccRCC and pan-cancer datasets. The study has an impressive breath, but there appears to be a heightened focus on revalidating prior findings and there is a missed opportunity for discovery. In addition, most findings are correlative in nature.

We thank the reviewer for the thoughtful evaluation and for recognizing the breadth and scale of our findings. We have now substantially strengthened our study by carefully addressing each of the reviewer's specific comments below, in most cases through the inclusion of new experimental data and additional analyses.

Major Comments.

1. The cohort size is modest (15 tRCC cases) and highly heterogeneous. As shown in the t-SNE plots of Figure 1 while malignant cells from different samples (primary and metastatic) from the same patient (e.g., TRCC18) cluster together, samples harboring the same fusion (e.g., PRCC–TFE3: TRCC11, TRCC12, TRCC19) were broadly dispersed. Authors should clarify how this may confound the interpretation of the results.

We thank the reviewer for raising this point. While we agree that the cohort is modest in size and biologically heterogeneous in comparison to recent snRNA-seq studies of common cancers, the size of the cohort is very substantial when considering the rarity of tRCC; indeed, to our knowledge, a multiomic profiling study of this scale has not been previously performed for tRCC.

In our dataset, malignant cells cluster by patient of origin, and primary and metastatic samples from the same patient cluster together. This is consistent with snRNA-seq studies of other cancers, in which patient-specific differences exceed intra-patient (inter-site) differences[15,16].

By contrast, tumors harboring the same TFE3 fusion partner do not consistently segregate together. While fusion partner can impact target gene expression, there are limited data to suggest that the differences are stark enough to drive co-clustering that would dominate inter-patient differences, particularly in sparse snRNA-seq data (versus bulk RNA-Seq). Moreover, our study was not designed or powered to define fusion-specific transcriptional signatures, and the number of samples per fusion type precludes robust statistical analysis of fusion-stratified differences. Finally, the key tumor meta-programs and microenvironmental features described were observed across fusion types, supporting the generalizability of our findings within tRCC.

To address this reviewer's comment regarding fusion partners, we did perform an analysis of several bulk RNA-seq datasets including tRCC samples (total 134 samples across 5 datasets, Rebuttal Figure 9). After batch correction using Combat to merge datasets and PCA analysis, we find that samples do not segregate clearly by fusion partners. Future studies powered by a larger number of samples will be required to more definitively answer this question.

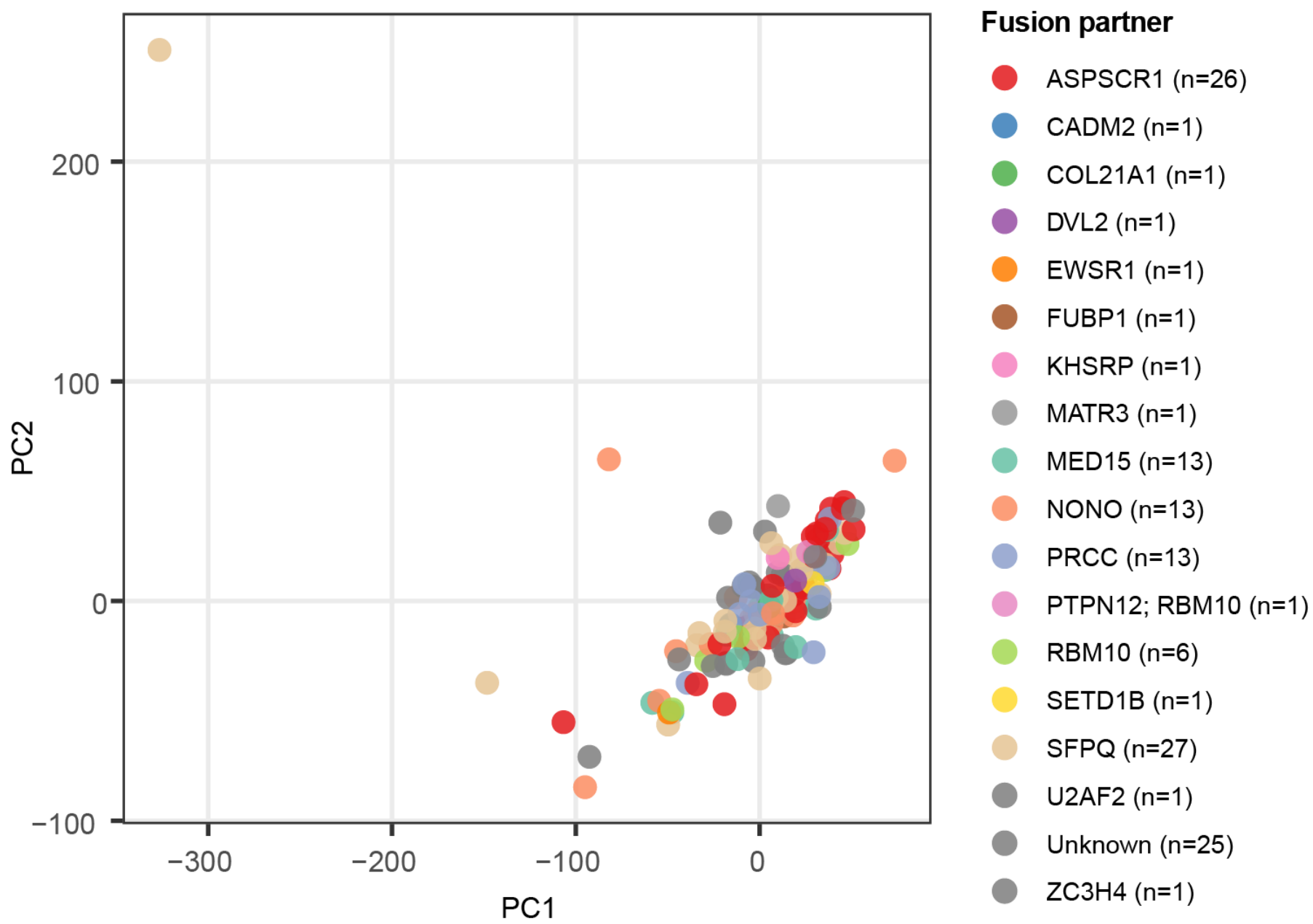

Rebuttal Figure 9. *Principal component clustering of tRCC bulk RNA-seq samples from publicly available datasets. Scatter plot of principal components 1 and 2 (PC1 and PC2) derived from PCA of batch-corrected bulk RNA-seq gene expression data aggregated across 5 independent tRCC datasets (total N=134 samples), colored by fusion partner.*

2. *A more systematic approach (e.g., enrichment scores, adjusted p-values/FDR, effect sizes) for all pathway enrichment analyses (Figure 2) should be provided beyond selected pathways.*

We thank the reviewer for this suggestion. To clarify our selection approach, pathways were ranked by Fold Enrichment and only the pathway terms with FDR < 0.05 were selected from each database (Hallmark, KEGG, and GO terms). We have now included Rebuttal Figure 10A (Figure 2A in revised manuscript) where bars are colored by exact FDR value. Because all displayed pathways meet the FDR < 0.05 threshold, the color gradient reflects the degree of statistical confidence within this significance-filtered set rather than a mix of significant and non-significant terms.

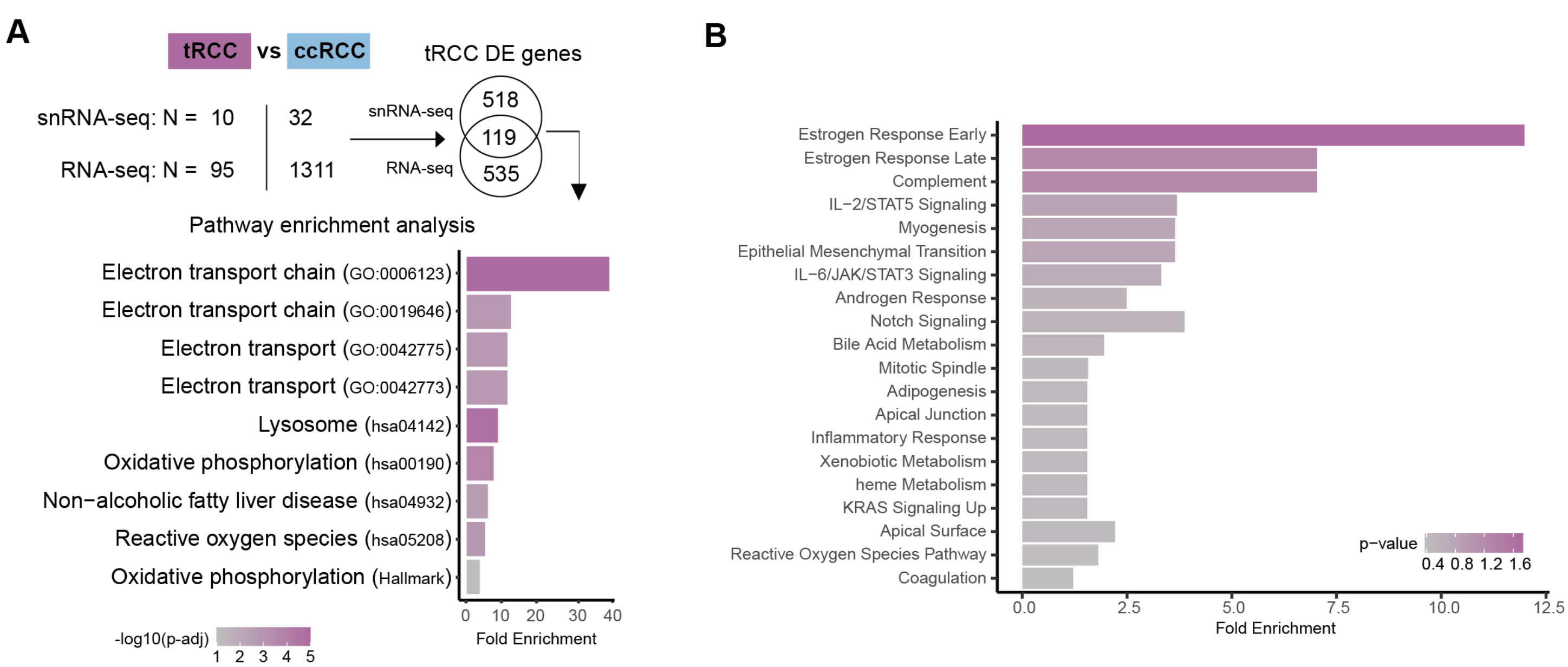

Rebuttal Figure 10. *Pathway enrichment analyses for tRCC vs. ccRCC differentially expressed genes and snATAC-seq data. A. (Left, revised Fig. 2A in manuscript) Pathway enrichment analysis of 119 consensus tRCC differentially expressed genes identified by intersection of snRNA-seq and bulk RNA-seq comparisons with ccRCC. Bar plots show fold enrichment for significantly enriched pathways (FDR < 0.05) across GO and KEGG databases, with bar color reflecting -log10(adjusted p-value)* **(Figure 2A in revised manuscript)**. *B. (Right, not*

included in revised manuscript) Hallmark pathway enrichment analysis performed on tRCC snATAC-seq data using peak-to-gene assignments. Bar color reflects p-value as indicated.

3. An explanation is needed for why the proximal tubule (PT) and EMT signatures appear mutually exclusive between primary and metastatic samples (Figure S3).

We thank the reviewer for this comment. The mutual exclusivity of PT and EMT programs likely reflects a biologically relevant plasticity between epithelial lineage identity and a "pro-metastatic" mesenchymal state. We believe that the enrichment of EMT and depletion of PT program at metastatic relative to primary sites reflects selective outgrowth of EMT-high cells during metastasis, consistent with a body of literature associating EMT transcriptional programs with increased migratory and invasive capacity during metastatic dissemination[17,18]; a cooperating factor may be organotropism that favors malignant cell dissemination to non-renal microenvironments[19,20] that lack the signals required to maintain proximal tubule identity.

While we believe our data regarding the mutual exclusivity between EMT/PT programs are compelling – and substantially strengthened by paired primary and metastatic sites from the same individual, we acknowledge that the nature of our study limits this to a correlative observation. Nonetheless, this observation is consistent with many well-established functional models of EMT and metastasis.

We have expanded on this discussion in the revised manuscript (lines 241-248).

4. The snATAC–ChIP integration studies should be extended evaluating enrichment at pathway/ gene set levels (e.g. EMT).

We thank the reviewer for this suggestion. We performed pathway-level enrichment analysis on chromatin accessibility data from tRCC snATAC-seq, using peak-to-gene assignments to assess enrichment of Hallmark gene sets (Rebuttal Figure 10B). While EMT was among the top enriched pathways, this did not reach statistical significance. This may reflect the known limitations of inferring pathway activation from chromatin accessibility alone, where accessibility at regulatory elements reflects transcriptional potential rather than active gene expression, and concordance with RNA-level pathway activity is incomplete[21–23]. Moreover, our power for identifying accessibility-associated pathways is more limited than for snRNA-seq, given that snATAC-seq was performed only on a subset of the cohort (N=4 samples from 2 patients). Despite these limitations, snATAC-seq integration is powerful in being able to nominate fusion-associated regulatory elements and direct TFE3 fusion target genes, as shown in Figure 2.

Nonetheless, we believe that the multiple lines of evidence presented in the manuscript, including the NMF-derived EMT meta-program, the TFE3 fusion perturbation experiments, and the GSEA analyses in cell lines and murine models provide a more reliable and interpretable readout of EMT pathway activation in tRCC than can be obtained from chromatin accessibility data alone.

5. While limited by numbers, it would be interesting to assess whether EMT, SPP1, and mCAF signatures predict survival independently of fusion partner type.

We thank the reviewer for this suggestion. Our original multivariate Cox model included four covariates (EMT, mCAF, SPP1, and tumor stage); we have now added fusion partner as a binary covariate (ASPSCR1-TFE3 vs. non-ASPSCR1, the only feasible subgroup comparison given the size and fusion partner distribution in this cohort), yielding a five-covariate model with 7 events across 26 patients (Rebuttal Figure 11; Figure 6G in revised manuscript).

As shown below, SPP1+ macrophage score retained statistical significance after adjusting for fusion partner identity (HR 5.73, 95% CI 1.05-31.38, p=0.044), while fusion partner itself was not a significant predictor of overall survival (HR 0.49, 95% CI 0.013-17.73, p=0.694). Advanced stage also remained an independent predictor of worse outcomes (HR 57.55, 95% CI 1.99-1661, p=0.018). EMT and mCAF scores remained non-significant in this expanded model. The retention of SPP1+ macrophage score as an independent predictor after adjusting for fusion partner provides additional evidence that this prognostic association is not simply a reflection of fusion partner-driven differences in tumor aggressiveness.

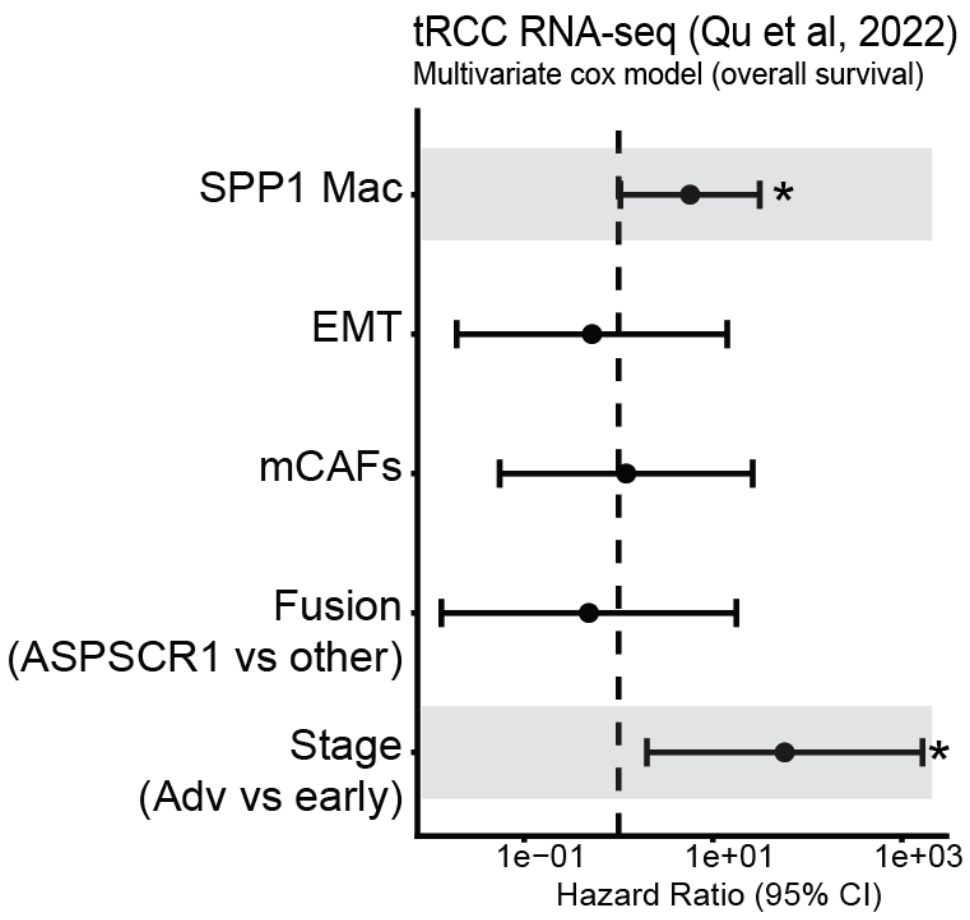

Rebuttal Figure 11 (Figure 6G in revised manuscript). *Multivariate Cox proportional hazards model for overall survival in the Qu et al. tRCC bulk RNA-seq cohort (N=26 patients, 7 events), incorporating EMT score, mCAF score, SPP1+ macrophage score, tumor stage (advanced vs. early), and fusion partner (ASPSCR1-TFE3 vs. other) as covariates. Forest plot shows hazard ratios with 95% confidence intervals.*

6. It would be good to expand on why the TME differs between ASPS and other fusions and how it may affect ICI responsiveness.

We thank the reviewer for this suggestion. We wish to clarify that our study was not designed to compare TME composition across TFE3 fusion partner subtypes within tRCC, and our snRNA-seq cohort includes does not include samples with ASPSCR1-TFE3 fusion. We therefore cannot directly address this question in the current manuscript.

We acknowledge that fusion partner identity may influence tumor biology and potentially TME composition. For example, Sun et al.[24] reported that ASPSCR1-TFE3 fusions are associated with more aggressive disease and enrichment of stromal and angiogenic signatures in bulk RNA-seq data, suggesting that fusion partner-specific transcriptional programs may exist. However, whether these transcriptional differences translate to meaningful differences in TME composition, immune infiltration, or ICI responsiveness at the cellular level remains unknown and would require cohort sizes and a scale of sequencing that would be challenging to achieve given the incidence of tRCC. Performing such an analysis in our cohort, which was assembled to characterize tRCC biology as a whole rather than to compare fusion subtypes, would require substantially larger fusion partner-stratified cohorts than are currently available.

However, we agree with the reviewer in the importance of this question, and have accordingly acknowledged fusion partner-specific TME characterization as an important and clinically relevant future direction in the revised Discussion (lines 521-530).

7. The immune deconvolution analyses indicating macrophage enrichment and a reduction in CD8 T cells should be validated by IHC ideally in a larger cohort.

We thank the reviewer for this important suggestion and are pleased to report that we have substantially expanded the validation of our immune deconvolution findings through both analytical refinements and orthogonal experimental validation (see Response to Reviewer #1, Comments 4,5).

As described in detail in our response to Reviewer #1, we have revised the immune deconvolution analysis in Figure 1E to account for tumor purity and to provide estimates of absolute infiltration (rather than proportional). These analyses demonstrate significantly higher macrophage infiltration and significantly lower CD8+ T cell abundance in tRCC relative to ccRCC across both the TCGA and IMmotion151 cohorts. We also provide more granular macrophage subtype estimates suggestive of lower M1/M2 abundance in tRCC relative to ccRCC (Rebuttal Figure 3A-B; Figure S2B-C in revised manuscript). Overall, these results are consistent with our single-cell finding that SPP1+ anti-inflammatory macrophages are enriched in tRCC while IFN-primed macrophages are absent.

We have also validated the reduction in CD8+ T cell infiltration in tRCC using multiplex immunofluorescence (IF) on a cohort of 10 tRCC and 11 ccRCC cases (Rebuttal Figure 4; Figure 1F in revised manuscript). This analysis demonstrates significantly lower CD8+ and CD8+/PD1+ populations in tRCC relative to ccRCC, with the

exception of one outlier case, providing direct orthogonal support for the immune deconvolution findings at the protein level in primary tumor tissue.

We have further validated the macrophage findings using multiplex IF on the same cohort. SPP1+/CD163+ macrophages are significantly more abundant in tRCC compared to ccRCC (Rebuttal Figure 12; Figures 5C-D in revised manuscript; lines 400-403), directly confirming at the protein level our single-cell findings. Moreover, MRC1+CD163+ macrophages, a broadly M2-polarized population, show no significant difference between tRCC and ccRCC. This suggests that the overall burden of M2-like macrophages is not dramatically different between the two subtypes, and that the functionally relevant distinction in tRCC lies specifically in the enrichment of SPP1+ macrophages within the broader M2 compartment.

Altogether, our updated analyses and multiplex IF validation substantially strengthen our conclusions regarding the specificity of SPP1+ macrophage enrichment in tRCC.

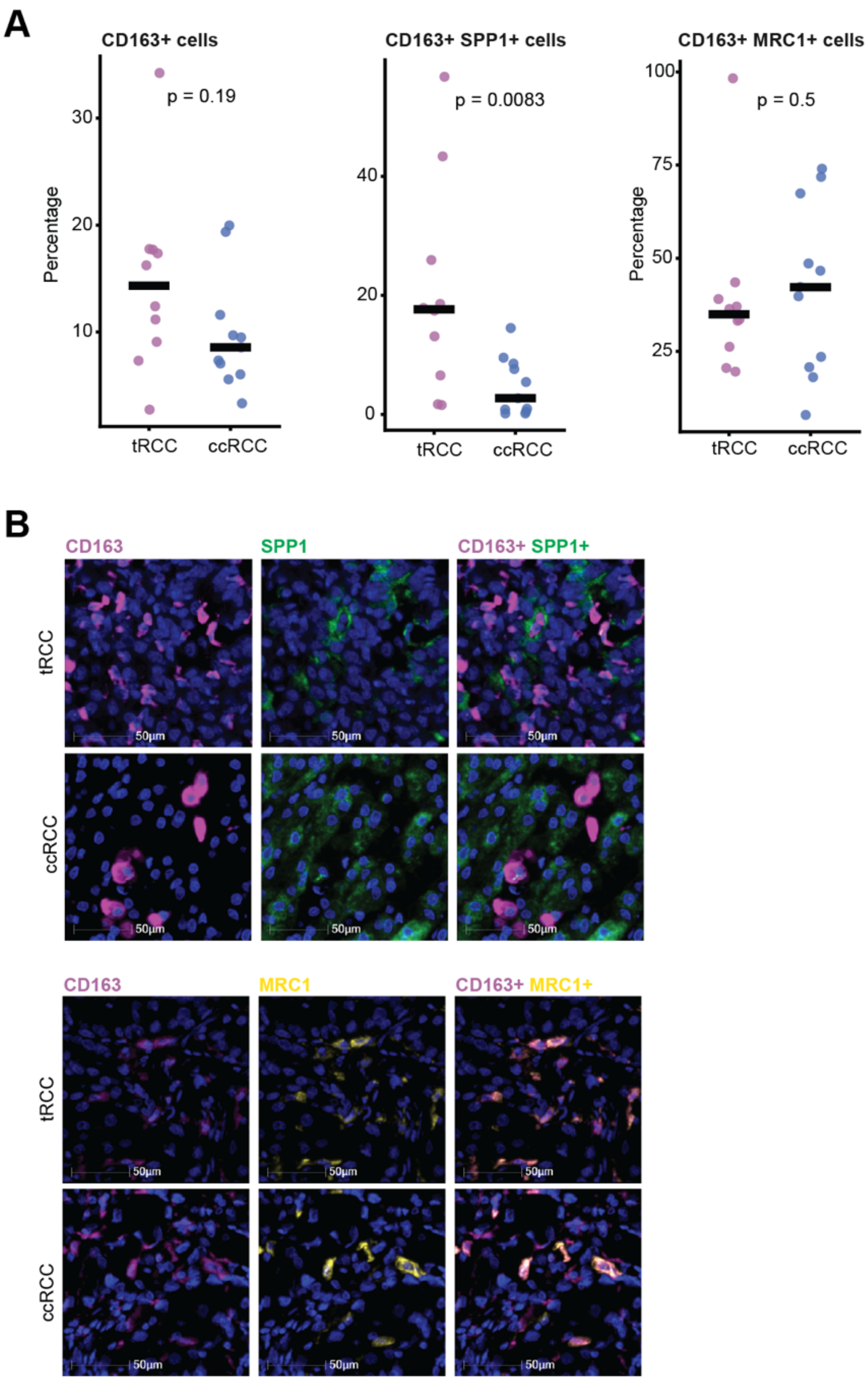

Rebuttal Figure 12 (Figures 5C-D in revised manuscript). *Multiplex immunofluorescence (IF) validation of macrophage subtype composition in tRCC and ccRCC. A. Quantification of the percentage of CD163+ cells (left), CD163+SPP1+ cells (middle), and CD163+MRC1+ cells (right) in tRCC (N=10) and ccRCC (N=11) cases. P-values were calculated by Wilcoxon rank-sum test. B. Representative multiplex IF images showing CD163 (pink), SPP1 (green), and CD163+SPP1+ cells (top panels) and CD163 (pink), MRC1 (yellow), and CD163+MRC1+ cells (bottom panels) in representative tRCC and ccRCC cases. Scale bars, 50 μm.*

8. The link between TFE3 fusion and EMT is intriguing but remains correlative. Functional validation such as knockdown/rescue experiments or CRISPR-based perturbations would be important to strengthen the causal relationship between TFE3 fusion and EMT induction.

We thank the reviewer for this important suggestion. Our revised manuscript now includes several functional experiments to address the causal relationship between TFE3 fusion activity and EMT induction (Rebuttal Figure 13; Figure S5 in revised manuscript).

First, to directly test whether TFE3 fusion activity is linked to expression of the EMT program, we performed shRNA-mediated knockdown of the endogenous TFE3 fusion in three tRCC cell lines (FUUR1, UOK120, and UOK124) and assessed EMT pathway activity by gene set enrichment analysis (GSEA). Fusion knockdown resulted in significant negative enrichment of the Hallmark EMT gene set across all three cell lines (Rebuttal Figure 13A), indicating that TFE3 fusion activity is required to maintain the EMT transcriptional program in tRCC cells. Consistent with this transcriptional finding, wound healing assays (Rebuttal Figure 13B) in FUUR1 cells demonstrated significantly reduced migratory capacity upon fusion knockdown relative to wildtype controls, providing functional evidence that fusion-driven EMT has measurable phenotypic consequences in a relevant *in vitro* assay.

Finally, to assess whether the fusion-EMT relationship observed in patient data and cell lines is recapitulated in vivo models of tRCC, we examined EMT enrichment in two published TFE3 fusion-driven genetically engineered mouse models (one expressing SFPQ-TFE3 fusion[25], and a second expressing an ASPSCR1-TFE3 fusion[26]) (Rebuttal Figure 13C). GSEA demonstrated significant positive enrichment of the Hallmark EMT gene set in tumor samples relative to adjacent normal kidney in both models, confirming that EMT induction is a conserved feature of TFE3 fusion-driven oncogenesis across multiple fusion partners and across both human samples and existing *in vivo* models.

While the transcriptional and functional data above support a causal role for TFE3 fusion activity in EMT induction, whether this is direct (i.e. through the activation of one or more downstream master regulators) or indirect, remains to be elucidated, and is beyond the scope of the current study.

We have incorporated these findings and a mechanistic discussion into the revised manuscript, including updated text in the Results and Discussion sections (lines 258-269 & 476-480). While more detailed mechanistic studies linking the TFE3 fusion to initiation and maintenance of EMT are an active area of investigation in our laboratory and others, we believe that the new convergent evidence from three tRCC cell lines, two *in vivo* mouse models, and the patient-derived single-cell and bulk transcriptomic data presented in our revised manuscript collectively support a functional relationship between TFE3 fusion activity and EMT induction in tRCC.

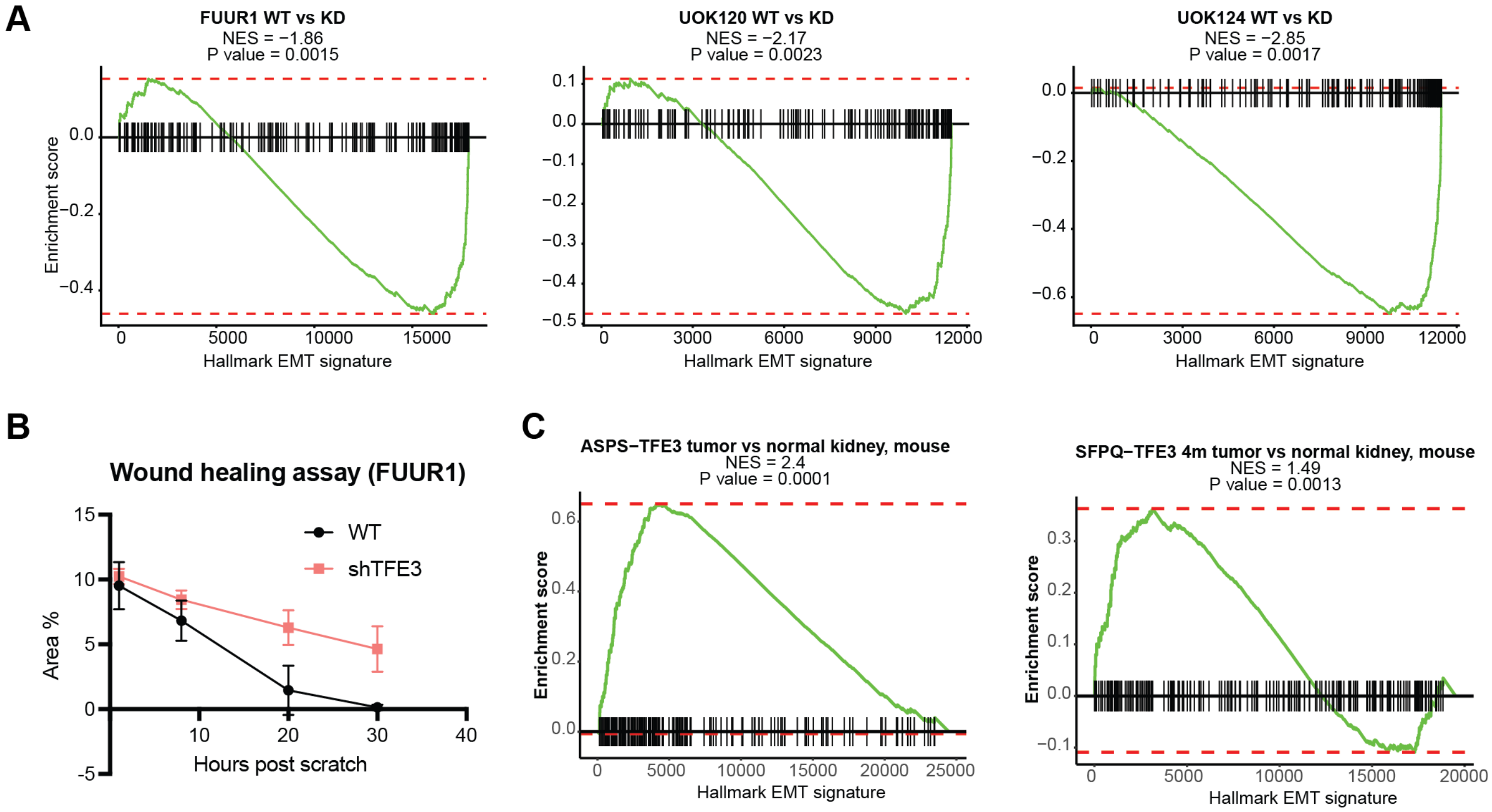

Rebuttal Figure 13 (Figure S5 in revised manuscript). *Functional validation of TFE3 fusion activity in EMT induction. A. GSEA plots showing depletion of the Hallmark EMT gene signature upon TFE3 fusion knockdown in three tRCC cell lines harboring distinct fusion partners: FUUR1 (ASPSCR1-TFE3; NES = −1.86, p = 0.0015), UOK120 (PRCC-TFE3; NES = −2.17, p = 0.0023), and UOK124 (PRCC-TFE3; NES = −2.85, p = 0.0017). WT, wild-type; KD, knockdown. B. Wound healing assay in FUUR1 cells expressing wild-type TFE3 fusion (WT) or TFE3 fusion knockdown (shTFE3), showing percentage of open wound area remaining at 0, 8, 20, and 30 hours post-scratch. Data represent mean ± SEM. C. GSEA plots showing enrichment of the Hallmark EMT gene signature in murine ASPS-TFE3 tumors (NES = 2.4, p = 0.0001) and SFPQ-TFE3 4-month tumors (NES = 1.49, p = 0.0013) relative to normal kidney tissue.*

9. Discovery of SPP1⁺ macrophages and mCAFs as suppressive hubs is novel. While spatial data confirmed proximity, functional validation would greatly strengthen the therapeutic implications, but understandably, this may be beyond the scope of this paper.

We thank the reviewer for this thoughtful comment. We agree that functional validation of the suppressive interactions between $SPP1^{+}$ macrophages and mCAFs would further strengthen the therapeutic implications of these findings. In the current study, we focused on integrating single-cell transcriptomic, chromatin, and spatial profiling approaches to identify and spatially validate these cellular hubs within the tumor microenvironment. While mechanistic interrogation of these interactions is an important next step (and indeed is the subject of our ongoing studies), as the reviewer points out, doing so rigorously would require experiments and complex models that are beyond the scope of the current manuscript.

Minor comments:

1. Please clarify the methodology used to process and integrate snRNA-seq data from fresh frozen (3' snRNA-seq and 3' Multiome) and FFPE samples. Since multiple platforms were combined, how were batch effects identified, addressed, and corrected? Providing additional QC metrics would further strengthen confidence in the integration and interpretation of these data.

We thank the reviewer for this question. We would like to clarify that the frozen and FFPE datasets were not integrated. As described in Results section 1.1 (lines 106-108), while frozen and FFPE platforms yielded broadly comparable feature distributions, differences in cell type proportions, likely related to sample type preparation differences, led us to analyze these cohorts separately (Figure S1D-E).

Within the frozen cohort, 3’ snRNA-seq and 3’ Multiome datasets were merged following standard preprocessing workflow using Seurat as described in the methods section. Batch effects were assessed by examining clustering patterns before integration and were corrected using Harmony, with sequencing batch and patient identity

included as covariates, as described in the Methods section ("Single-nucleus RNA sequencing data processing and analysis"). Accordingly, post-integration inspection of UMAP embeddings demonstrated mixing of samples across batches in non-malignant cells, which clustered by cell type rather than by patient or batch, while tumor cells clustered separately by patient (Figure S1C).

2. Please provide the specific gene sets used to calculate the EMT score in Figure 3 and explain why COL1A1, SPP1, or VCAN (top-ranked in the NFM EMT signature) are not among the 113 fusion-regulated genes (Figure 3C).

We thank the reviewer for this comment. The EMT score in Figure 3 is derived from the NMF-based EMT meta-program gene signature derived directly from our snRNA-seq data, and the TFE3 fusion signature (113 genes) is derived by intersecting two independent *in vitro* perturbation experiments (TFE3 fusion overexpression in HEK293T cells and knockdown in FUUR1 cells). Both gene lists are provided in Supplementary Table S3.

These two signatures serve fundamentally different purposes and are not expected to overlap substantially. COL1A1, SPP1, and VCAN are among 33 genes in the NMF signature highly expressed in EMT-high malignant cells in patient tumors but their regulation by the TFE3 fusion is likely context-dependent and would not necessarily be expected to be observed across the cell line models used to derive the overall fusion signature. This is for multiple reasons, including: 1) the fusion likely induces a host of genes related to other non-EMT pathways; 2) some loci accessible in tumors may not be accessible for gene activation in exogenous/*in vitro* systems; 3) EMT-related pathway expression may be incompletely captured in pure cell line models (i.e. without co-culture of microenvironmental cell types).

3. Did the authors account for the 15 TCGA tRCC cases that were not annotated or subclassified? If so, were these excluded prior to analysis?

We thank the reviewer for this question. The TCGA dataset used in our analysis contains 17 tRCC cases, all of which were accounted for and included in our bulk RNA-seq analyses/ comparisons between tRCC vs ccRCC (Figures 1D-E and Figure 2).

Reviewer #3 (Remarks to the Author)

I co-reviewed this manuscript with one of the reviewers who provided the listed reports. This is part of the Nature Communications initiative to facilitate training in peer review and to provide appropriate recognition for Early Career Researchers who co-review manuscripts.

Reviewer #4 (Remarks to the Author):

I co-reviewed this manuscript with one of the reviewers who provided the listed reports. This is part of the Nature Communications initiative to facilitate training in peer review and to provide appropriate recognition for Early Career Researchers who co-review manuscripts.

Reviewer #5 (Remarks to the Author):

In this manuscript, Konda et al describe a comprehensive analysis of single-cell transcriptomes and chromatin accessibility profiles from translocation renal cell carcinoma (tRCC), an aggressive RCC defined by oncogenic TFE3 gene fusions. The authors assembled a cohort of 20 tRCC samples which were profiled using single-nucleus RNA sequencing (snRNA-seq), along with single nucleus ATAC sequencing (snATAC-seq), spatial transcriptomic analysis, and T Cell Receptor (TCR) sequencing. By integrating 128 bulk transcriptomic tRCC profiles from prior studies and a systematic comparison with 1300 published ccRCC bulk and 32 single-cell transcriptional profiles, authors present a detailed picture of how TFE3 fusions drive malignancy and contribute to immunotherapy resistance.

This is an important contribution to understanding the biology of an understudied cancer type. There are several issues that should first be addressed.

We thank the reviewer for their thoughtful review, encouraging assessment and for recognizing the importance of this contribution to understanding tRCC biology. We have carefully addressed each of the reviewer's comments below by incorporating additional analyses into our revised manuscript.

MAJOR COMMENTS:

- For the analysis in Figure 1E, a claim is made that there are more macrophages in tRCC, upon immune deconvolution. This claim could use further support. First, what was counted as macrophage from CIBERSORTx (assuming LM22 matrix was used)? M2? M1?. Second, it looks like the relative abundance of immune populations were shown, but we know there are dramatic differences in immune infiltration between tumor types. It would be helpful to examine the differences using "absolute" mode for CIBERSORTx. Lastly, there is no statistical comparison done – it would be more convincing if a statistical test were performed showing that there is significantly higher macrophages levels in tRCC compared to the other tumor types.

We thank the reviewer for these important suggestions. As discussed in detail in response to Reviewer #1, comments #4 and #5, we have updated our CIBERSORTx and TIMER analysis to confirm our underlying finding of increased macrophage infiltration in tRCC relative to ccRCC, with a trend toward lower M1/M2 ratio in tRCC vs. ccRCC. Statistical comparisons were performed across tumor types for both algorithms (Rebuttal Figure 2B and 3; Figures 1E and S2B in revised manuscript). These results alongside appropriate caveats regarding the limitations of these immune deconvolution algorithms are described in the updated text (lines 117-127). Additionally, the core findings have also now been validated by mIF in our revised manuscript.

- In figure 3, the authors chose to separately analyze the snRNAseq from frozen and FFPE, which is reasonable. However, for the tumor metaprogram analysis, it is unclear whether this is found in the snRNA-seq from frozen, from FFPE, or from both. The authors should clarify this, and if only performed in one (e.g. frozen), it would be helpful to validate in the other.

We thank the reviewer for this important comment. The tumor metaprogram analysis was performed independently in both the frozen snRNA-seq cohort and the FFPE 10X Flex cohort. In each dataset, we identified highly similar tumor-intrinsic transcriptional programs using the same analytical framework. We treated each cohort separately owing to sample type preparation differences that could otherwise confound integrating the two datasets.

For clarity, Figures 3A-E and 3G present results from the frozen cohort, while Figure 3J and Supplementary Figures S3A and S3C (updated Figures S4A and S4C) include validation analyses in the FFPE cohort. We have revised the text (lines 221-222) in the Results section to more explicitly state that the metaprogram analysis was

performed in both cohorts independently and that comparable programs were independently identified across platforms.

- For figure 4, it would be helpful to gain some confidence around the claim that CD8 c1 is a progenitor exhausted cluster, vs simply a non-exhausted population. Does this cluster express intermediate levels of PD1? Any TOX expression? It is very possible that c1 is simply a non-exhausted CD8 T cell population.

We thank the reviewer for this thoughtful comment. Upon further analysis of both clusters, the CD8 c1 population demonstrates intermediate *PDCD1* expression and detectable *TOX* expression relative to CD8 c2, while lacking high levels of terminal exhaustion markers such as *HAVCR2*, *LAG3*, and *ENTPD1* (which are higher in CD8 c2). In addition, CD8 c1 retains expression of stem-associated markers including *IL7R*, *TCF7*, *SLAMF6*, and *CXCR4*. To clarify this distinction, we have revised Figure 4A (Rebuttal Figure 14) to include additional exhaustion and progenitor-associated markers and updated the graph to better illustrate the exhaustion continuum. We have also modified the text (lines 345-352) to more precisely describe CD8 c1 as exhibiting a progenitor-like/early exhausted phenotype rather than implying a fully canonical progenitor exhausted state.

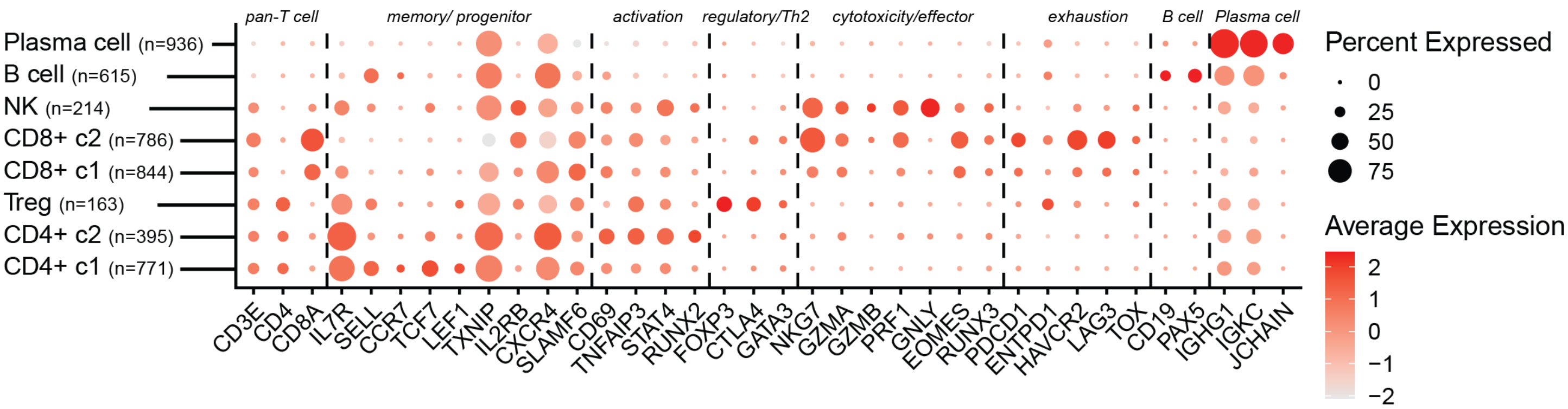

<u>Rebuttal Figure 14 (Figure 4A in revised manuscript)</u>. *Dot plot depicting lymphocyte subpopulations identified in the tRCC 10x Flex cohort (N=5 samples, 4724 nuclei), with an expanded marker panel to characterize the CD8+ T cell exhaustion continuum. Dot size represents percent of cells expressing each gene; color represents average expression level.*

- For the analysis in 4E, again here to make the claim that a certain population is expanded, a statistical comparison should be done on a per sample basis (e.g. the proportion of CD8 c1 in each sample with ICI compared to each sample without ICI).

We agree with the reviewer that statistical comparison of cluster proportions should ideally be performed on a per-sample basis. However, in the 10X Flex FFPE cohort, only one tumor was derived from an ICI-treated patient, precluding meaningful statistical comparison. We have therefore revised the text (lines 350-356) to clarify that the observed enrichment represents a descriptive trend rather than a statistically tested expansion. Importantly, a similar enrichment of progenitor-like CD8 T cells in ICI-exposed samples was observed in the independent frozen snRNA-seq cohort, providing orthogonal support for this observation, within the limits of the relatively small cohort size.

- For the spatial analysis in figures 5F and 5G, it would be helpful to provide some quantification / summary statistics to confirm the claim of the receptor-ligand interactions.

We thank the reviewer for this suggestion. We have addressed this comment at two levels in the revised manuscript.

For the specific SPP1+ macrophage and matrix CAF interactions highlighted in the main figures, we have added a dot plot (Rebuttal Figure 15; Figure S7G in the revised manuscript) displaying interaction specificity scores and expression magnitude for each ligand-receptor pair across target cell types in the tRCC 10x Flex cohort. This provides quantitative summary statistics confirming that the interactions highlighted including SPP1->ITGAV/ITGB axes and COL1A1->integrin interactions are both highly specific to and strongly expressed in the relevant cell type pairs.

Additionally, we have now performed spatial proximity analysis directly on the CosMx spatial transcriptomic data to quantitatively support the co-localization patterns shown in Figures 5F and 5G (Figures 6C, 6D in revised manuscript). Using cell centroid coordinates, we computed the nearest-neighbor distance from each SPP1+ macrophage to the closest ITGAV+ tumor cell, and from each COL1A1+ fibroblast to the closest ITGAV+ tumor

cell, within a 20 µm proximity threshold. In TRCC6 (Figure 6C), 51.5% of SPP1+ macrophages were within 20 µm of an ITGAV+ tumor cell, and 15.0% of COL1A1+ fibroblasts were within 20 µm of an ITGAV+ tumor cell. In TRCC14 (Figure 6D), 4.1% of SPP1+ macrophages were within 20 µm of CD44+ T cells. These proximity metrics provide quantitative confirmation of the spatial co-localization patterns depicted in the figures (lines 440-443 in the revised manuscript).

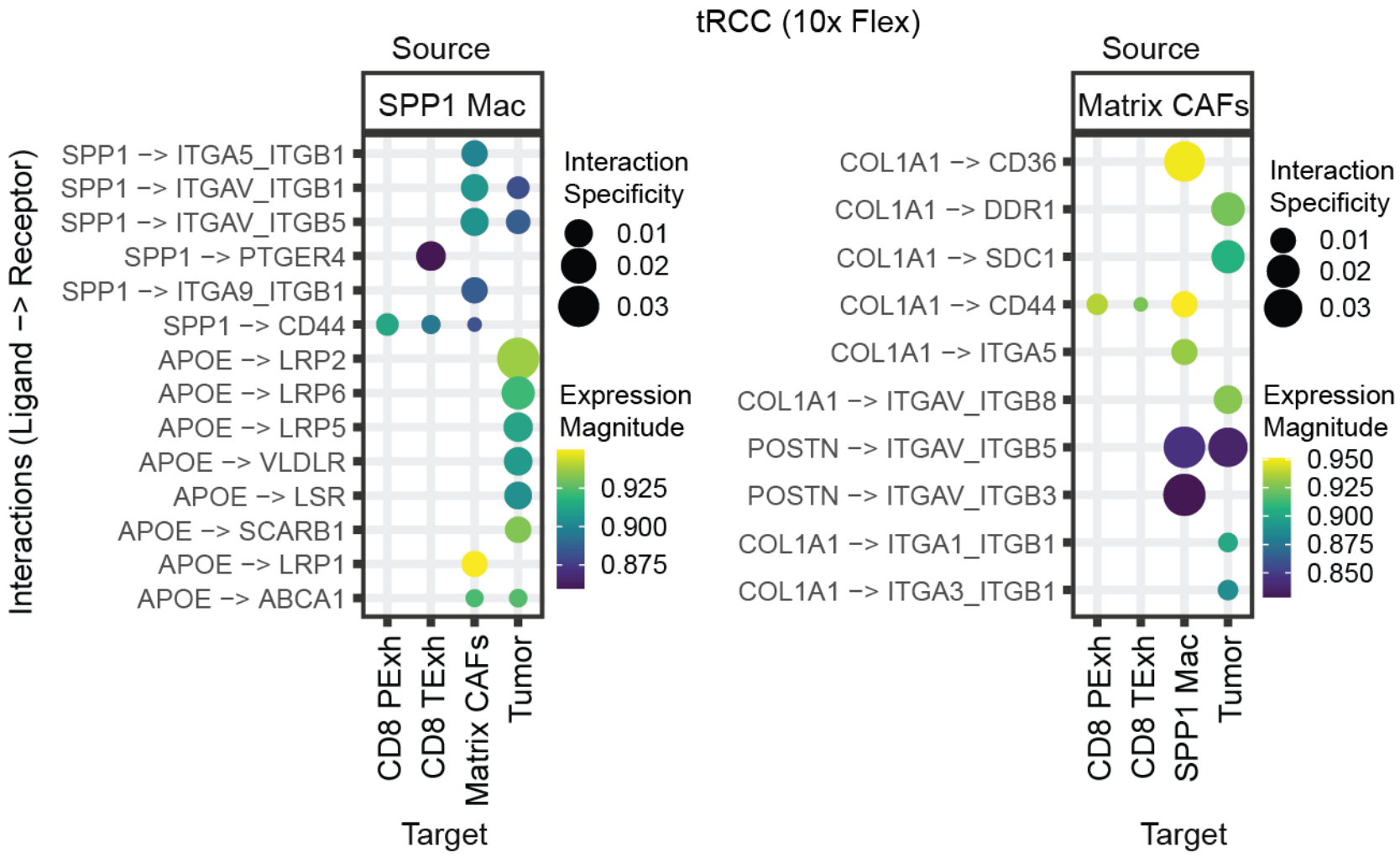

Rebuttal Figure 15 (Figure S7G in revised manuscript). *Dot plots showing interaction specificity and expression magnitude of ligand-receptor interactions in the tRCC 10x Flex cohort, with SPP1+ macrophages (left) and matrix CAFs (right) as sender cell types. Dot size represents interaction specificity score; color represents expression magnitude.*

Additionally, as also discussed in detail in response to Reviewer 1, Comment #10, we have performed a comparative ligand-receptor analysis between tRCC and ccRCC (snRNA-seq, frozen cohorts) at the level of macrophage-to-tumor interactions (Rebuttal Figure 7A; Figure 5E in revised manuscript), identifying tRCC-specific, ccRCC-specific, and shared interactions, with significant mean interaction strength shown for each interaction. A broader summary of significant interaction counts across all cell type pairs is additionally provided in Rebuttal Figure 7B (Figure S7E in revised manuscript).

MINOR COMMENTS:

- The total number of cells (post QC) for each dataset (snRNASeq) can be mentioned in the main text or in a supplemental table

We thank the reviewer for this suggestion. We have now included the total number of post-QC nuclei for each snRNA-seq dataset, as well as for the snATAC-seq datasets, in additional sheets of Supplementary Table 1.

- A note on how Harmony variables were defined (e.g. by sample, batch or patient) could be included.

We thank the reviewer for this comment. Harmony integration was performed using sequencing cohort batch and patient identity as covariates to account for technical and inter-patient variability. We have now clarified this in the Materials and Methods section (line 687).

Reviewer #6 (Remarks to the Author):

Reviewer Comments

The authors investigate tRCC, a relatively rare renal tumor, using a multimodal approach that includes snRNA-seq, snATAC-seq, TCR-seq, and spatial transcriptomic analyses to elucidate its cell of origin and to clarify, from the perspective of TME, why responses to ICIs tend to be poor. To the best of my knowledge, there have been no prior tRCC studies utilizing snRNA-seq and snATAC-seq, and the study design is interesting. The authors also perform analyses using publicly available datasets. However, considering the limited number of in-house cases, especially in light of pre- and post-chemotherapy sampling and the heterogeneity of fusion partners, the robustness of the conclusions may be insufficient. Furthermore, the proposed formation of a suppressive TME involving progenitor-exhausted CD8$^+$ T cells, anti-inflammatory SPP1$^+$ macrophages, and mCAFs lacks immunohistochemical validation, and the cases subjected to spatial transcriptomic analysis may not appear representative of tRCC in this study.

We thank this reviewer for the careful evaluation of our study and for recognizing the novelty and scale of our multi-modal approach for this rare tumor. We have addressed each of the reviewer's concerns in full below, including new multiplex immunofluorescence validation of the suppressive TME and additional analyses to strengthen the robustness of our conclusions. We hope the reviewer agrees that these revisions have substantially addressed the concerns raised.

While we acknowledge the limited size of the cohort, as the reviewer points out, the scale is unprecedented for this rare tumor type. Moreover, we have taken pains to perform a multi-modal characterization of our single-cell cohort together with nearly all accessible previously published bulk RNA-seq datasets and a comprehensive comparison to ccRCC. We therefore hope that this study will represent an essential and rigorously analyzed dataset for the field, within the inherent limits of tumor rarity.

1. Compared with other cohorts (PMID: 40057038), many of the cases in your study are at an advanced stage. This tendency appears to be the same even among cases that did not receive chemotherapy. I would like to know whether you intentionally selected advanced-stage cases when choosing the samples, or whether the samples were selected randomly.

We thank the reviewer for this insightful observation. We did not use any intentional stage-based criteria but rather profiled all available samples in our institutional tissue bank; details are clarified in the Methods section under "Study approval and sample collection."

As our tissue bank is organized through the Department of Medical Oncology, it is possible that there is an inherent selection for patients of advanced stage, who are treated with systemic therapy, versus those of earlier stage that may be treated by surgical resection. Upon direct comparison with the referenced study, 8 of 12 evaluable cases in our cohort (67%) were stage III/IV, compared to 9 of 16 evaluable cases (56%) in the referenced cohort[27]. While our cohort does skew slightly toward advanced disease, it is overall comparable to the referenced study. We acknowledge this as a limitation of retrospective biobank-based studies and clarified this in methods section (lines 610-612).

2. In Figure 2, the cases that underwent snRNA-seq should be clearly indicated. Given the small size of the cohort and the inclusion of both pre- and post-chemotherapy samples, specifying which cases were analyzed by snRNA-seq is crucial for assessing the validity of the study's results. If my understanding is correct that these are scRNA-seq cases that were not processed using 10x Flex, more than half of them appear to be post-chemotherapy cases. Since chemotherapy is known to affect both tumor cells and the immune microenvironment, it would be important to consider and discuss whether it may have influenced your results.

We thank the reviewer for raising this important point. Of the tRCC samples analyzed by snRNA-seq (frozen cohort, listed in Fig. 2A), 5 had received prior systemic therapy with various regimens (1 temsirolimus, 3 cabozantinib + nivolumab, 1 nivolumab followed by cabozantinib followed by lenvatinib + everolimus). Notably, none of these cases received conventional cytotoxic chemotherapy; all prior treatments were targeted therapies or immune checkpoint inhibitors. The treatments received in each case are annotated in Supplementary Table 1/Figure S1A.

Given the heterogeneous treatments received, we cannot draw strong conclusions about how prior treatments may have influenced distributions of malignant cell states or the tumor microenvironment. With respect to prior immunotherapy, as shown in Figure 4, it does appear to have an effect on T-cell states, consistent with prior

studies[28], although this will require further validation in larger tRCC cohorts (see response to Reviewer 5, comment 4).

To evaluate whether treatment status could also be associated with differences in tumor-intrinsic programs, we also checked levels of the skin pigmentation and respiration programs shown in Fig. 2, stratified by treatment-naïve or IO-exposed (as numbers were too small to interpret targeted therapy-exposed patients alone) (Rebuttal Figure 16).

Thus, within the limits of our cohort size and treatment history, we do not find substantial alterations in the dominant tumor cell programs by prior therapy.

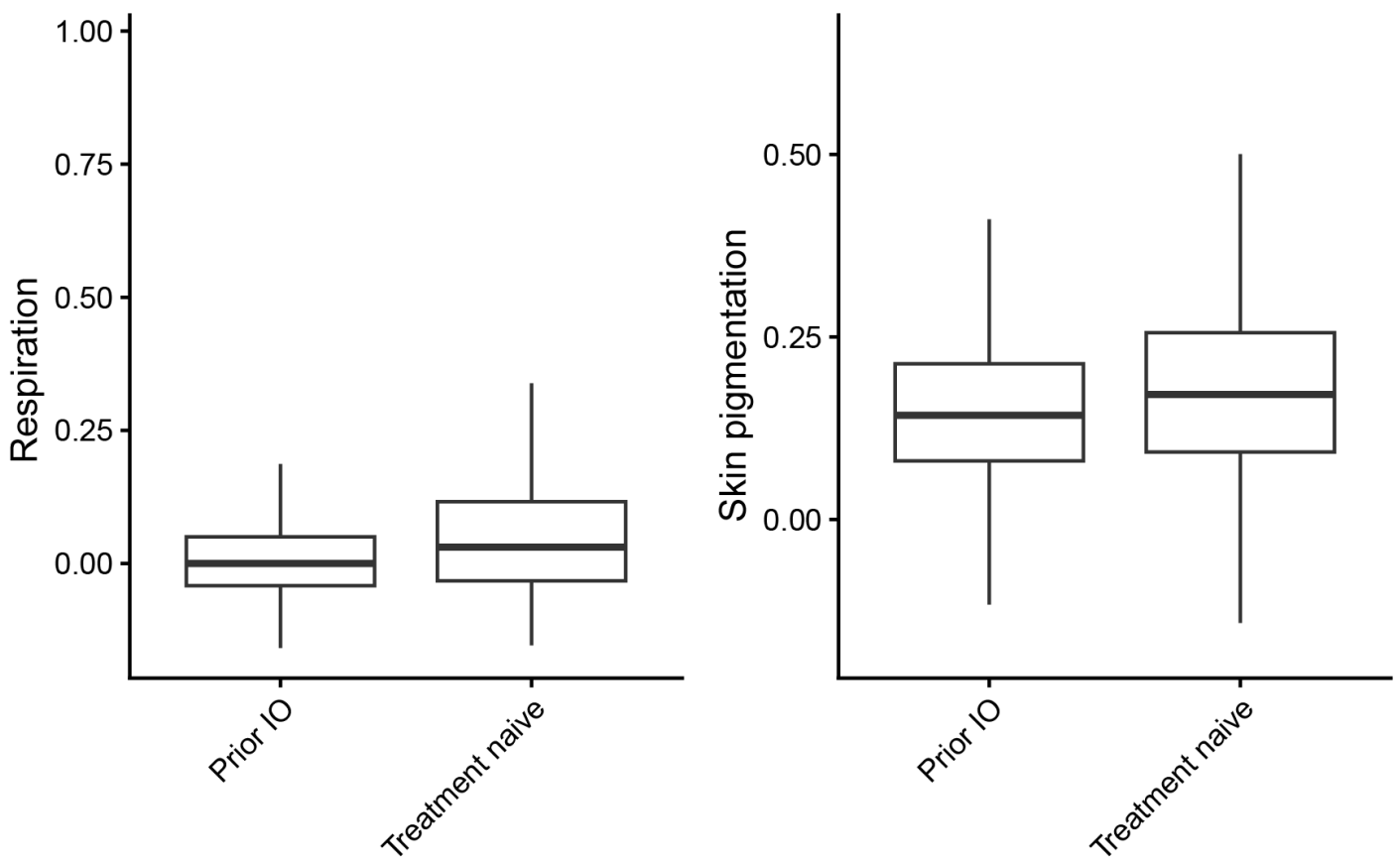

Rebuttal Figure 16. *Box plots showing average module scores for the respiration (left) and skin pigmentation (right) programs in malignant cells, stratified by prior immunotherapy exposure (Prior IO) versus treatment-naïve status in tRCC samples.*

3. Many of the genes used for the EMT signal appear to be CAF markers, so I wonder whether this can appropriately be referred to as a "tumor EMT signature." How were the genes for the EMT signal in Figure 3 selected? As key EMT markers are not included, I believe you should consider adding and analyzing representative EMT-related genes such as VIM, CDH2, S100A4, ITGA5, ITGB1, COL5A1, SNAI1/2, ZEB1/2, and TWIST1.

We thank the reviewer for this important clarification. We note that the tumor metaprogram signatures used in Figure 3 and provided in supplementary table 3 were derived empirically from NMF analysis of malignant cells from within this cohort, rather than from curated gene lists. The NMF approach is particularly useful in snRNA-seq datasets, owing the sparsity of gene expression, as many canonical marker genes from curated gene lists may not be detectable in all cells.

Briefly, we applied NMF to highly variable genes from each individual tumor, and robust gene modules were identified using a ranking strategy that retains genes most strongly contributing to a given factor. The resulting modules were then annotated biologically based on their enrichment against GO terms, KEGG pathways, Reactome pathways, Hallmark gene sets, and PanglaoDB cell type markers, with extensive manual curation.

We acknowledge the reviewer's point that some genes within the NMF-derived EMT module overlap with CAF markers, which is an inherent feature of data-driven gene module discovery rather than curated pathway analysis. However, the NMF step occurs downstream of malignant/non-malignant cell type classification. Because malignant tRCC cells were identified on the basis of highly tRCC-specific marker genes, we have a high degree of confidence that the listed genes in the EMT module are indeed expressed in malignant cells, not fibroblasts.

Importantly, we note that for all bulk cohort EMT analyses, we used the Hallmark EMT gene set from MSigDB, which includes the canonical EMT markers highlighted by the reviewer (*VIM*, *CDH2*, *S100A4*, *ITGA5*, *ITGB1*, *COL5A1*, *SNAI1/2*, *ZEB1/2*, *TWIST1*), and the enrichment of this signature in tRCC remains consistent using both gene lists.

To further address this comment, we have also reanalyzed the single-cell tumor subclusters in Figure 3D by including revised EMT and PT modules (EMT_new and PT_new) that manually incorporate 168 additional representative markers (Hallmark EMT signature), and the key trends and conclusions remain unchanged (Rebuttal Figure 17).

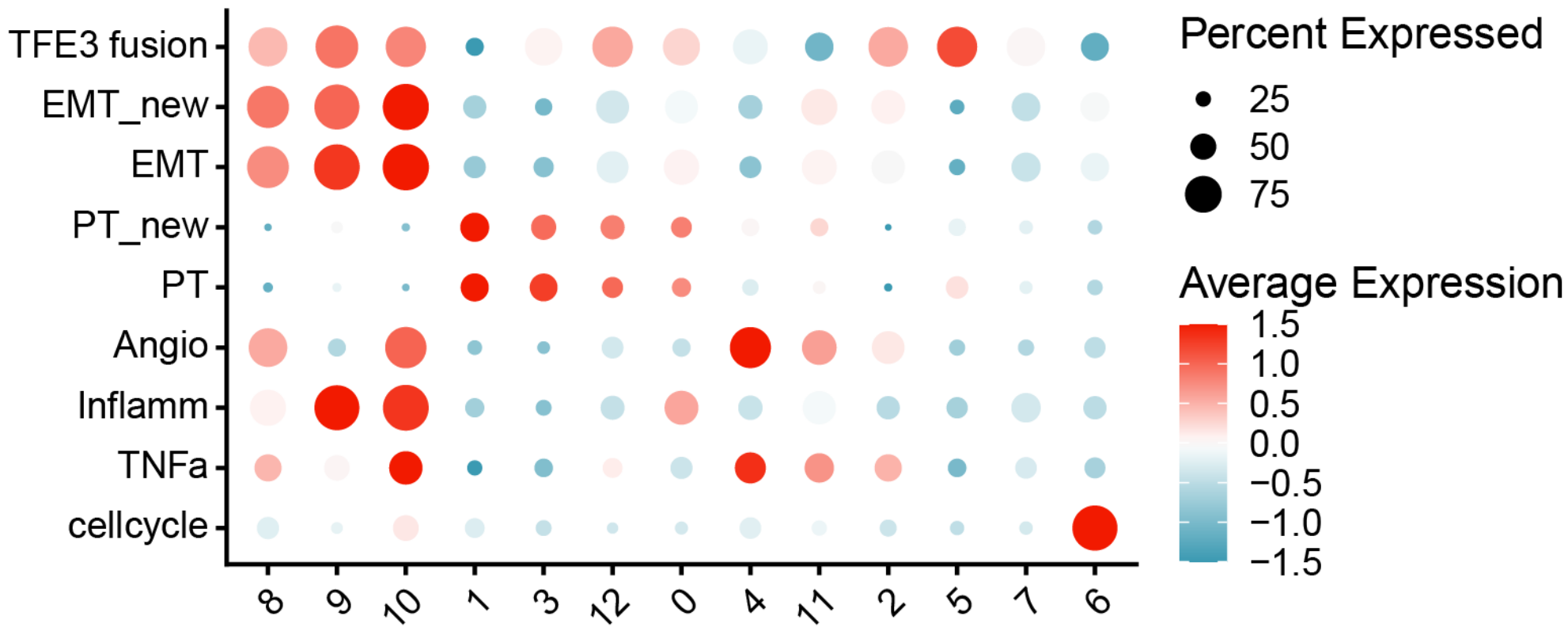

__Rebuttal Figure 17.__ Dot plot showing module scores for TFE3 fusion signature, NMF-derived EMT and PT programs (EMT, PT), revised Hallmark-based EMT and PT programs incorporating canonical markers (EMT_new, PT_new), and additional meta-programs (Angio, Inflamm, TNFa, cell cycle) across tRCC tumor subclusters. EMT_new and PT_new were derived from the MSigDB Hallmark EMT gene set, incorporating canonical markers including VIM, CDH2, S100A4, SNAI1/2, ZEB1/2, and TWIST1. Dot size represents percent of cells expressing each gene set; color represents average module score.

4. I would like to know why you chose to exclude typical PT markers such as LRP2 and AQP1.

We thank the reviewer for this question. As described above in our response to comment #3, the gene signatures used in Figure 3 were derived empirically from NMF analysis of malignant cells rather than selected *a priori*. Canonical proximal tubule markers such as LRP2 and AQP1 were not excluded intentionally, rather, they were not identified as highly variable genes within the malignant cell population in our dataset and therefore did not contribute to any NMF-derived module. To address the reviewer's concern, we have also generated a revised PT module (PT_new, Rebuttal Figure 17, above) that incorporates additional representative PT markers, and the overall trends in Figure 3D remain consistent with our original findings.

5. In Figure 4E, the comparison between ICI and non-ICI cases is based on only a single ICI-treated case, so the conclusions that can be drawn from these data appear to be weak. In addition, while Figure 4E suggests that the CD8+c1 subtype is more frequent in the ICI group, Figure 4F shows that CD8$^+$ T cells separate cleanly into ICI and non-ICI groups, which is difficult to interpret.

We thank the reviewer for this important comment. Regarding Figure 4E, we agree that with only one ICI-treated sample in the 10x Flex cohort, no meaningful statistical comparison can be performed, and the observed enrichment of progenitor-exhausted CD8+ T cells in the ICI-exposed sample represents a descriptive trend only. We have revised the text to make this limitation explicit. Importantly, this observation is corroborated by the independent frozen snRNA-seq cohort (N=10 samples, 4 ICI-treated), where CD8+ T cells cluster into three subpopulations (Figure 4F); clusters 0 and 1 are predominantly derived from ICI-naïve tumors while cluster 2, which displays the highest progenitor-exhausted signature, is enriched in ICI-treated samples (Figure 4G). The convergence of this finding across two independent cohorts using orthogonal profiling platforms provides support for this observation despite the limited sample size in the FFPE cohort.

Regarding Figure 4F, the separation of CD8+ T cells by ICI status on the UMAP reflects the fact that ICI-treated and ICI-naïve samples contribute differentially to the three CD8+ subclusters rather than forming entirely distinct transcriptional populations, cluster 2 is enriched in ICI-treated samples, while clusters 0 and 1 are predominantly ICI-naïve, as shown in the ICI status overlay and confirmed by the progenitor exhaustion signature in Figure 4G. We have addressed the limitations of our analyses and results revised text (lines 350-356).

6. In ccRCC, SPP1$^+$ macrophage infiltration has been reported (PMID: 36423636), and these cells have been implicated in CD8$^+$ T-cell dysfuncton(PMID: 40680256). I believe it would be appropriate to explicitly acknowledge their role in the immunosuppressive microenvironment of ccRCC and then discuss tRCC in that context. In addition, how do you explain the absence of SPP1$^+$ macrophages in ccRCC in Figure 5B?

We fully agree with the reviewer on this important point. We have revised the Discussion (lines 511-516) to recognize SPP1+ macrophages as an emerging immunosuppressive hub across multiple tumor types including ccRCC, and to contextualize our tRCC findings within this broader framework.

Regarding the apparent absence of SPP1+ macrophages in ccRCC in Figure 5B, we wish to clarify that this figure reflects the macrophage cluster composition in the Wu et al. frozen snRNA-seq dataset[5], which we did not generate ourselves. While SPP1 transcripts are detectable at low levels within macrophage populations in this dataset, the expression was insufficient to define a distinct SPP1+ macrophage cluster using our clustering and annotation approach. While such a dramatic difference is most likely primarily due to biological differences between tRCC and ccRCC, as supported by multiple lines of orthogonal data in our manuscript, we cannot exclude differences due to patient population and sample processing that may also have contributed. These caveats have been more explicitly stated in the text (lines 511-516). Importantly, Figure 5B was intended to illustrate the relative enrichment of SPP1+ macrophages in tRCC compared to ccRCC rather than to imply their complete absence in ccRCC (which we have also validated using multiplex immunohistochemistry: Rebuttal Figure 12 and Figures 5C-D in revised manuscript). We propose that this quantitative enrichment of SPP1+ macrophages, rather than a binary presence/absence distinction, contributes to the more suppressive TME and reduced immunotherapy responsiveness observed in tRCC relative to ccRCC. The role of SPP1+ macrophages in suppressing CD8+ T cell activity, as recently demonstrated in ccRCC[29], is likely amplified in tRCC given their greater abundance, and this is now explicitly discussed in the revised manuscript.

7. The cases shown in Figures 5E and 5F are post-chemotherapy, and I'm not sure whether a case treated with an mTOR inhibitor can be a representative example. The case in Figure 5G also harbors a LUC7L3 fusion, which is a very rare subtype even among tRCC. In light of these points, can the findings derived from these cases truly be generalized to tRCC as a whole? In addition, SPP1$^+$ macrophages appear to be quite abundant; however, histologically it is not generally recognized that tRCC is characterized by prominent macrophage infiltration. Moreover, SPP1$^+$ macrophages themselves seem to represent only a relatively small subset. Could you demonstrate the colocalization of SPP1 with macrophage markers by multiplex staining, for example using CD68 or other macrophage markers in the other cases?

We thank the reviewer for these thoughtful comments. Regarding the representativeness of the spatial cases, we wish to clarify that the CosMx spatial transcriptomics data serve as orthogonal spatial validation of interactions first identified computationally across all samples in both our frozen and 10X Flex snRNA-seq cohorts. The primary biological conclusions rest on this cohort-wide snRNA-seq analysis rather than on the two spatial cases alone. We acknowledge that TRCC6 received prior mTOR inhibitor therapy and TRCC14 harbors the comparatively uncommon *LUC7L3-TFE3* fusion. We have added explicit annotation of these facts to the figure legends. However, the generalizability of our findings is supported by consistent identification of SPP1+ macrophages as the dominant myeloid population across all tRCC samples spanning multiple fusion partners and treatment histories in the snRNA-seq cohorts, orthogonal validation by multiplex IF, correlation with EMT score across 144 bulk RNA-seq tRCC profiles from five independent cohorts, and by independent prognostic significance in multivariate survival analysis.

We also wish to clarify that, regarding SPP1+ macrophage infiltration, our claim is not that tRCC is characterized by abundant macrophage infiltration overall, but that within the macrophage compartment, SPP1+ macrophages are specifically enriched relative to ccRCC. In support of this, our multiplex immunofluorescence data confirm significantly higher CD163+SPP1+ macrophages in tRCC ($p=0.0083$) while CD163+MRC1+ macrophages did not differ significantly between tumor types. This demonstrates that the enrichment is specific to the SPP1+ subpopulation rather than reflecting a generalized expansion of M2-polarized macrophages (Rebuttal Figure 12; Figures 5C-D in revised manuscript).

8. Please correct the label in Supplementary Figure 3B from "Primary tumor nephrectomy" to "Primary tumor nephrectomy."

Thank you. The label in Supplementary Figure 3B now reads "Primary tumor nephrectomy."

9. For case TRCC22, the stage is listed as IV, while the pathological status is given as pT3N0, and there is no description of distant metastasis. It would be better to clarify whether distant metastasis was present (Stage IV) or whether the correct stage is Stage III.

We thank the reviewer for pointing this out. TRCC22 presented with distant metastasis at the time of primary tumor staging; the stage has been updated to pT3N0M1 in the revised Supplementary Table 1 to accurately reflect Stage IV disease.

10. In the Results section, within the subsection "Suppressive macrophages and fibroblasts in the tRCC TME," there is a sentence that ends with a comma instead of a period. Please correct this.

We thank the reviewer for catching this error. The comma has been corrected to a period in the revised manuscript.

11. Please check the spelling of “temsirolimus” in Supplementary Table 1.

Thank you. The spelling of "temsirolimus" has been corrected in the revised Supplementary Table 1.

REFERENCES

1. Chen, F. *et al.* Multilevel Genomics-Based Taxonomy of Renal Cell Carcinoma. *Cell Rep.* **14**, 2476–2489 (2016).
2. Salem, S., Alchoueiry, M., Xu, W. & Henske, E. P. Chromophobe renal cell carcinoma: Emerging vulnerabilities as a promise for a new therapeutic landscape. *Crit. Rev. Oncol. Hematol.* **216**, 104985 (2025).
3. Li, B. *et al.* Comprehensive analyses of tumor immunity: implications for cancer immunotherapy. *Genome Biol.* **17**, 174 (2016).
4. Newman, A. M. *et al.* Determining cell type abundance and expression from bulk tissues with digital cytometry. *Nat. Biotechnol.* **37**, 773–782 (2019).
5. Wu, Y. *et al.* Epigenetic and transcriptomic characterization reveals progression markers and essential pathways in clear cell renal cell carcinoma. *Nat. Commun.* **14**, 1681 (2023).
6. Zhang, Y. *et al.* Single-cell analyses of renal cell cancers reveal insights into tumor microenvironment, cell of origin, and therapy response. *Proc. Natl. Acad. Sci.* **118**, e2103240118 (2021).
7. Lake, B. B. *et al.* A single-nucleus RNA-sequencing pipeline to decipher the molecular anatomy and pathophysiology of human kidneys. *Nat. Commun.* **10**, 2832 (2019).
8. Kiuchi-Saishin, Y. *et al.* Differential expression patterns of claudins, tight junction membrane proteins, in mouse nephron segments. *J. Am. Soc. Nephrol. JASN* **13**, 875–886 (2002).
9. Schumacher, A. *et al.* Defining the variety of cell types in developing and adult human kidneys by single-cell RNA sequencing. *Npj Regen. Med.* **6**, 45 (2021).
10. D'Agati, V. D. & Shankland, S. J. Recognizing diversity in parietal epithelial cells. *Kidney Int.* **96**, 16–19 (2019).
11. Kuppe, C. *et al.* Novel parietal epithelial cell subpopulations contribute to focal segmental glomerulosclerosis and glomerular tip lesions. *Kidney Int.* **96**, 80–93 (2019).
12. Troulé, K. *et al.* CellPhoneDB v5: inferring cell-cell communication from single-cell multiomics data. *Nat. Protoc.* **20**, 3412–3440 (2025).
13. broadinstitute. InferCNV. *GitHub* https://github.com/broadinstitute/infercnv/wiki/Home.

14. Achom, M. *et al.* A genetic basis for sex differences in Xp11 translocation renal cell carcinoma. *Cell* **187**, 5735-5752.e25 (2024).

15. Neftel, C. *et al.* An Integrative Model of Cellular States, Plasticity, and Genetics for Glioblastoma. *Cell* **178**, 835-849.e21 (2019).

16. Tirosh, I. *et al.* Dissecting the multicellular ecosystem of metastatic melanoma by single-cell RNA-seq. *Science* **352**, 189–196 (2016).

17. Aiello, N. M. *et al.* EMT Subtype Influences Epithelial Plasticity and Mode of Cell Migration. *Dev. Cell* **45**, 681-695.e4 (2018).

18. Youssef, K. K. *et al.* Two distinct epithelial-to-mesenchymal transition programs control invasion and inflammation in segregated tumor cell populations. *Nat. Cancer* **5**, 1660–1680 (2024).

19. Lo, H. C. & Zhang, X. H.-F. EMT in Metastasis: Finding the Right Balance. *Dev. Cell* **45**, 663–665 (2018).

20. Gao, Y. *et al.* Metastasis Organotropism: Redefining the Congenial Soil. *Dev. Cell* **49**, 375–391 (2019).

21. Kiani, K., Sanford, E. M., Goyal, Y. & Raj, A. Changes in chromatin accessibility are not concordant with transcriptional changes for single-factor perturbations. *Mol. Syst. Biol.* **18**, e10979 (2022).

22. Klemm, S. L., Shipony, Z. & Greenleaf, W. J. Chromatin accessibility and the regulatory epigenome. *Nat. Rev. Genet.* **20**, 207–220 (2019).

23. Tu, Z. *et al.* Discordance between chromatin accessibility and transcriptional activity during the human primed-to-naïve pluripotency transition process. *Cell Regen.* **12**, 35 (2023).

24. Sun, G. *et al.* Integrated exome and RNA sequencing of TFE3-translocation renal cell carcinoma. *Nat. Commun.* **12**, 5262 (2021).

25. Asrani, K. *et al.* SFPQ-TFE3 reciprocally regulates mTORC1 and induces lineage plasticity in a mouse model of renal tumorigenesis. *Nat. Commun.* **16**, 8822 (2025).

26. Prakasam, G. *et al.* Comparative genomics incorporating translocation renal cell carcinoma mouse model reveals molecular mechanisms of tumorigenesis. *J. Clin. Invest.* **134**, (2024).

27. Liu, H. *et al.* Molecular Characterization of TFE3-Rearranged Renal Cell Carcinoma in Children and Adolescents. *Mod. Pathol. Off. J. U. S. Can. Acad. Pathol. Inc* **38**, 100748 (2025).

28. Miller, B. C. *et al.* Subsets of exhausted CD8+ T cells differentially mediate tumor control and respond to checkpoint blockade. *Nat. Immunol.* **20**, 326–336 (2019).

29. Jiang, W. *et al.* SPP1+ Tumor-Associated Macrophages Drive Immunotherapy Resistance via CD8+ T-cell Dysfunction in Clear-Cell Renal Cell Carcinoma. *Cancer Immunol. Res.* **13**, 1533–1546 (2025).
